# Co‐Catalyzed Synthesis of Primary Amines via Reductive Amination employing Hydrogen under very mild Conditions

**DOI:** 10.1002/cssc.202100553

**Published:** 2021-05-06

**Authors:** Matthias Elfinger, Timon Schönauer, Sabrina L. J. Thomä, Robert Stäglich, Markus Drechsler, Mirijam Zobel, Jürgen Senker, Rhett Kempe

**Affiliations:** ^1^ Inorganic Chemistry II – Catalyst design Sustainable Chemistry Centre University of Bayreuth 95440 Bayreuth Germany; ^2^ Solid State Chemistry – Mesostructured Materials University of Bayreuth 95440 Bayreuth Germany; ^3^ Inorganic Chemistry III and North Bavarian NMR center University of Bayreuth 95440 Bayreuth Germany; ^4^ Bavarian Polymer Institute (BPI) Keylab “Electron and Optical Microscopy” University of Bayreuth 95440 Bayreuth Germany

**Keywords:** catalysis, cobalt, ketones, primary amines, reductive amination

## Abstract

Nanostructured and reusable 3d‐metal catalysts that operate with high activity and selectivity in important chemical reactions are highly desirable. Here, a cobalt catalyst was developed for the synthesis of primary amines via reductive amination employing hydrogen as the reducing agent and easy‐to‐handle ammonia, dissolved in water, as the nitrogen source. The catalyst operates under very mild conditions (1.5 mol% catalyst loading, 50 °C and 10 bar H_2_ pressure) and outperforms commercially available noble metal catalysts (Pd, Pt, Ru, Rh, Ir). A broad scope and a very good functional group tolerance were observed. The key for the high activity seemed to be the used support: an N‐doped amorphous carbon material with small and turbostratically disordered graphitic domains, which is microporous with a bimodal size distribution and with basic NH functionalities in the pores.

## Graphical abstract

**Reductive amination**: A nanostructured highly active cobalt catalyst is employed for the reductive amination of ketones and aldehydes by ammonia and H_2_ under very mild conditions. A broad substrate scope and very good functional group tolerance are observed. Detailed analysis of the catalyst's chemical and physical nature provides key reasons for its extraordinary activity, outperforming noble metal catalysts.

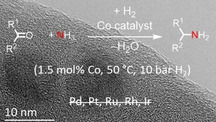

## Introduction

**Scheme 1:**
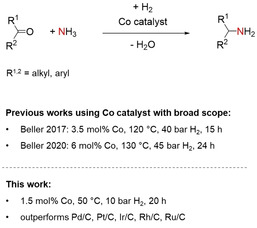
Reusable and nanostructured Co catalysts for the reductive amination of ketones and aldehydes to primary amines using hydrogen as the reducing agent.

Reductive amination, the reaction of an aldehyde or a ketone and ammonia or an amine in the presence of a reducing agent, is a very important chemical transformation intensively investigated and used in industry and academia.^[1]^ The use of inexpensive and potentially green hydrogen as the reducing agent in the presence of a catalyst is especially attractive.^[1]^ The products of reductive aminations, alkyl amines, are very important bulk and fine chemicals and/or biologically active compounds such as pharmaceuticals or agro chemicals.^[2]^ Especially challenging and important is the synthesis of primary amines. The reaction is challenging since over‐alkylation and other unwanted side reactions have to be avoided, and it is crucial since primary amines are important starting materials to synthesize other amines or N‐heterocyclic compounds.^[3]^ The replacement of rare elements in key technologies is very important too. Catalysis, a key technology of our century, is often based on rare noble metals such as Pd, Pt, Rh, and Ir. Earth‐abundant metal catalysts have played an important role with respect to the development of reductive amination in its early days,^[4]^ in particular Raney Ni. Note that Raney Ni is difficult to handle and its reusability is limited. Very recently, nanostructured and reusable Co catalysts have been introduced for the general synthesis of primary amines from ammonia and ketones or aldehydes and hydrogen (Scheme 1).^[5]^ Unfortunately, these catalysts operate under rather harsh conditions. A general problem with 3d‐metal catalysts is their significantly lower activity in many reactions in comparison to noble metal catalysts. Thus, reusable or nanostructured catalysts based on abundantly available elements that operate under very mild conditions and are competitive with noble metal catalysts are highly desirable. We have recently introduced Ni^[6]^ and Fe^[7]^ catalysts and report here on a highly active and reusable Co catalyst for the selective synthesis of primary amines via reductive amination employing hydrogen as the reducing agent. Our catalyst operates under very mild conditions (1.5 mol% catalyst loading, 50 °C and 10 bar H_2_ pressure) and outperforms commercially available noble metal catalysts (Pd, Pt, Ru, Rh, Ir), and ammonia dissolved in water, which is easy to handle and inexpensive, is used. We observe a broad scope with good functional group tolerance that encompasses aryl‐alkyl, diaryl, dialkyl ketones and aldehydes, which can be converted into the corresponding primary amines. Furthermore, the amination of biologically active compounds has been demonstrated. The key for the high activity seems to be the used N‐SiC support. It is an N‐doped (≈7 wt%) amorphous carbon material processed at 1000 °C having graphitic domains smaller than 2 nm with a turbostratic disorder and widened average interlayer distances. The material is microporous with a bimodal pore size distribution, which look as if to be of a chemically different origin. The larger of them seem to contain accessible basic NH functionalities most likely formed via hydrolysis of C−N−Si bonds during NaOH treatment. Besides the above‐mentioned two Co catalysts for the synthesis of primary amines from ketones or aldehydes and ammonia, other Co catalyst systems for reductive amination reactions employing hydrogen as the reducing agent have been introduced.^[8]^

## Results and Discussion

### Catalyst synthesis and characterization

**Figure 1:**
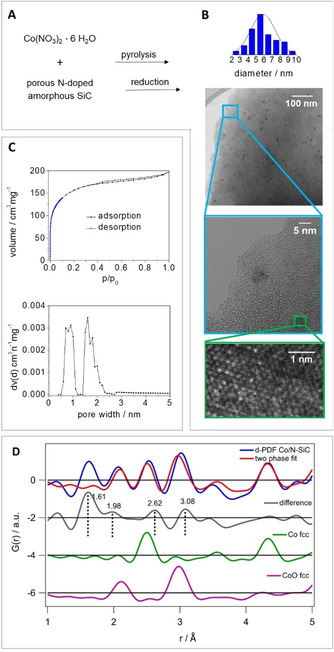
Synthesis and characterization of the Co catalyst: (A) Synthesis of the crosslinked material, followed by pyrolysis and NaOH treatment to obtain the support material; wet impregnation, pyrolysis and reduction led to the active Co catalyst. (B) TEM analysis suggested the presence of homogeneously distributed Co nanoparticles with an average particle size of 5.9 nm. High‐resolution (HR)TEM micrograph with atomic resolution of a Co nanoparticle surface. (C) Surface characterization and pore size distribution of the catalyst via argon physisorption measurement [calculation model: Ar at 87 K on carbon (cylindr. pores, NLDFT equilibrium model)]. The specific surface showed a slight decline from 545 m^2^ g^−1^ of the support material to 491 m^2^ g^−1^ of the catalyst. (D) The differential‐PDF of (Co/N‐SiC)‐(N‐SiC) (blue) was fitted with a two‐phase fit (red) containing a Co fcc (green) and a CoO fcc (pink) phase. Remaining distance correlations in the difference curve (grey) exist for the short‐range order up to 5 Å.

First, the N‐SiC support was synthesized following published procedures by crosslinking the commercially available polycarbosilane precursor SMP 10 and acrylonitrile in dimethylformamide, using azobis‐isobutyronitrile as an initiator.^[9]^ The greenbody was pyrolyzed at 1000 °C under inert atmosphere and then treated with 1 m NaOH at 85 °C overnight (see the Supporting Information 2.1). To obtain the active catalyst, the N−SiC support was impregnated with Co(NO_3_)_2_ ⋅ 6H_2_O dissolved in water, pyrolyzed (700 °C, N_2_) and reduced (550 °C, N_2_/H_2_). Inductively coupled plasma optical emission spectroscopy (ICP‐OES) analysis of the catalyst revealed a cobalt content of 2.24 wt% (see the Supporting Information 3.1). The specific surface area could be determined by argon physisorption measurements (Figure 1C) and showed a decline in surface area from 545 m^2^ g^−1^ of the support material to 491 m^2^ g^−1^ of the catalyst. A homogeneous distribution of the cobalt nanoparticles over the N‐SiC support (Figure 1B) and an average particle size of 5.9 nm could be determined via transmission electron microscopy (TEM). In addition, X‐ray photoelectron spectroscopy (XPS) showed the presence of metallic cobalt and cobalt oxi(hydroxide) species as well as different binding modes of nitrogen within the support material at the surface of the catalyst material (see the Supporting Information Figure S2). Scanning electron microscopy (SEM) in combination with energy dispersive X‐ray spectroscopy (EDX) confirmed the homogeneous distribution of the cobalt nanoparticles over the N‐SiC support (see the Supporting Information Figure S3). Pair distribution function (PDF) studies (Figure 1D) indicate the presence of Co fcc and CoO fcc nanoparticles. The Co domain size was refined to about 9 nm in comparison to the CoO domain size with about 3 nm (see the Supporting Information Table S3). An average particle diameter of 5.9 nm with a distribution from 3–9 nm was determined by TEM (Figure 1B). This suggests that separate Co and CoO nanoparticles exist rather than Co and CoO domains within one particle.

### Optimization of the reaction conditions

In order to determine the optimized reaction conditions, the reductive amination of acetophenone to 1‐phenylethylamine was chosen as benchmark reaction. The solvent screening revealed that a concentration of ammonia in water of 32 % leads to the best yield of amine product since the high concentration accelerates the imine formation prior to the reduction step. The optimally adjusted reaction parameters could be defined as 3.5 mL aqueous ammonia (32 %), 10 bar H_2_, 50 °C and 1.5 mol% cobalt. Since water is our preferred solvent, ammonia gas could also be used, which would form ammonia dissolved in water then. We further investigated the influence of varying metal loadings of the catalyst (Table 1). The experiments revealed that the catalyst with theoretically 3 wt% cobalt showed the best activity in the reductive amination. Lowering the pyrolysis temperature to 600 °C led to a significant reduction of yield. Applying a higher temperature (800 °C) led to better results than 600 °C but could not match the superior activity of the catalyst that was pyrolyzed at 700 °C. Pure N‐SiC support as well as cobalt nitrate on various commercially available supports showed no conversion of acetophenone. TEM investigation of these materials revealed inhomogeneously distributed or barely present nanoparticles (see the Supporting Information Figure S4). A highly active Ni/Al_2_O_3_ catalyst introduced by our group in 2019 also showed no activity when applied under the conditions mentioned above.^[6]^ For this Ni‐catalyst, a Ni complex stabilized by a N,O‐ligand called salen was used as a metal precursor and the C‐ and N‐atoms of the ligand provide an N‐doped carbon matrix in which the Ni nanoparticles are embedded and, so, fixed at the Al_2_O_3_ support. It might be that the very mild reaction conditions make it very challenging for other supports in combination with inexpensive simple metal precursor Co nitrate. When comparing the activity and selectivity of well‐known and commercially available noble metal catalysts to our cobalt system, it can be shown that the latter is outperforming all noble catalysts applying the same reaction conditions. Ru/C and Rh/C are inactive regarding the formation of the primary amine **1**. Other metals (Pd, Pt, and Ir) form the desired product of about 50 % and simultaneously side products based on acetophenone hydrogenation mainly 1‐phenylethanol (**2**) and 1‐phenyl‐cyclohexylalcohol (**3**) (Table 2). When compared to the literature where the selective reductive amination of ketones to branched primary amines using ammonia is addressed, all papers except one report harsher conditions in terms of temperature and hydrogen pressure than we have used for our cobalt catalyst system.^[10]^ In 2020, Wang and co‐workers reported on palladium nanoparticles for the reductive amination of 16 aromatic ketones with aqueous ammonia under milder conditions.^[11]^ However, extremely high catalyst loadings of 37 mol% Pd were used.

**Table 1:** Cobalt catalyst comparison.^[a]^

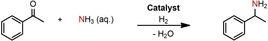				

Entry	Metal source	Support	Pyrolysis temperature [°C]	Yield= Conversion [%]
1	Co‐nitrate (3 wt% Co)	N‐SiC	700	99
2	Co‐nitrate	N‐SiC	600	3
3	Co‐nitrate	N‐SiC	800	87
4	Co‐nitrate (2 wt% Co)	N‐SiC	700	21
5	Co‐nitrate (4 wt% Co)	N‐SiC	700	91
6	Co‐nitrate (5 wt% Co)	N‐SiC	700	32
7	Co‐nitrate	activated C	700	0
8	Co‐nitrate	Al_2_O_3_	700	0
9	Co‐nitrate	TiO_2_	700	0
10	Co‐nitrate	SiO_2_	700	0
11	–	N‐SiC	700	0
12^[b]^	Ni salen complex	Al_2_O_3_	700	0

[a] Reaction conditions: 1.5 mol% Co (2.24 wt% Co, 0.0075 mmol Co, 0.44 mg Co), 0.5 mmol acetophenone, 50 °C, 20 h, 10 bar H_2_, 3.5 mL aq. NH_3_‐32 %. Yields were determined by GC using *n*‐dodecane as an internal standard. [b] 2.0 mol% Ni (4.0 wt% Ni).

**Table 2:** Reductive amination with noble metal catalysts.^[a]^

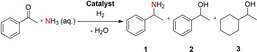					

Entry	Catalyst	Yield 1 [%]	Yield 2 [%]	Yield 3 [%]	Other products [%]
13^[b]^	Pd/C	46	53	–	–
14^[c]^	Pt/C	48	32	–	19
15^[d]^	Ru/C	3	58	6	32
16^[e]^	Rh/C	0	0	48	51
17^[f]^	Ir/C	49	30	–	20

[a] Reaction conditions: 0.5 mmol acetophenone, 50 °C, 20 h, 10 bar H_2_, 3.5 mL aq. NH_3_‐32 %. Yields were determined by GC using *n*‐dodecane as an internal standard. [b] 2.0 mol% Pd (10.0 wt% Pd). [c] 2.0 mol% Pt (5.0 wt% Pt). [d] 2.0 mol% Ru (5.0 wt% Ru). [e] 2.0 mol% Rh (5.0 wt% Rh). [f] 2.0 mol% Ir (1.0 wt% Ir).

### Substrate scope

Since the optimized reaction conditions have been predefined, we were interested in the substrate scope of our highly active/selective catalyst system. The product yields refer to the isolated corresponding hydrochloride salts of the synthesized amines.

**Scheme 2:**
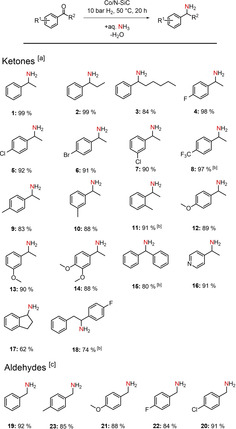
Reductive amination of aryl substituted ketones and aldehydes to primary amines. [a] Reaction conditions for ketones: 1.5 mol% Co (2.24 wt% Co, 0.0075 mmol Co, 0.44 mg Co), 0.5 mmol ketone, 50 °C, 20 h, 10 bar H_2_, 3.5 mL aq. NH_3_‐32 %. [b] 60 °C. [c] Reaction conditions for aldehydes: 1.1 mol% Co, 0.5 mmol aldehyde, 80 °C, 20 h, 10 bar H_2_, 3.5 mL aq. NH_3_‐32 %. Isolated yields of the converted hydrochloride salts.

First, we investigated the reductive amination of aryl‐alkyl ketones with various substituents. Substrates with short, moderate and long aliphatic moieties (Scheme 2, products **1**–**3**) could be isolated in very good yields of 84–99 %. The conversion of ketones with electron‐withdrawing substituents, such as halogens, also worked out well with yields of over 90 % for the *para*‐substituted derivatives (Scheme 2, products **4**–**6**). The sterically more demanding *meta*‐chloroacetophenone (Scheme 2, product **7**) could also be converted with an isolated yield of 90 %. The heavily electron‐withdrawing trifluoromethyl group (Scheme 2, product **8**) required higher reaction temperatures (60 °C) to be converted into its corresponding primary amine with an excellent yield of 97 %. Minor electron‐donating substituents, such as methyl groups, could be tolerated very well in *meta*‐ and *para*‐positions of the aromatic ring (Scheme 2, products **9** and **10**). For the *ortho*‐substituted acetophenone, we had to increase the temperature to 60 °C to obtain 91 % of isolated product (Scheme 2, product **11**). If the electron‐donating property of the substituents was increased (e. g., methoxy groups), *meta*‐ and *para*‐substituted ketones (Scheme 2, products **12** and **13**), as well as dimethoxy acetophenone (product **14**) could be converted smoothly into the corresponding primary amines without any need to increase the temperature. The reductive amination of benzophenone (Scheme 2, product **15**) showed that aryl‐aryl ketones can also be converted with slightly harsher conditions, which are also needed for the sterically demanding benzyl‐4‐fluorophenyl ketone (product **18**). The amination of a N‐heterocyclic ketone (Scheme 2, product **16**) proceeded smoothly with an isolated yield of 91 %. The corresponding amine of 1‐indanon could be acquired with a 62 % yield (product **17**). For the reductive amination of benzylic aldehydes, higher temperatures were needed to suppress the self‐coupling reaction of the primary amine product with unconverted substrate. Therefore 80 °C and a catalyst loading of 1.1 mol% were used. Benzaldehyde (Scheme 2, product **19**), as well as its substituted derivates could be converted in very good yields. Electron‐donating substituents such as methyl and methoxy groups (Scheme 2, products **20** and **21**) were tolerated very well with isolated yields of the corresponding amines up to 88 %. In addition, the conversion of substituted benzaldehydes containing electron‐withdrawing moieties such as halogens was also accomplished with very good yields up to 91 % (Scheme 2, products **22** and **23**).

**Scheme 3:**
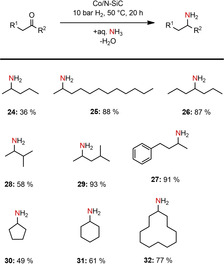
Reductive amination of purely aliphatic ketones to primary amines. Reaction conditions: 1.5 mol% Co (2.24 wt% Co, 0.0075 mmol Co, 0.44 mg Co), 0.5 mmol ketone, 50 °C, 20 h, 10 bar H_2_, 3.5 mL aq. NH_3_‐32 %. Isolated yields of the converted hydrochloride salts.

In addition, we investigated the reductive amination of purely aliphatic ketones. Substrates with long and short branched and linear aliphatic chains as well as cyclic ketones could be aminated in moderate to very good yields (36–93 %, Scheme 3). Aliphatic ketones with low molecular weight could only be isolated in moderate yields due to their low boiling points, which caused significant losses of product during the workup procedure.

**Scheme 4:**
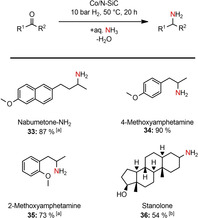
Reductive amination of biologically active molecules to primary amines. Reaction conditions:1.5 mol% Co (2.24 wt% Co, 0.0075 mmol Co, 0.44 mg Co), 0.5 mmol ketone, 50 °C, 20 h, 10 bar H_2_, 3.5 mL aq. NH_3_‐32 %; Isolated yields of the converted hydrochloride salts. [a] 60 °C; [b] 3 mol% Co, 65 °C, 3.2 mL aq. NH_3_‐32 %, 0.3 mL EtOH.

Next, we became interested in the reductive amination of biologically active molecules under very mild conditions. The selective conversion of nabumetone, 4‐ and 2‐methoxyphenylacetone to their corresponding primary amines could be accomplished in isolated yields of up to 90 % (Scheme 4). The reductive amination of steroid molecules was rather challenging, due to the poor water solubility of this substance class at 50 °C. By applying 65 °C and 3 mol% Co as well as adding 0.3 mL of ethanol to enhance the solubility, 54 % of Stanolone‐NH_2_ could be obtained. Attempts to aminated examples carrying an ester, an amide, or a nitro group and a keto acid failed under the given conditions.

### Reusability and upscaling

Finally, we examined the reusability and upscaling of our cobalt catalyst system. Five consecutive runs without any loss of activity were performed to confirm the recyclability (see the Supporting Information Figure S1), and the reductive amination of 10 mmol acetophenone, our model substrate, could be accomplished with 95 % yield (see the Supporting Information 2.4.3).

### Nature of the N‐SiC support

**Figure 2:**
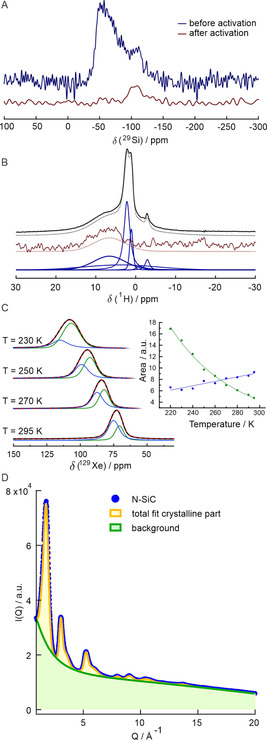
NMR and PXRD/PDF investigations. (A) ^29^Si SP MAS NMR spectra of the N‐SiC support prior (blue) and after (brown) NaOH treatment (activation). (B) Ultrafast ^1^H MAS NMR spectra; SP excitation (top) and nitrogen selective ^1^H‐^14^N HMQC excitation (brown). Both spectra are deconvoluted with the same set of normal components (blue lines). The resulting lineshapes are depicted by pointed lines beneath the experimental spectra (C) Variable‐temperature ^129^Xe NMR spectra using hyperpolarized ^129^Xe within the limiting shift region. The inset depicts the intensity trend for the observed two resonances as function of temperature. (D) PXRD data of N‐SiC support material (blue) with its fit for the crystalline phase fraction (yellow) of 17 % on the big amorphous background (green).

The key for the high activity seems to be the N‐SiC support, which we analyzed in detail employing solid‐state NMR spectroscopy (ssNMR), synchrotron radiation‐based powder X‐ray diffraction (PXRD)/PDF, Raman spectroscopy, XPS, and acid‐base titration. Key results of the NMR spectroscopic and PXRD/PDF investigations are listed in Figure 2. The starting point of the support synthesis is a copolymerization of acrylonitrile with the commercially available polycarbosilane SMP 10 followed by pyrolysis up to 1000 °C. The pyrolyzed material is then treated with a 1 m NaOH solution at 85 °C for 16 h. ^13^C cross polarization (CP) and ^29^Si single pulse magic angle spinning (SP MAS) NMR spectra (see the Supporting Information Figure S12 and Figure 2A) suggest that the pyrolyzed material consists of graphitic domains and silicon rich regions with mixed SiC_*x*_O_4‐*x*_ and SiN_*x*_O_4‐*x*_ environments.[^9^, ^12^] The NaOH treatment leads to a nearly complete removal of the silicon‐rich domains. In this process, the specific surface area increases from 6 to 545 m^2^ g^−1^ [Ar sorption, Brunauer‐Emmett‐Teller (BET) model, see the Supporting Information Figure S5]. The pore size distribution below 2.3 nm supports a domain structure of the pyrolyzed material, which is turned into a predominantly microporous material by the NaOH treatment. Besides adsorbed mobile water, the ^1^H NMR spectra obtained with ultrafast MAS and SP (black) as well as nitrogen‐selective ^1^H‐^14^N heteronuclear multiple quantum coherence (HMQC) excitation (brown) (Figure 2B) prove the presence of a substantial amount of NH_*x*_ (*x*=1, 2) functionalities.^[13]^ The latter is also supported by the ^15^N{^1^H} CP MAS NMR spectra (see the Supporting Information Figure S14) and the downfield flank within the ^13^C MAS NMR spectrum supportive for carbon atoms in local CN_*y*_(NH_*x*_) environments also observed for graphitic carbon nitrides.^[14]^ The N 1s XPS spectra (see the Supporting Information Figure S2D) are in agreement with this analysis. Hyperpolarized ^129^Xe NMR (see Supporting Information Figures S15, S16), and especially the line shape analysis in the high‐temperature regime (Figure 2C) reveal that the adsorbed signal consists of two different resonances with a markedly different depolarization behavior. The intensity of the highfield signal increases with decreasing temperature, which is expected for diamagnetic systems due to the larger Xe uptake.^[15]^ In contrast, the intensity of the downfield signal decreases, demonstrating that depolarization dominates as typical for graphite.^[16]^ We thus assign the two ^129^Xe resonances to domains consisting of graphite and a diamagnetic component (e. g., a carbon nitride like region). In the low loading limit at high temperatures, usually smaller pores are preferentially populated. The depolarization behavior of the adsorbed hyperpolarized ^129^Xe (Figure 2C) thus suggests that the smaller pores occur predominantly in the graphitic regions. Considering the bimodal pore size distribution with nearly the same pore volume for both pore size ranges (see Supporting Information Figure S5 and Figure 1C [catalyst]), it is suggestive that the region with the larger pores contains the observed NH_x_ functionalities. A fit of the PXRD data of the N‐SiC support material after NaOH treatment (blue) resulted in a crystalline phase fraction of about 17 % (total fit yellow, Figure 2D and Supporting Information Figure S9) on a big amorphous background (green). A widened average interlayer distance of 3.57 Å was found by fitting the (002) reflex in PXRD data, alongside a smaller fraction of stronger expanded stacks with an average interlayer distance of 4.52 Å in comparison to hexagonal graphite with an interlayer distance of 3.354 Å (see the Supporting Information 3.8). The multinuclear NMR and Raman spectra (see Figure 2 and Supporting Information Figure S6) are in accordance with a dominantly amorphous graphitic material. The PDF of the N‐SiC support (see the Supporting Information Figure S10) indicates hexagonal graphite structure. Below 5 Å, all distances match in‐plane carbon–carbon distances in hexagonal carbon rings. Since the PDF decayed to 0 beyond about 20 Å, it can be concluded that the structural coherence of the N‐SiC support is about 2 nm. Rather high atomic displacement parameters u_33_ along the *c*‐axis corroborate turbostratic disorder in PDF refinements. HRTEM based analysis of the graphitic domains (see the Supporting Information Figure S8) is in accordance and revealed a mean size of the graphitic domains of 1.6 nm and a widened interlayer distance of 3.6 Å. Acid‐base titration revealed the presence of 0.42 mmol accessible basic N atom functionalities per gram N‐SiC material after activation.

In summary, our N‐SiC support is an N doped (≈7 wt%) amorphous carbon material (processed at 1000 °C) having graphitic and nitrogen doped domains smaller than 2 nm with a turbostratic disorder and widened interlayer distances. The material is predominantly microporous with a bimodal size distribution, which might be chemically different. The larger one of them seems to contain the accessible basic NH functionalities most likely formed via hydrolysis of C−N−Si bonds during NaOH treatment.

## Conclusion

We report on a highly active 3d‐metal catalyst for the selective synthesis of primary amines via reductive amination employing hydrogen, a key reaction in industry and academia. Our catalyst is nanostructured and reusable, has a large scope, and outperforms noble metal catalysts. The key for the high activity seems to be our support: an amorphous N‐doped carbon material with small graphitic domains and with pores that carry NH‐functionalities. 88 % of the basic NH‐functionalities are still present or available in the final cobalt catalyst. The accessible NH‐functionalities can bind cobalt species during catalyst synthesis or even catalysis and might catalyze the crucial condensation step. Related materials might be suitable for a more rational design of highly active 3d‐metal catalysts.

## Experimental Section

### General considerations

All air‐ and moisture sensitive reactions were performed under dry argon or nitrogen atmosphere using standard Schlenk and glovebox techniques. All dried solvents were obtained from a solvent purification system (activated alumina cartridges) or purchased from Acros. Deuterated solvents were dried via molecular sieves. All chemicals were acquired from commercial sources with purity over 95 % and used without further purification. The precursor SMP‐10 was purchased from Starfire Systems, New York, USA. Pyrolysis of the support material was carried out under nitrogen atmosphere in a high temperature furnace (Gero, Berlin, Germany). Pyrolysis and reduction of the catalyst were performed under nitrogen or forming gas (90 : 10) atmosphere in a ChemBET Pulsar TPR/TPD instrument from Quantachrome. TEM was carried out by using a Variant LEO 9220 (200 kV) and a JEOL JEM 2200FS (200 kV) device. For the sample preparation, the samples were suspended in chloroform and sonicated for 5 min.

Pore characterizations were carried out via nitrogen sorption measurements using a 3P Micro 100 Surface Area and Pore Size Analyzer device. The pore size distribution was computed via DFT calculations (calculation model: Ar at −186.15 °C on cylindrical pore, MDFT equilibrium model). The specific surface area was calculated by using *p*/*p*_0_ values from 0.005–0.1 (BET).

NMR measurements were performed using a Varian INOVA 300 (300 MHz for 1H, 75 MHz for ^13^C) and a Bruker Avance III HD 500 (500 MHz for 1H, 125 MHz for ^13^C) instrument at 296 K.

The hydrogenation experiments were carried out with Parr Instrument stainless‐steel autoclavesN‐MT5 250 mL equipped with heating mantles and temperature controller.

PXRD data was collected at 65 keV (0.1907 Å) at ID31 at the European Synchrotron Radiation Facility (ESRF) with a CdTe Dectris Pilatus X 2 M detector. NIST cerium oxide standard was used for distance calibration and determination of instrumental resolution (*Q*_damp_=0.016; *Q*_broad_=0.008). Fits to the PXRD data were conducted with the multipeak fitting option within the software Igor Pro 8.

^129^Xe, ^29^Si, ^15^N and ^13^C solid‐state NMR measurements were performed on a Bruker Avance II NMR spectrometer operating at an external field of 7.05 T corresponding to Larmor frequencies of 83.4, 59.6, 30.4 and 75.5 MHz for the ^129^Xe, ^29^Si, ^15^N and ^13^C, respectively.

Variable‐temperature measurements of hyperpolarized (hp) ^129^Xe spectra were performed using a wideline 5 mm double‐resonance Bruker probe. Hyperpolarized Xenon was supplied by a homebuilt polarizer with a gas composition of 1 % *v*/*v* Xe with natural abundance of the ^129^Xe isotope, 6 % *v*/*v* N_2_ as fluorescence quenching gas and He as buffer gas at a system pressure of 4×10^5^ Pa. ^129^Xe wideline SP NMR spectra were recorded in steps of 10 K with an equilibration time of 3 min before starting the acquisition. The pulse length for the π/2 pulse was set to 3.25 μs with a nutation frequency of approximately 77 kHz. Two scans were accumulated for each temperature with a temperature dependent recycle delay between 1 s at room temperature and 120 s at 130 K. The chemical shift was referenced to gaseous xenon at zero pressure (0 ppm).

^13^C NMR spectra were recorded as SP and CP experiments under MAS with a spinning speed of 10 kHz using a Bruker 4 mm triple‐resonance MAS probe. SP MAS NMR spectra were accumulated with pulse lengths of 2.3 μs for the π/2 pulse with a nutation frequency of approximately 110 kHz. Recycle delays were varied between 10 and 300 s after a pre‐saturation pulse train.

### Catalytic procedures

All catalytic reactions were carried out after the procedure described in the following: A magnetic stirring bar, 0.5 mmol ketone, 3.5 mL aq. NH_3_ (32 %) and 2 mol% Co catalyst (3.0 wt% Co, 0.01 mmol Co 0.59 mg Co, 19.7 mg catalyst) were filled in a 5 mL glass reaction vial. For aldehydes, 1.5 mol% Co was used. The vial was placed in a 250 mL high‐pressure autoclave (Parr Instruments) and flushed three times with 0.5 MPa of hydrogen. The autoclave was pressurized with 1.0 MPa of hydrogen and the reaction was stirred for 20 h at 50 °C for ketones and 80 °C for aldehydes. After cooling to room temperature and release of the hydrogen pressure, the solution was extracted five times with methyl *tert*‐butyl ether and dried over Na_2_SO_4_. The resulting solution was filtered and the solvent was removed under reduced pressure. To obtain the amine hydrochloride salts, 0.5 mL HCl in ether were added. The solvent was removed under reduced pressure and the resulting solid was then further analyzed by NMR spectroscopy.

## Conflict of interest

The authors declare no conflict of interest.

## Supporting information

As a service to our authors and readers, this journal provides supporting information supplied by the authors. Such materials are peer reviewed and may be re‐organized for online delivery, but are not copy‐edited or typeset. Technical support issues arising from supporting information (other than missing files) should be addressed to the authors.

Supplementary
