# Supplementary material for: Co‐Catalyzed Synthesis of Primary Amines via Reductive Amination employing Hydrogen under very mild Conditions
Source: ChemSusChem. 2021 May 6;14(11):2360–6. doi: 10.1002/cssc.202100553 (PMC8251741; doi:10.1002/cssc.202100553)
Supplement: Supplementary file 1 — Supplementary [file CSSC-14-2360-s001.pdf]

ChemSusChem

Supporting Information

Co-Catalyzed Synthesis of Primary Amines via Reductive Amination employing Hydrogen under very mild Conditions

Matthias Elfinger, Timon Schönauer, Sabrina L. J. Thomä, Robert Stäglich, Markus Drechsler, Mirijam Zobel, Jürgen Senker, and Rhett Kempe*© 2021 The Authors. ChemSusChem published by Wiley-VCH GmbH. This is an open access article under the terms of the Creative Commons Attribution License, which permits use, distribution and reproduction in any medium, provided the original work is properly cited.

Table of contents

1 General considerations ... 3
2. Experimental procedures ... 5
2.1 Synthesis of the support material ... 5
2.2. Synthesis of the catalyst ... 5
2.3 Catalytic procedures ... 5
2.4 Catalytic studies ... 6
2.4.1 Screening of reaction parameters ... 6
2.4.2 Reusability ... 8
2.4.3 Upscaling ... 9
3. Characterization of the catalyst ... 9
3.1 ICP-OES ... 9
3.2 XPS ... 10
3.3 SEM/EDX ... 11
3.4 TEM ... 12
3.5 Argon physisorption ... 13
3.6 Raman spectroscopy and elemental analysis ... 14
3.7 HR-TEM graphitic domain analysis ... 15
3.8 PXRD and PDF ... 16
3.9 Solid State NMR spectroscopy ... 19
4. Characterization of the isolated products ... 23
5. NMR Spectra ... 36
6. References ... 73

1 General considerations

All air- and moisture sensitive reactions were performed under dry argon or nitrogen atmosphere using standard Schlenk and glove box techniques. All dried solvents were obtained from a solvent purification system (activated alumina cartridges) or purchased from Acros. Deuterated solvents were dried via molecular sieves. All chemicals were acquired from commercial sources with purity over 95 % and used without further purification. The precursor SMP-10 was purchased from Starfire Systems, New York, USA. Pyrolysis of the support material was carried out under nitrogen atmosphere in a high temperature furnace (Gero, Berlin, Germany). Pyrolysis and reduction of the catalyst were performed under nitrogen or forming gas (90/10) atmosphere in a ChemBET Pulsar TPR/TPD instrument from Quantachrome. Transmission electron microscopy (TEM) was carried out by using a Variant LEO 9220 (200 kV) and a JEOL JEM 2200FS (200 kV) device. For the sample preparation, the samples were suspended in chloroform and sonicated for 5 min. For analysis, LC200-Cu and CF200-Cu grids were used.

Pore characterizations were carried out via nitrogen sorption measurements using a 3P Micro 100 Surface Area and Pore Size Analyzer device. The pore size distribution was computed via DFT calculations (calculation model: Ar at -186.15 °C on cylindrical pore, MDFT equilibrium model). The specific surface area was calculated by using p/p_0 values from 0.005-0.1 (BET).

The cobalt content was determined by ICP-OES. The fusion of the catalyst was carried out in a Berghof SpeedWave 4 microwave, for the ICP-OES measurement a Varian Vista Pro was used.

X-ray photoelectron spectroscopy (XPS) was performed using a Physical Electronics Phi 5000 Versa Probe III instrument. As X-ray source a monochromatic Al K α with a spot size of 100 µm
(21.1 W) was used. The kinetic pass energy of the photoelectrons was determined with a hemispheric analyzer (45°) set to pass energy of 13 eV for high-resolution spectra.

NMR measurements were performed using a Varian INOVA 300 (300 MHz for ^{1}H, 75 MHz for ^{13}C) and a Bruker Avance III HD 500 (500 MHz for ^{1}H, 125 MHz for ^{13}C) instrument at 296 K. Chemical shifts are reported in ppm relative to the residual solvent signal (DMSO-d6: 2.50 ppm (^{1}H), 39.51 ppm (^{13}C)), coupling constants (J) are reported in Hz.

GC analyses were carried out on an Agilent 6850 GC system equipped with an Optima 17 column (30 m x 0.32 mm x 0.25 µm).

The hydrogenation experiments were carried out with Parr Instrument stainless steel autoclaves N-MT5 250 mL equipped with heating mantles and temperature controller.

Powder X-ray diffraction (PXRD) data was collected at 65 keV (0.1907 Å) at ID31 at the European Synchrotron Radiation Facility (ESRF) with a CdTe Dectris Pilatus X 2M detector. All samples were measured in 1 mm Kapton® capillaries. NIST cerium oxide standard was used for distance calibration and determination of instrumental resolution (Q_{damp} = 0.016; Q_{broad} = 0.008). Masking and distance calibration were done with the software package pyFAI[1]. Azimuthal integration to powder diffraction patterns was performed with xpdtools[2]. Pair Distribution Function (PDF) calculation was done with PDFgetX3[3], using a Q-range of at least 0.9 – 18.0 Å^{-1}. For the support material, an elemental composition of $SiC_{9.36}N_{0.99}O_{1.62}$ derived from elemental analysis (see 3.6) was used while a nominal composition of CoNSiC was employed for the calculation of the difference-PDF of the catalyst using the unloaded support as background. The I(Q) of the support material was rescaled for analysis of the materials in reciprocal space as well as for calculation of the difference-PDF, so that reflexes of Co-containing phases are positive in the difference signal. For PDF modelling we used PDFgui[4]. Cif files for PDF modelling were downloaded from the American Mineralogist Crystal Structure Database (AMCSD)[5] or created from published structures[6] found with the software X'Pert Highscore. Fits to the PXRD data were conducted with the multipeak fitting option within the software Igor Pro 8.

^{129}Xe, ^{29}Si, ^{15}N and ^{13}C solid state NMR measurements were performed on a Bruker Avance II NMR spectrometer operating at an external field of 7.05 T corresponding to Larmor frequencies of 83.4, 59.6, 30.4 and 75.5 MHz for the ^{129}Xe, ^{29}Si, ^{15}N and ^{13}C, respectively.

Variable temperature measurements of hyperpolarized (hp) ^{129}Xe spectra were performed using a wideline 5mm double-resonance Bruker probe. Hyperpolarized Xenon was supplied by a homebuilt polarizer with a gas composition of 1 % v/v Xenon with natural abundance of the ^{129}Xe isotope, 6 % v/v N_2 as fluorescence quenching gas and Helium as buffer gas at a system pressure of $4 \cdot 10^5$ Pa. ^{129}Xe wideline single pulse (SP) NMR spectra were recorded in steps of 10 K with an equilibration time of 3 minutes before starting the acquisition. The pulse length for the π/2 pulse was set to 3.25 µs with a nutation frequency of approximately 77 kHz. Two scans were accumulated for each temperature with a temperature dependent recycle delay between 1 s at room temperature and 120 s at 130 K. The chemical shift was referenced to gaseous xenon at zero pressure (0 ppm).

^{13}C NMR spectra were recorded as SP and cross polarization (CP) experiments under magic angle spinning (MAS) with a spinning speed of 10 kHz using a Bruker 4 mm triple-resonance MAS probe. SP MAS NMR spectra were accumulated with pulse lengths of 2.3 µs for the π/2 pulse with a nutation frequency of approximately 110 kHz. Recycle delays were varied between 10 and 300 s after a pre-saturation pulse train. For the CP MAS NMR spectroscopic measurements an initial π/2 pulse of 2.5 µs with a nutation frequency of 100 kHz for protons was utilized, followed by 5 ms CP contact time with a nutation frequency of 65 kHz on the ^{13}C channel and a linearly ramped pulse from 50 kHz to 100 kHz on the proton channel.[13] Heteronuclear broadband decoupling was performed with a SPINAL-64 sequence[14] using a ^{1}H nutation frequency of 70 kHz with phase increments of 5 degrees. Between 64 to 256 scans were performed for SP MAS NMR spectroscopic measurements and between 3000 to 4000 scans were accumulated for CP MAS NMR spectra. The ^{13}C MAS NMR spectra are referenced indirectly with respect to tetramethylsilane (TMS) using adamantane as secondary reference.

^{29}Si SP NMR spectra were recorded under MAS with a spinning speed of 10 kHz. 2600 (before activation) / 4600 (after activation) scans were accumulated with a recycle delay of 60 s and a pulse length of 3.2 µs for the initial π/2 pulse.

For the ^{15}N CP MAS experiments a 4 mm triple-resonance MAS probe was utilized with a spinning speed of 5 kHz. An initial pulse length for protons of 4.8 µs with a nutation frequency of 52 kHz was used, followed by 500 µs / 1000 µs CP contact time.[13] SPINAL-

64 sequence was utilized for heteronuclear decoupling with a frequency of 55 kHz.[14] with a recycle delay of 1 s a total of 530,000 / 300,000 scans were accumulated.
Solid-state NMR spectroscopic studies of ^{1}H nuclei were performed on a Bruker Avance III HD NMR spectrometer at an external field of 14.1 T, corresponding to a larmor frequency of 600.1 MHz. The measurements were carried out in a 1.3 mm MAS double-resonance wide bore probe with commercial ZrO_2 rotors with a spinning speed of 62.5 kHz. For the acquisition of ^{1}H SP NMR spectra, a nutation frequency of 180 kHz with a pulse length of 1.4 µs, 32 scans and a recycle delay of 3 s were set. The ^{1}H-^{14}N *D*-HMQC[15,16] experiment was performed with a spinning of 50.0 kHz ($v_0(^{14}N)$ = 43.4 MHz). The super-cycled symmetry based $SR4_1^2$ sequence[17] was used for the ^{1}H-^{14}N dipolar recoupling. The recoupling time was adjusted to 240 µs. A rotor-synchronized delay was employed between the two ^{14}N pulses. The ^{14}N pulses were set to 28 µs using a B_1 field of approximately 60 kHz.

2. Experimental procedures

2.1 Synthesis of the support material

For the preparation of the N-SiC support material a known literature method was modified.[7]
200 mg SMP-10 (StarPCS^{TM}), 0.987 mL (0.800 g, 15.08 mmol) acrylonitrile and 45 mg (0.28 mmol) azobis(isobutyronitrile) were dissolved in 4 mL dimethylformamide and crosslinked for 16 h at 75 °C. After solvent removal under reduced pressure, the obtained greenbody was pyrolyzed using the following program:

$$20\ °\mathrm{C} \xrightarrow{5\ \mathrm{K/min}} 200\ °\mathrm{C}\ (0\ \mathrm{h}) \xrightarrow{1\ \mathrm{K/min}} 400\ °\mathrm{C}\ (0\ \mathrm{h}) \xrightarrow{5\ \mathrm{K/min}} 1000\ °\mathrm{C}\ (1\ \mathrm{h}) \xrightarrow{10\ \mathrm{K/min}} 20\ °\mathrm{C}$$

After ball mining for 40 minutes, 400 mg of the ceramic were washed by stirring in solution of 5.3 mL aq. NaOH (1 M) and 4 mL MeOH at 85 °C for 24 h. Afterwards the material was washed until neutrality and dried at room temperature.

2.2. Synthesis of the catalyst

1000 mg N-SiC were impregnated with 148 mg $Co(NO_3)_2 \cdot 6\ H_2O$ in 20 mL H_2O and stirred at 110 °C until the solvent was completely removed. Afterwards, the material was pyrolyzed and reduced using the following program:

$$\mathrm{RT} \xrightarrow[N_2]{2\ \mathrm{K/min}} 300\ °\mathrm{C} \xrightarrow[N_2]{10\ \mathrm{K/min}} 700\ °\mathrm{C}\ (0.5\ \mathrm{h}) \xrightarrow[N_2]{20\ \mathrm{K/min}} 100\ °\mathrm{C} \xrightarrow[N_2/H_2]{5\ \mathrm{K/min}} 550\ °\mathrm{C}\ (3\ \mathrm{h}) \xrightarrow[N_2/H_2]{20\ \mathrm{K/min}} 40\ °\mathrm{C}$$

2.3 Catalytic procedures

All catalytic reactions were carried out after the procedure described in the following: A magnetic stirring bar, 0.5 mmol ketone, 3.5 mL aq. NH_3 (32%) and 2 mol % Co catalyst (3.0 wt% Co, 0.01 mmol Co 0.59 mg Co, 19.7 mg catalyst) were filled in a 5 mL glass reaction vial. For aldehydes, 1.5 mol% Co were used. The vial was placed in a 250 mL high pressure autoclave (Parr Instruments) and flushed three times with 0.5 MPa of hydrogen. The autoclave was pressurized with 1.0 MPa of hydrogen and the reaction was stirred for 20 h at 50 °C for ketones and 80 °C for aldehydes. After cooling to room temperature and release of the hydrogen pressure, the solution was extracted five times with methyl *tert*-butyl ether and dried over Na_2SO_4. The resulting solution was filtered and the solvent was removed under reduced pressure. To obtain the amine hydrochloride salts, 0.5 mL HCl in ether were added. The solvent was removed under reduced pressure and the resulting solid was then further analyzed by NMR spectroscopy.

2.4 Catalytic studies

2.4.1 Screening of reaction parameters

Solvent screening of the model substrate acetophenone

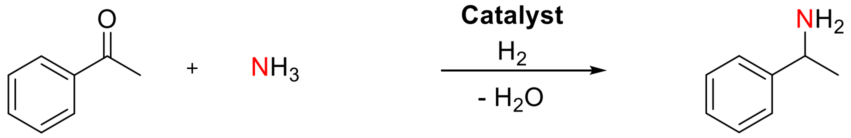

**Solvent**	**Yield / %**
NH_3 in Dioxane (0.5 M)	0
NH_3 in EtOH (0.5 M)	0
NH_3 in H_2O (0.5 M)	4
NH_3 in EtOH (2.0 M)	0
NH_3 in H_2O (13.4 M)	16
NH_3 in H_2O (17.2 M)	30

Reaction conditions: 1.5 mol% Co (2.24 wt% Co, 0.0075 mmol Co, 0.44 mg Co), 0.5 mmol acetophenone; 30 °C, 20 h, 1.0 MPa H_2, 3.0 mL solvent; yields were determined by GC using *n*-dodecane as an internal standard.

Screening of the NH_3 amount

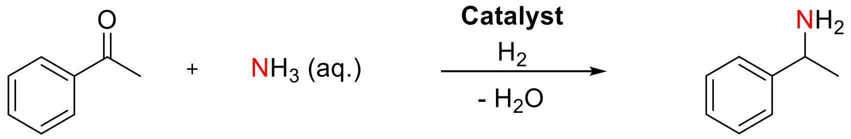

**Volume aq. NH_3 (32 %) / mL**	**Yield / %**
1	0
2	0
2.5	14
3	30
3.5	40
4	8
5	4
6	2

Reaction conditions: 1.5 mol% Co (2.24 wt% Co, 0.0075 mmol Co, 0.44 mg Co), 0.5 mmol acetophenone; 30 °C, 20 h, 1.0 MPa H_2, solvent: aq. NH_3 (32 %); yields were determined by GC using *n*-dodecane as an internal standard.

Screening of the metal loading of the catalyst

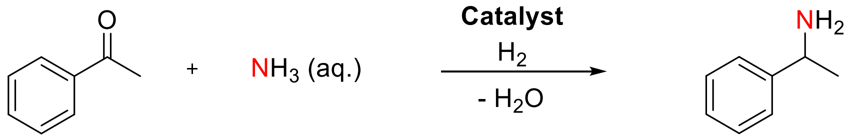

**Theoretical wt% of Co**	Yield / %
2	5
3	35
4	32
5	22

Reaction conditions: 1.5 mol% Co (2.24 wt% Co, 0.0075 mmol Co, 0.44 mg Co), 0.5 mmol acetophenone; 30 °C, 20 h, 1.0 MPa H_2, 3.5 mL aq. NH_3 (32 %); yields were determined by GC using *n*-dodecane as an internal standard.

Screening of the catalyst loading

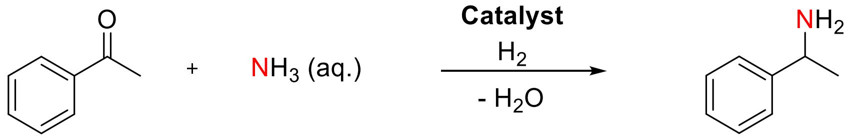

**Catalyst loading / mol%**	**Yield / %**
1	8
2	38
3	46
4	73
5	76

Reaction conditions: 1.5 mol% Co (2.24 wt% Co, 0.0075 mmol Co, 0.44 mg Co), 0.5 mmol acetophenone; 30 °C, 20 h, 1.0 MPa H_2, 3.5 mL aq. NH_3 (32 %); yields were determined by GC using *n*-dodecane as an internal standard.

Screening of the reaction temperature

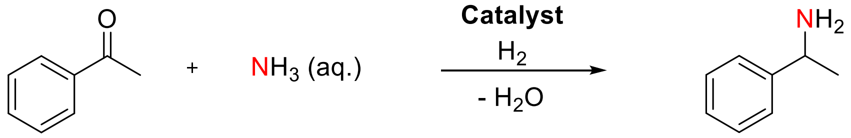

**Temperature / °C**	**Yield / %**
30	32
40	42
50	99
60	99

Reaction conditions: 1.5 mol% Co (2.24 wt% Co, 0.0075 mmol Co, 0.44 mg Co), 0.5 mmol acetophenone; 20 h, 1.0 MPa H_2, 3.5 mL aq. NH_3 (32 %); yields were determined by GC using *n*-dodecane as an internal standard.

Temperature screening of the model substrate benzaldehyde

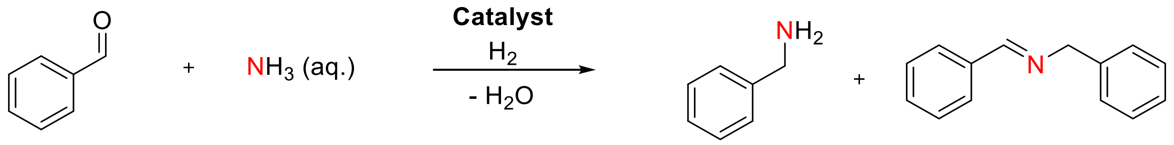

1 **2**

**Temperature / °C**	Yield 1 / %	Yield 2 / %
40	0	73
50	0	77
60	12	64
70	34	46
80	83	7

Reaction conditions: 1.1 mol% Co (2.24 wt% Co, 0.0055 mmol Co 0.32 mg Co), 0.5 mmol benzaldehyde; 20 h, 1.0 MPa H_2, 3.5 mL aq. NH_3 (32 %); yields were determined by GC using *n*-dodecane as an internal standard.

2.4.2 Reusability

The reusability of the catalyst was investigated using the reductive amination of acetophenone with aqueous ammonia. The reaction was carried out for 13 h under the optimized reaction conditions to obtain 60 % yield of 1-phenylethylamine, which was determined by GC using *n*-dodecane as an internal standard. After each run, the reaction mixture was separated from the catalyst using Pasteur pipettes. This was possible due to the magnetic catalyst, which was attracted to the magnetic stirring bar inside the reaction vial. The catalyst was washed two times with water and used directly for the subsequent run. The procedure was carried out 5 times without a loss of activity (Fig. S1).

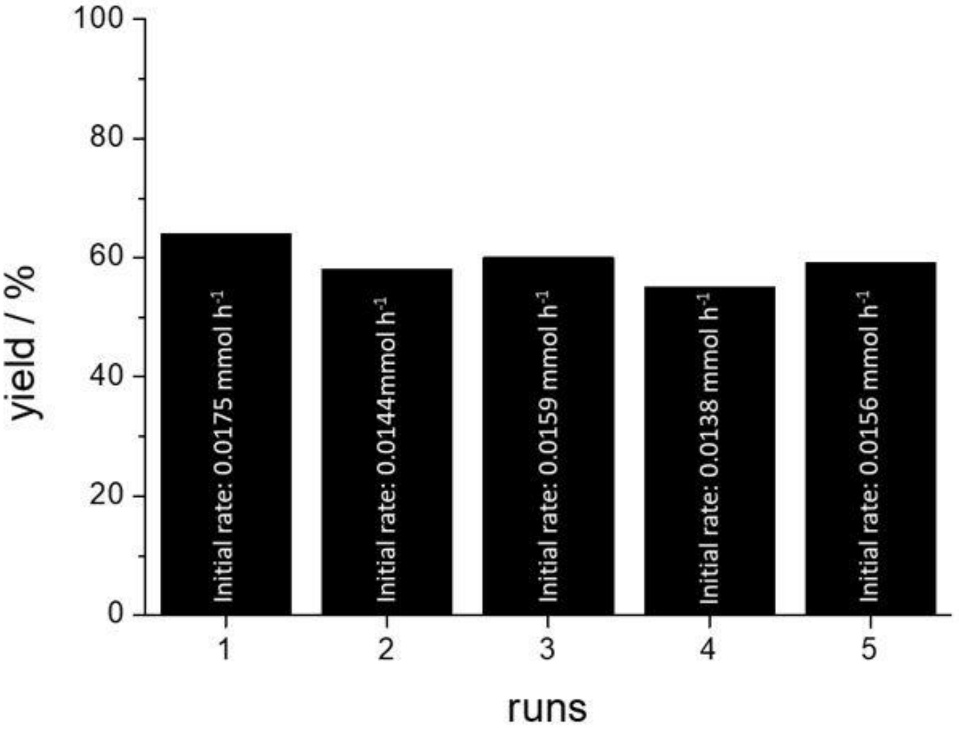

Figure S1: Reusability and initial rates of the Co/N-SiC catalyst for 5 consecutive runs. Reaction conditions for 60 % yield: 1.5 mol% Co (2.24 wt% Co, 0.0075 mmol Co, 0.44 mg Co), 0.5 mmol acetophenone; 13 h, 1.0 MPa H_2, 3.5 mL aq. NH_3 (32 %); yields were determined by GC using *n*-dodecane as an internal standard.

2.4.3 Upscaling

Acetophenone was used as a model substrate to carry out an upscaling experiment. For the reaction, 392 mg Co/N-SiC (3 wt%, 2.0 mol%), 10 mmol acetophenone and 70 mL aq. NH_3-32 % were stirred in a Schott bottle within a 250 mL high pressure autoclave (Parr Instruments) under the optimized reaction conditions (50 °C, 1.0 MPa H_2, 20 h). The workup was identical to the 0.5 mmol reactions. Isolated yield of 1-phenylethanaminium chloride: 1.495 g, 95 %.

3. Characterization of the catalyst

3.1 ICP-OES

25 mg of the sample were solved in 1.5 mL HNO_3 (65 %, distilled), 4.5 mL HCl (32 %, p.a.) and 1 mL HF (40 %) and heated in the microwave at 170 °C for 7 min (80 % power), at 180 °C for 7 min (85 % power) and 195 °C for 20 min (90 % power).

Theoretical Co content: 3 wt%
Measured Co content: 2.24 wt%

3.2 XPS

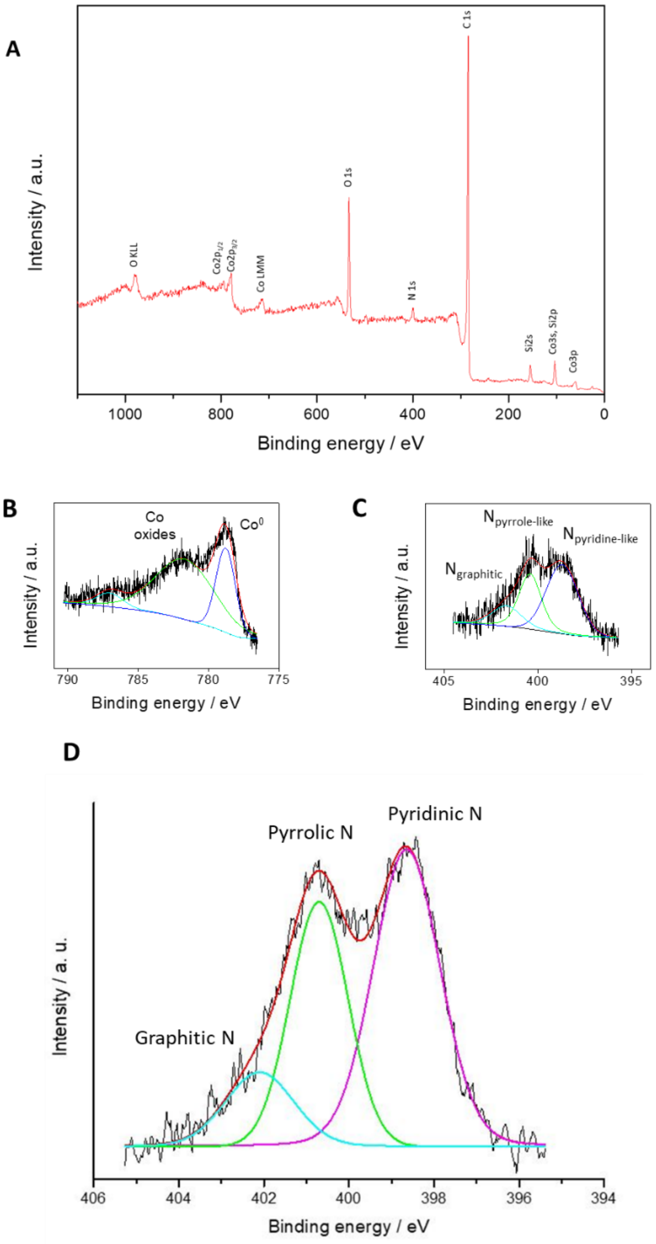

Figure S2: X-ray photoelectron spectra (XPS) of the catalyst. **A**: overview spectrum. **B**: analysis of the Co $2p_{3/2}$ area shows the presence of metallic cobalt (28 %) and cobalt oxide (65 %). **C**: analysis of the nitrogen area suggests different binding modes of nitrogen within the catalyst (graphitc: 16 %, pyrrole-like: 33 %, pyridine-like: 51 %). **D:** High resolution XPS of the N1s region of the catalyst.

The N_{1s} region of the XPS-spectrum (Fig. S2**D**) shows three major peaks, which can be addressed to 16.4 % graphitic nitrogen (401.3 eV), 32.8 % pyrrolic nitrogen (400.5 eV) and 50.8 % pyridinic nitrogen (398.6 eV). The Co and CoO ratios determined by fitting the Co_{2p} region (28 % Co; 65 % CoO) are consistent with those obtained by PDF (23 % Co; 77 % CoO; see 3.8).

3.3 SEM/EDX

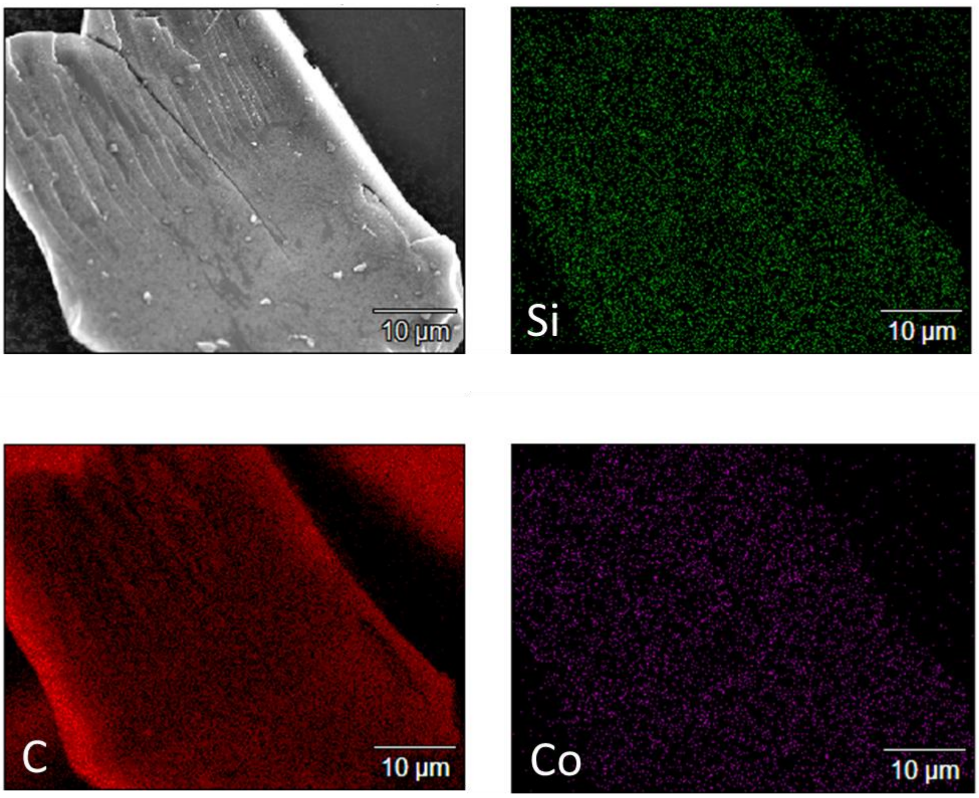

Figure S3: Scanning electron microscopy (SEM) in combination with energy dispersive X-ray (EDX) mapping. The measurements show a homogeneous distribution of all individual elements, including cobalt, on the surface of the support material.

3.4 TEM

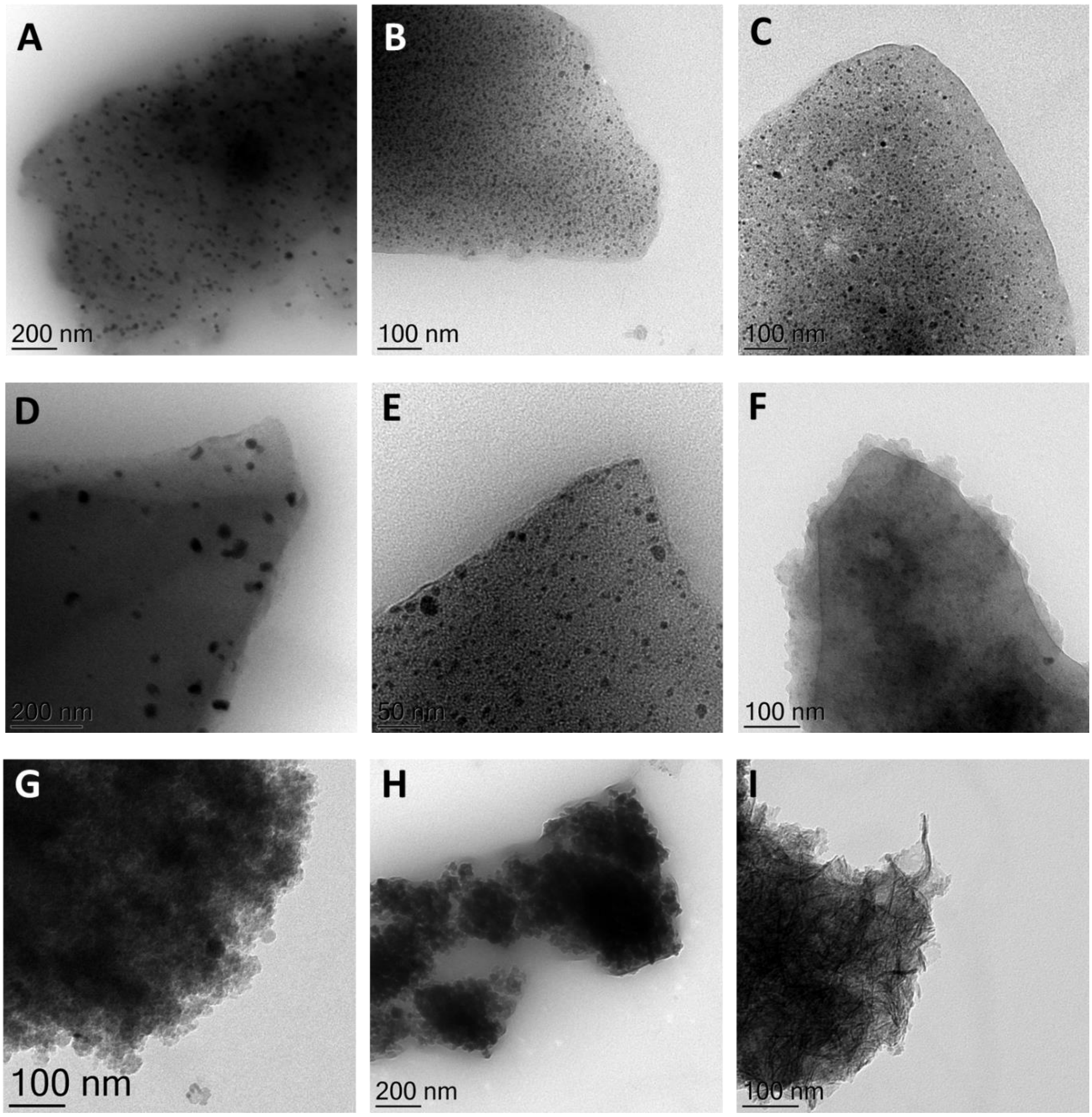

Figure S4: TEM characterization of the catalyst used for the activity screening in the reductive amination of ketones. Variation of the metal content regarding the Co/N-SiC catalyst: A) 2 wt% Co; B) 4 wt% Co; C) 5 wt% Co; Variation of the pyrolysis temperature of the Co/N-SiC catalyst: D) 600 °C; E) 800 °C; Variation of the support material while using $Co(NO_3)_2 \cdot 6\ H_2O$ as metal source: F) Co/activated carbon; G) Co/SiO_2; H) Co/TiO_2; I) Co/Al_2O_3

3.5 Argon physisorption

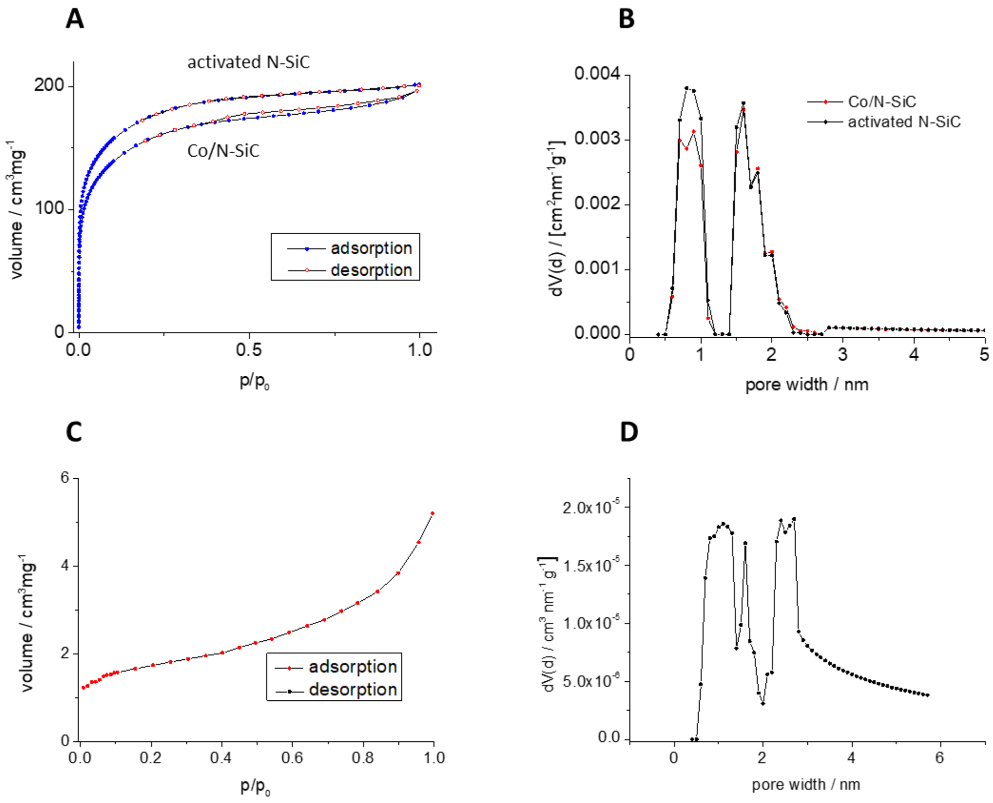

Figure S5: A Surface characterization and **B** pore size distribution of the catalyst and activated N-SiC support via argon physisorption measurements [calculation model: Ar at 87 K on carbon (cylindr. pores, NLDFT equilibrium model)]. The specific surface showed a slight decline from 545 m^2g^{-1} of the activated support material to 491 m^2g^{-1} of the catalyst. **C** surface characterization and pore size distribution **D** of the support material prior to activation. The surface area increases from 6 to 545 m^2g^{-1} after treatment with NaOH solution.

3.6 Raman spectroscopy and elemental analysis

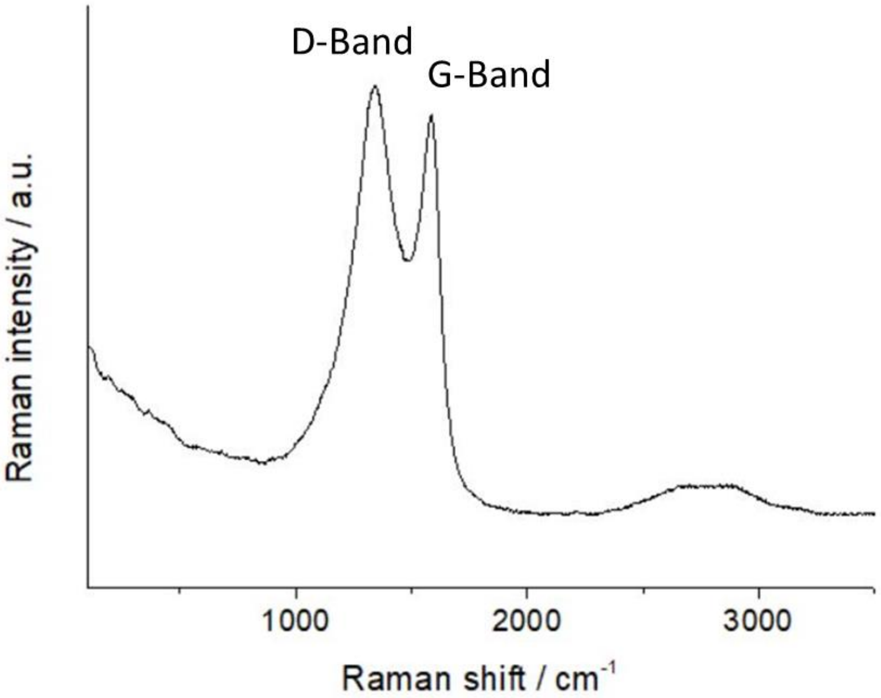

Figure S6: Raman spectrum of the activated support material

The Raman spectrum (Fig. S6) shows the D- and G-peaks, which are the most dominant. The D-peak is caused by disordered structure of graphene, such as structural defects that break the symmetry. The G-peak corresponds to the only allowed Raman transition in the spectrum region of large graphite crystals. It results from a C-C stretching in the longitudinal symmetry axis of the graphite plane.
The presence of NH_x groups in the support material could be verified by titration experiments. The calculation of the addressable amine groups in the liquid phase was done by determining the absolute number of amine groups that could be converted to ammonium chloride functionalities by the uptake of hydrogen chloride. Per 100 mg of support material, 0.04175 mmol HCl were used up. Hence, the presence of $2.514 \cdot 10^{20}$ NH_x groups could be determined. With the elemental analysis of the support material (Fig. S7), the percentage of NH_x groups compared to the whole nitrogen content can be specified.

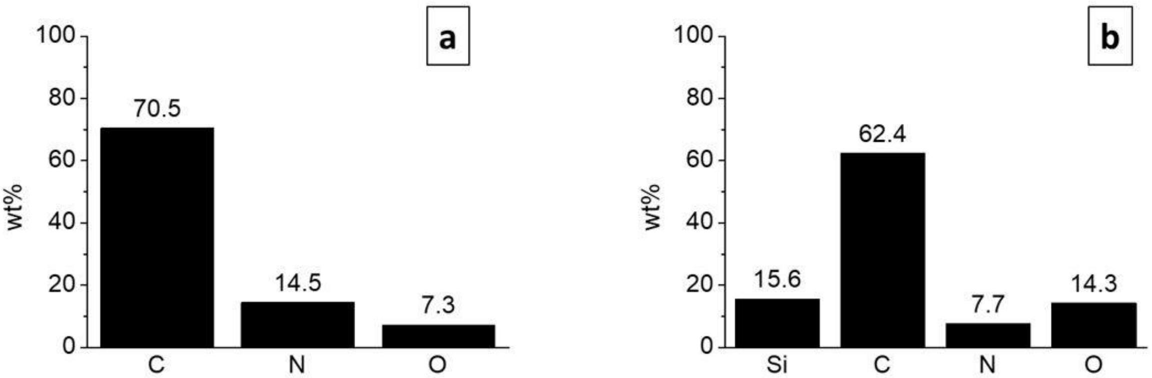

Figure S7: a) Elemental analysis of pure pyrolyzed polyacrylnitrile and **b)** activated N-SiC support material. The silicon content of N-SiC was calculated based on the difference of the measured elements to 100 %.

Based on a total nitrogen content of 7.7 wt% and 0.585 wt% NH_x groups in the support material, an amount of 7,6 % of the total nitrogen content is addressable via titration. Considering that XPS measurements (Fig. S2) are suggesting that 49.2 % of nitrogen within the N-SiC material can possibly be described as NH_x groups (graphitic and pyrrolic N), the accessible amount of NH_x groups in liquid phase seems to be significantly lower. Titration of the Co/N-SiC catalyst revealed an uptake of 0.03685 mmol HCl per 100 g, resulting in

$2.219 \cdot 10^{20}$ NHx groups. For the same experiment with used catalyst, 100 mg of it was washed with water two times and dried under vacuum conditions. The titrated amount of NH_x groups didn't show any significant changes in comparison to the unused catalyst.

3.7 HR-TEM graphitic domain analysis

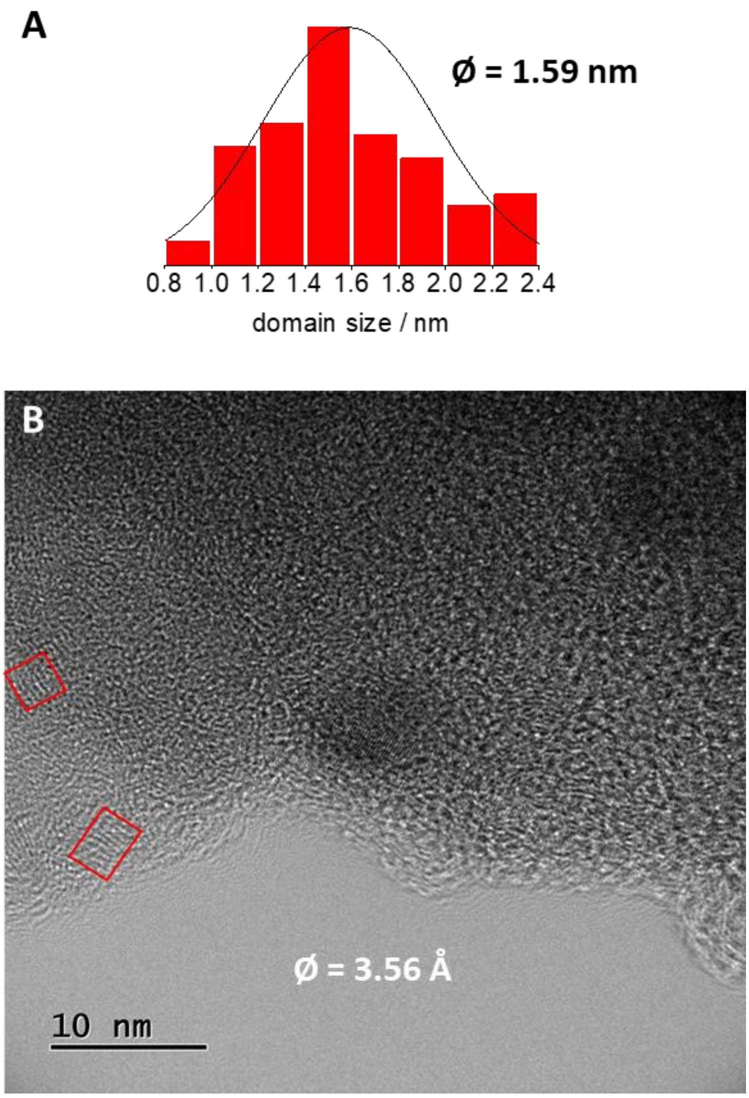

Figure S8: Size analysis of the graphitic domains within the N-SiC support via HR-TEM with a median domain size of 1.59 nm (**A**) and a median interlayer distance of 3.56 Å. Some domains show even further interlayer spacing of up to 4.2 Å (**B**, red markers). These results are in good agreement with PXRD analysis (see 3.8).

3.8 PXRD and PDF

Figure S9 panel A shows the PXRD data of both the N-SiC support and the Co/N-SiC catalyst, together with the respective difference signal catalyst minus support. Evidently the N-SiC support features broad Bragg peaks, which can be indexed by hexagonal graphite. The data shown is already corrected for background scattering of air and empty capillary, and hence the Bragg peaks sit on top of a noticeable amorphous background. To quantify the ratio of amorphous and crystalline phases in the support material we fit a continuous function (polynomial 5th order in log(Q) describes the background best) to the background in the Q-range of 0.9 - 20 Å^{-1}, as well as several Gaussian peaks to the broad Bragg peaks of the hexagonal graphite. The ratio of the integrated areas beneath the individually fitted Gaussians and the background represents the fraction of crystalline graphitic material in the sample. This results in a crystalline phase fraction of 17 % (see Figure 2D).The Bragg peaks in the pink difference curve can be assigned to two different phases based on different widths of reflexes, which were identified to be Co fcc (amcsd code 0011143)[5] and CoO fcc[6]. Additionally, the difference signal features a broad negative feature between 1.6 – 2.2 Å^{-1}, indicating that structural signal is lost in the Co/N-SiC catalyst in comparison to the N-SiC support and that the support restructured due to the Co loading.

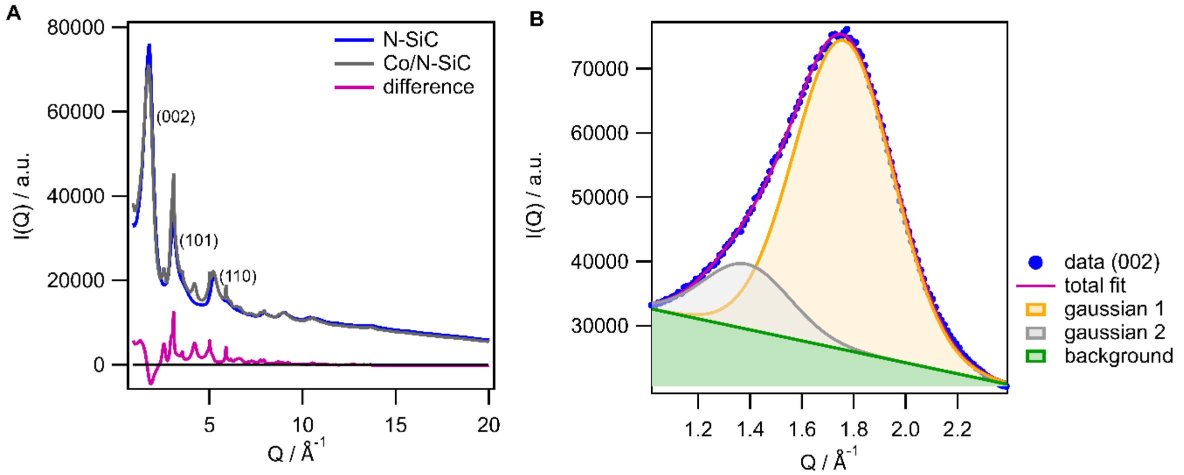

Figure S9: PXRD characterization of the catalyst and support material. A) PXRD data of N-SiC support material (blue), the Co/N-SiC catalyst (grey) and their difference (pink), which is the active catalytic phase. B) The (002) reflex (blue circles) of the N-SiC support material was fitted with two Gaussian peaks (grey and yellow; total fit pink) on a linear baseline (i.e. the amorphous support, green).

To further characterize the support material we fitted the (002) reflex with 2 Gaussians $G(x) = A \cdot e^{-\frac{(x-\mu)^2}{w^2}}$ on top of a linear baseline in the Q-range of 1.0 – 2.4 Å^{-1}, see Figure S9 B and fit results in Table S1: the peak maximum (location μ) corresponds to d-spacing of graphitic domains via d = 2π/Q, the width (w) reflects the distribution of lattice spacings around the respective average, the amplitude (A) describes the height of the peak and defines the peak area representing the corresponding phase fraction. Chi square is the goodness of fit parameter. The data was best described by two Gaussian peaks centered at 1.76 and 1.39 Å^{-1} corresponding to d-spacings of ca. 3.6 and 4.5 Å. This shows, that the majority of the crystalline graphitic domains are in average expanded by ca. 0.25 Å compared to hexagonal graphite (d-spacing of 3.354 Å). This expansion is well explained by the N-doping of graphite, increasing d-spacings. Moreover, 15 % of the graphitic domains are expanded by 1.15 Å compared to hexagonal graphite (derived from areas under peaks, i.e. 4,240/27,600). These results are in good agreement with HR-TEM analysis (see 3.7).

Table S1: Fit parameters of two Gaussian peaks to (002) reflex of PXRD of N-SiC support.

		Location (Å^{-1})	d-spacing (Å)	Amplitude	Width	Area	Total Area	Chi Square
N-SiC	Peak 0	1.39 ± 1.23e-2	4.52	1.01e+4 ± 7.14e+2	2.37e-1 ± 1.36e-2	4.24e+3 ± 5.31e+2	2.76e+4 ± 6.81e+2	1.79e+7
	Peak 1	1.76 ± 3.53e-3	3.57	4.81e+4 ± 3.95e+2	2.73e-1 ± 2.91e-3	2.33e+4 ±4.26e+2		

In order to characterize the local structure of the N-SiC support and the active catalytic species, pair distribution function (PDF) analysis was conducted. The PDF is a histogram of all interatomic distances within the sample. Figure S10 shows the PDF of the N-SiC support and its fit with the hexagonal graphite structure, Table S2 the resulting parameters. Distances < 5 Å are colour-coded in the PDF and the inset of the hexagonal ring structure of graphite: 1.42, 2.43 and 2.88 Å are nearest-neighbor distances within one ring, and 3.73, 4.24 and 4.97 Å are distances to carbon atoms in adjacent rings.

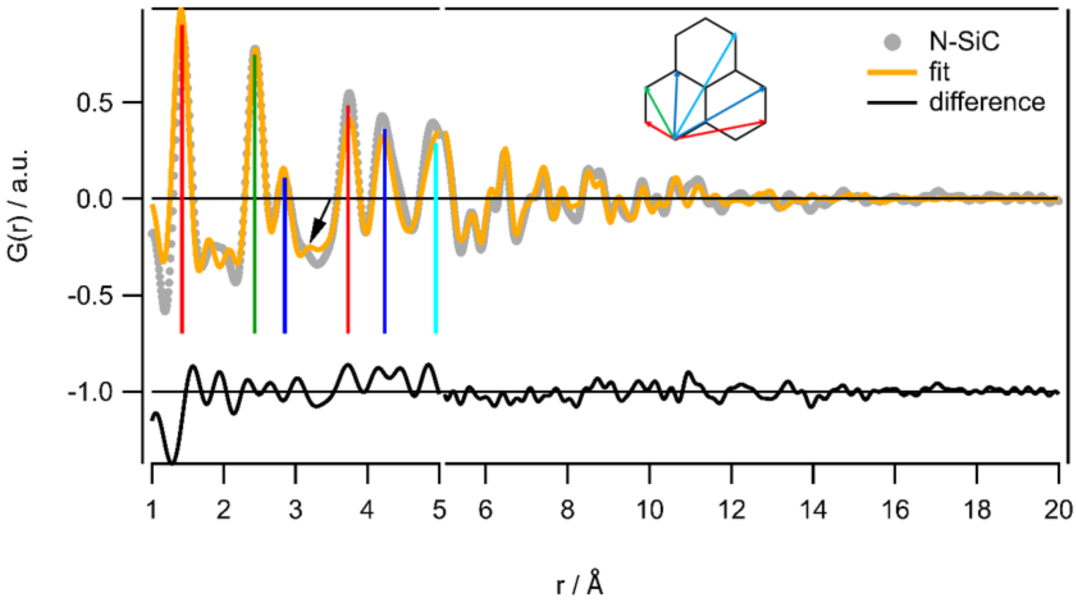

Figure S10: PDF of N-SiC (grey) with its fit (orange, graphite structure) and the resulting difference curve (black, in offset). Nearest-neighbor correlations up to 5 Å are marked in different colours and are assigned in the hexagonal ring structure in the inset. The x-axis is expanded in the region up to 5 Å. The arrow is marking a shoulder at 3.2 Å.

All peaks are fairly symmetric indicating the absence of a large portion of non-hexagonal rings.[8] Yet, the small shoulder at about 3.2 Å (see arrow Figure S10) could point towards the presence of seven-membered rings.[8a, 9]No distance correlation at the interlayer spacing of 3.6 Å is observed, suggesting turbostratic disorder[8].The discrepancies in experimental and fitted intensities of PDF peaks can be reasoned by N-doping, which reflects a 14 % increase in electron density compared to C.
The fit with the graphite structure (amcsd code 0011247)[5] was first carried out over 1-8 Å corresponding to one stack height, to retrieve atomic displacement parameters u_{11}=u_{22}, lattice parameter c and delta2. These parameters were then fixed for refining of the longer range, in order to prevent incorrect modeling of peak broadening according to Poulain et al.[8a]. For all fit ranges, the atomic displacement parameter u_{33} along the c axis was found to be quite high, corroborating the turbostratic disorder. Domain sizes, i.e. structural coherence, was refined to 19 – 23 Å depending on the fit range. Further, it is noteworthy that the lattice parameter a is shortened for the refinement for the whole range up to 20 Å as well as when only modeling the long range of 10 – 20 Å, in comparison to the short range of only up to 8 Å. Consequently, the atoms are systematically closer together in this range than predicted by the model. This indicates curvature of the sheets and implies, that structural coherence is also quite limited in ab-plane and not only along the c-axis.[8a, 10] Moreover, the fit overestimates the experimental data in the first three peaks, which might be a hint for the presence of non-hexagonal rings[11] or might stem from the N-doping. Additionally, we want to note, that the support material also contains a large fraction of amorphous and only very short-range ordered material, as shown with our analysis in reciprocal space (see Figure 2C). The amorphous fraction is not implemented in this model and thus deviations between model and data are expected.

Table S2: Results of PDF refinement of graphite to N-SiC PDF data. a, b, c: unit cell parameter (starting values in brackets); scale factor to match theoretical and experimental PDF data in intensity; u_{11}, u_{22}, u_{33}: isotropic atomic displacement parameters, delta2: parameter to account for correlated atomic motion; particle size and R_w: goodness-of-fit. Parameters, which were fixed during refinement are marked with *.

	Range 1-8	Range 1-15	Range 1-20	Range 10-20
a,b	2.4493 (2.546)	2.4477 (2.546)	2.4478 (2.546)	2.4324 (2.546)
c	6.9221 (7.140)	6.9221 (7.140)*	6.9221 (7.140)*	6.9221 (7.140)*
scale factor	0.1251	0.1327	0.1336	0.1128
u_{11}, u_{22}	0.0044	0.0044*	0.0044*	0.0044*
u_{33}	0.1906	0.2345	0.2319	0.8010
δ2	1.0536	1.0536*	1.0536*	1.0536*
particle size	23.2592	18.9321	18.5636	20.3012
**R_w**	**0.31**	**0.34**	**0.34**	**0.68**

The difference-PDF (d-PDF) of the Co/N-SiC catalyst, with the N-SiC support already subtracted, was refined with two phases being Co fcc[5] and CoO fcc phase[6], see Table S3 for refined parameters and Figure S11 for the plot..

Table S3: Results from biphasic simultaneous refinement of Co fcc and CoO fcc phases to difference-PDF data of Co/N-SiC.

Co fcc		CoO fcc	
a, b, c	3.5397 (3.548)	a, b, c	4.2319 (4.24)
scale factor	0.0601	scale factor	0.1367
particle size	92.7809	particle size	27.7549
u_{11}, u_{22}, u_{33}	0.0071	Co u_{11}, u_{22}, u_{33}	0.0126
		O u_{11}, u_{22}, u_{33}	0.060
delta 2	5.0759	delta 2	3.9211
**R_w**	**0.43**		

The biphasic refinement results in phase fractions of 77% CoO and 23% percent Co with respect to mass and of 86% CoO and 14% Co with respect to atoms and is in agreement with XPS results (see 3.2). The goodness of fit parameter R_w of 0.43 is in line with remaining structural signal up to about 8 Å in the difference curve. We hypothesize, that this signal stems from restructured support material. The first peak in this difference at 1.61 Å is not accounted for in the biphasic model. This peak matches well the bond lengths of Si-O as determined with total scattering by Tucker et al.[12]. We conjecture, that the small remaining Si phase after activation of the support forms SiO_2 domains due to heating steps carried out during Co impregnation to build the catalyst from the support.

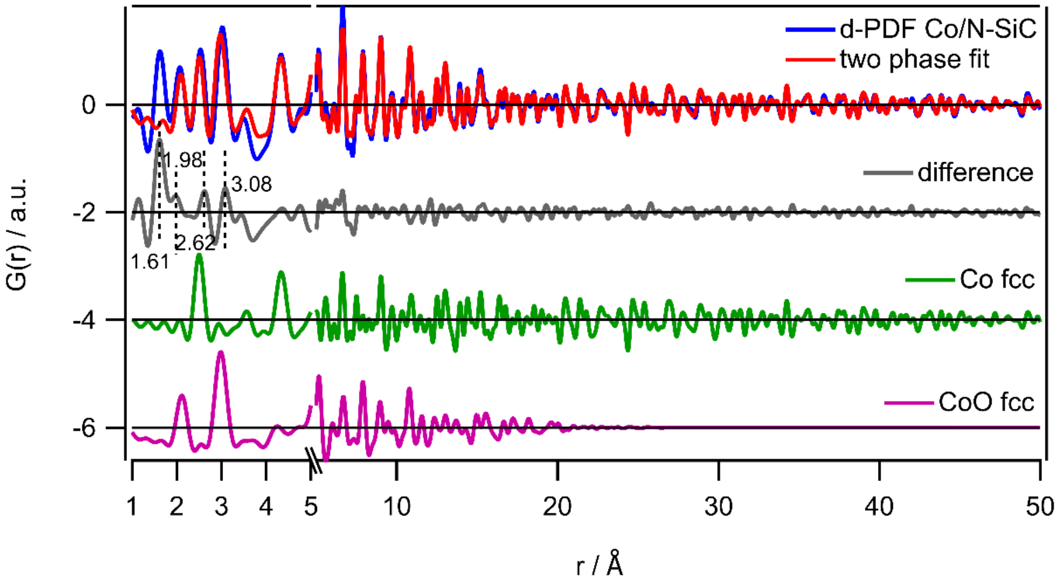

Figure S11: d-PDF of Co/N-SiC catalyst (blue) with its two phase fit (red), the resulting difference curve (grey; in offset), the contributions of Co fcc phase (green) and CoO fcc phase (purple) to the total fit (both in offset). Note that x axis is splitted and the area up to 5 Å is expanded. Distances in the difference curve in this area are labeled.

3.9 Solid State NMR spectroscopy

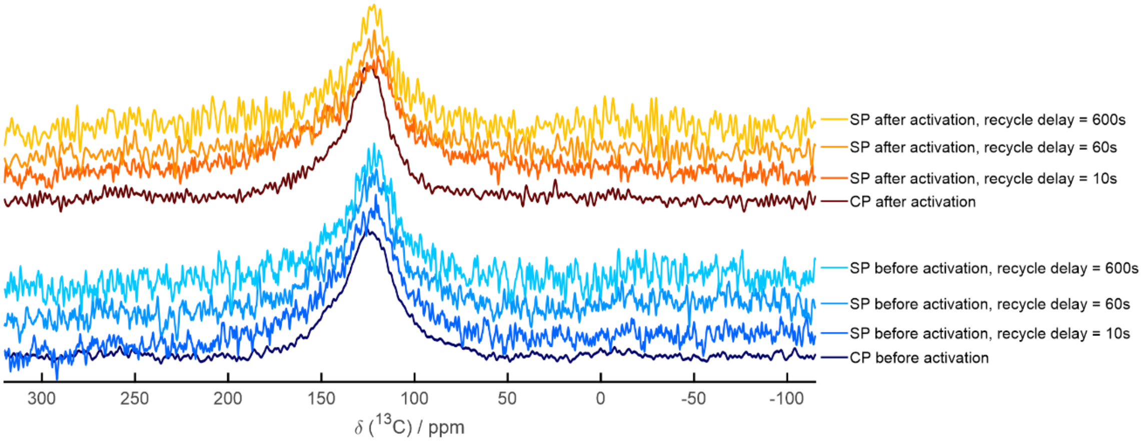

Figure S12: ^{13}C Single Pulse (SP) and Cross Polarization (CP) NMR spectra for samples before and after activation with NaOH. The similar line shapes of the SP and CP ^{13}C NMR spectra before and after activation suggest a regular distribution of proton spin density over the sample.

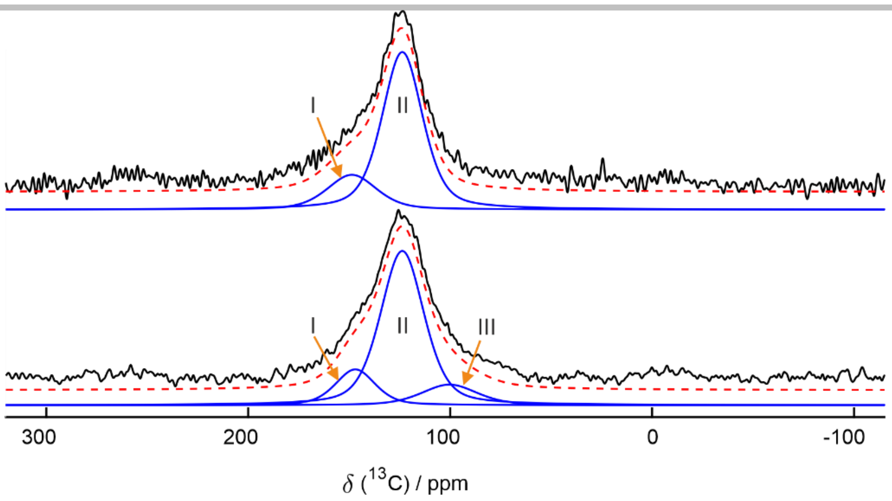

Figure S13: Deconvolution of ^{13}C CP MAS NMR spectra before (bottom) and after (top) activation with NaOH. The spectral regions I, II and III are characteristic for graphitic carbon with nitrogen incorporation, graphitic carbon without nitrogen and graphitic carbon in contact to silicon, respectively. The disappearance of signal region III is in line with the removal of silicon rich domains by activation with NaOH, as pointed out in Figure 2A. The similar intensity ratio for regions I and II show that the graphitic regions are insensitive to the activation procedure.

Table S4: Refinement parameters for deconvolution of ^{13}C CP MAS NMR spectra in Figure S13

	Before Activation			After Activation	
	I	II	III	I	II
δ / ppm	146.9	123.7	100.3	148.7	123.7
Intensity	0.15	0.73	0.12	0.21	0.79
G/L	0.6	0.6	0.6	0.6	0.6
FWHM / ppm	23.1	25.4	32.5	28.8	23.6

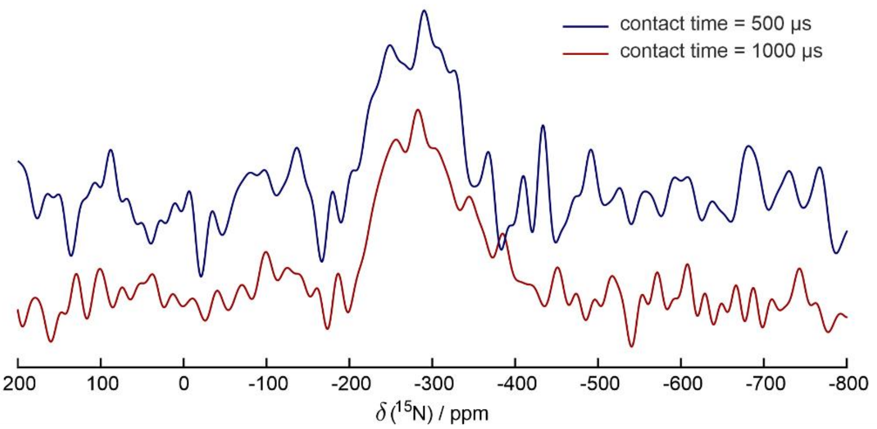

Figure S14: ^{15}N CPMAS NMR spectra with polarization transfer contact times of 500 µs (blue) and 1 ms (red). The similarity of both spectral shapes is indicative for close proton contacts for all nitrogen environments. The broad chemical shift range for the corresponding nitrogen atoms is typical for nitrogen embedded in graphitic domains.

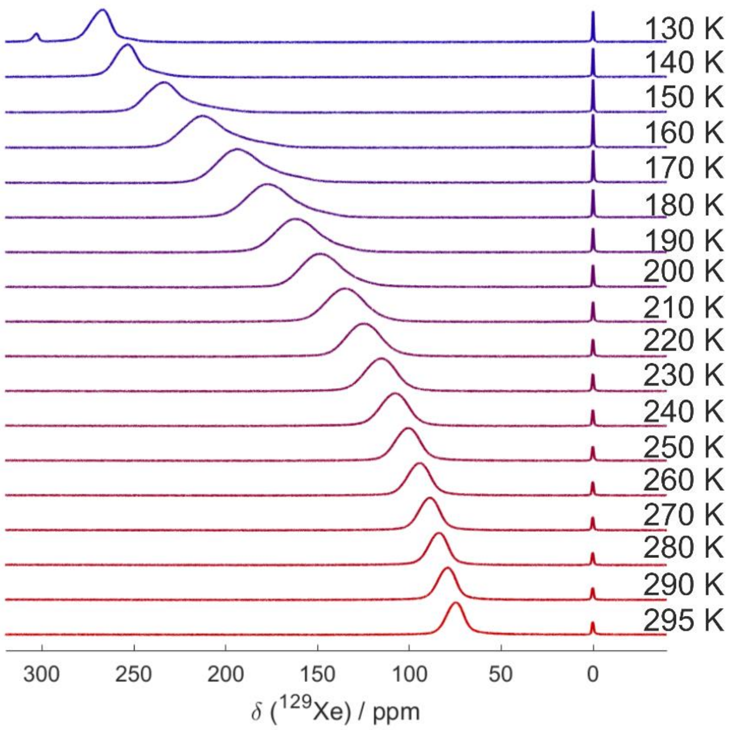

Figure S15: series of hyperpolarized (hp) ^{129}Xe NMR spectra at variable temperatures. The ^{129}Xe chemical shift (CS) in the low loading limit (high temperature range, 260 – 295 K) is typical for isolated xenon atoms in microporous graphitic materials. A detailed analysis of the shift in this region is given in Figure 2C. Towards lower temperatures the ^{129}Xe CS increases, corresponding to a larger xenon uptake, where also Xe-Xe interactions influence the chemical shift.

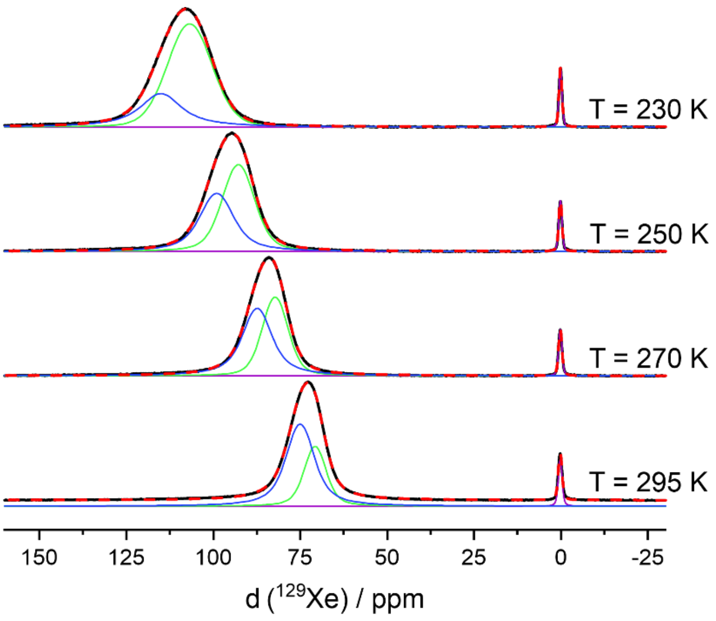

Figure S16: Depiction of the deconvolution of the limiting shift region of hp ^{129}Xe NMR spectra with a larger chemical shift range compared to Figure 2C.

4. Characterization of the isolated products

1-Phenylethanaminium chloride (1)

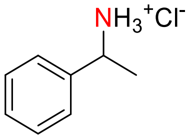

MW = 157.64 g/mol

^{1}H NMR (500 MHz, DMSO-D$_6$): δ = 8.55 (s, 3 H), 7.55 (d, *J* = 7.55 Hz, 2 H), 7,44-7,32 (m, 3 H), 4.41-4.30 (q, *J* = 4.36, 1 H), 1.52 (d, *J* = 1.55 Hz, 3 H) ppm

^{13}C NMR (126 MHz, DMSO-D$_6$): δ = 140.15, 129.10, 128.72, 127.31, 50.47, 21.43 ppm

Yield: 99 % (78 mg) as a white solid

1-Phenylpropan-1-aminium chloride (2)

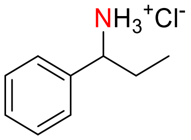

MW = 171.67 g/mol

^{1}H NMR (500 MHz, DMSO-D$_6$): δ = 8.64 (s, 3 H), 7.53-7.34 (m, 5 H), 4.3 (m, 1 H), 2.23-1.45 (m, 2 H), 0.79-0.70 (t, *J* = 0.75 Hz, 3 H) ppm

^{13}C NMR (126 MHz, DMSO-D$_6$): δ = 138.24, 129.13, 128.92, 127.96, 56.27, 27.95, 10.49 ppm

Yield: 99 % (85 mg) as a white solid

1-Phenylhexan-1-aminium chloride (3)

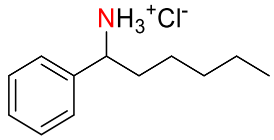

MW = 213.75 g/mol

^{1}H NMR (500 MHz, DMSO-D$_6$): δ = 8.49 (s, 3 H), 7.70-7.35 (m, 5 H), 3.04-2.96 (t, *J* = 3.01 Hz, 1 H), 1.64-1.56 (m, 2 H), 1.34-1.06 (m, 5 H), 0.87-0.77 (m, 3 H) ppm

^{13}C NMR (126 MHz, DMSO-D$_6$): δ = 137.19, 133.48, 129.16, 128.32, 38.35, 31.62, 28.79, 24.25, 22.50, 14.39 ppm

Yield: 84 % (90 mg) as a white solid

1-(4'-Fluorophenyl)ethanaminium chloride (4)

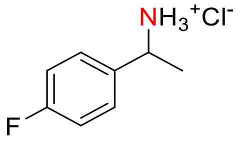

MW = 175.63 g/mol

^{1}H NMR (500 MHz, DMSO-D$_6$): δ = 8.75 (s, 3 H), 7.64-7.59 (q, *J* = 7.62 Hz, 2 H), 7.29-7.22 (t, *J* = 7.26 Hz, 2 H), 4.42-4.37 (q, *J* = 4.40 Hz, 1 H), 1,50 (d, *J* = 1.52 Hz, 3 H) ppm

^{13}C NMR (126 MHz, DMSO-D$_6$): δ = 161.40, 136.20, 129.73, 115.96, 49.76, 21.22 ppm

Yield: 98 % (86 mg) as a white solid

1-(4'-Chlorophenyl)ethanaminium chloride (5)

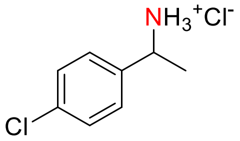

MW = 192.08 g/mol

^{1}H NMR (500 MHz, DMSO-D$_6$): δ = 8.79 (s, 3 H), 7.48 (d, *J* = 7.60 Hz, 2 H), 7.58 (d, *J* = 7.48 Hz, 2 H), 4.38-4.43 (q, *J* = 4.40 Hz, 1 H), 1.51 (d, *J* = 1.51 Hz, 3 H) ppm

^{13}C NMR (126 MHz, DMSO-D$_6$): δ = 138.94, 133.38, 129.44, 129.04, 49.79, 21.14 ppm

Yield: 92 % (88 mg) as a white solid

1-(4‘-Bromophenyl)ethanaminium chloride (6)

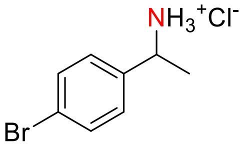

MW = 236.54 g/mol

^{1}H NMR (300 MHz, DMSO-D$_6$): δ = 8.82 (s, 3 H), 7.87 (d, *J* = 7.87 Hz, 2 H), 7.70 (d, *J* = 7.72 Hz, 2 H), 4.42-4.35 (m, 1 H), 1.52 (d, *J* = 1.52 Hz, 3 H) ppm

^{13}C NMR (75 MHz, DMSO-D$_6$): δ = 139.30, 132.18, 130.64, 121.99, 49.90, 27.17 ppm

Yield: 91 % (108 mg) as a white solid

1-(3‘-Chloropheny)lethanaminium chloride (7)

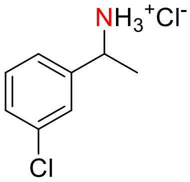

MW = 192.08 g/mol

^{1}H NMR (500 MHz, DMSO-D$_6$): δ = 8.75 (s, 3 H), 7.96-7.41 (m, 4 H), 4.46-4.39 (m, 1 H), 1.53 (d, *J* = 1.52 Hz, 3 H) ppm

^{13}C NMR (126 MHz, DMSO-D$_6$): δ = 142.36, 133.37, 131.20, 128.29, 127.44, 49.90, 27.35 ppm

Yield: 90 % (86 mg) as a white solid

1-(4-(Trifluoromethyl)phenyl)ethanaminium chloride (8)

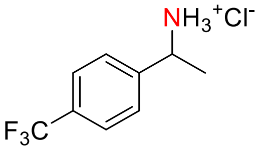

MW = 225.64 g/mol

^{1}H NMR (300 MHz, DMSO-D$_6$): δ = 8.85 (s, 3 H), 7.87-7.77 (s, 4 H), 4.63-4.45 (m, 1 H), 1.53 (d, *J* = 1.53 Hz, 3 H) ppm

^{13}C NMR (75 MHz, DMSO-D$_6$): δ = 144.53, 128.35, 126.03, 125.98, 50.00, 21.10 ppm

Yield: 97 % (109 mg) as a white solid

1-(4'-Methylphenyl)ethanaminium chloride (9)

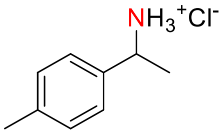

MW = 171.67 g/mol

^{1}H NMR (500 MHz, DMSO-D$_6$): δ = 8.66 (s, 3 H), 7.42 (d, J = 7.41 Hz, 2 H), 7.22 (d, J = 7.22 Hz, 2 H), 4.33-4,27 (q, J = 4.32 Hz, 1 H), 2.28 (s, 3 H), 1.50 (d, J = 1.54 Hz, 3 H) ppm

^{13}C NMR (126 MHz, DMSO-D$_6$): δ = 138.09, 136.95, 129.60, 127.24, 50.19, 21.26, 21.16 ppm

Yield: 83 % (71 mg) as a white solid

1-(3'-Methylphenyl)ethanaminium chloride (10)

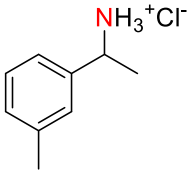

MW = 171.67 g/mol

^{1}H NMR (500 MHz, DMSO-D$_6$): δ = 8.74 (s, 3 H), 7.44-7.16 (m, 5 H), 4.34-4.29 (m, 1 H), 2.3 (s, 3 H), 1.53 (d, J = 1.51 Hz, 3 H) ppm

^{13}C NMR (126 MHz, DMSO-D$_6$): δ = 139.93, 138.25, 129.04, 127.91, 124.32, 50.45, 21.51, 21.32 ppm

Yield: 88 % (76 mg) as a white solid

1-(2'-Methylphenyl)ethanaminium chloride (11)

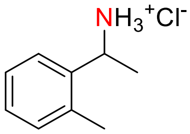

MW = 171.67 g/mol

^{1}H NMR (300 MHz, DMSO-D$_6$): δ = 8.74 (s, 3 H), 7.70-7.62 (m, 1 H), 7.35-7.21 (m, 3 H) 4.62-4.45 (m, 1 H), 2.34 (s, 3 H), 1.53 (d, J = 1.51 Hz, 3 H) ppm

^{13}C NMR (75 MHz, DMSO-D$_6$): δ = 138.34, 135.52, 130.96, 128.48, 126.94, 125.93, 46.65, 20.84, 19.27 ppm

Yield: 88 % (76 mg) as a white solid

1-(4‘-Methoxyphenyl)ethanaminium chloride (12)

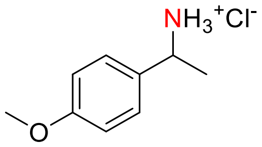

MW = 187.67 g/mol

^{1}H NMR (300 MHz, DMSO-D$_6$): δ = 8.58 (s, 3 H), 7.95 (d, J = 7.45 Hz, 2 H), 7.02 (d, J = 7.19 Hz, 2 H), 4.33-4.27 (m, 1 H), 3.85 (s, 3 H), 1.50 (d, J = 1.54 Hz, 3 H) ppm

^{13}C NMR (75 MHz, DMSO-D$_6$): δ = 163.56, 159.66, 130.95, 114.40, 55.99, 49.92, 26.88 ppm

Yield: 89 % (84 mg) as a white solid

1-(3‘-Methoxyphenyl)ethanaminium chloride (13)

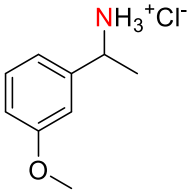

MW = 187.67 g/mol

^{1}H NMR (500 MHz, DMSO-D$_6$): δ = (500 MHz, DMSO-D$_6$): δ = 8.68 (s, 3 H), 7.36-7.29 (m, 1 H), 7.22-7.17 (m, 1 H), 7.08 (d, J = 7.52, 1 H), 6.95 (d, J = 7.56, 1 H), 4.34-4.29 (m, 1 H), 3.72 (s, 3 H), 1.53 (d, *J* = 1.56 Hz, 3 H) ppm

^{13}C NMR (126 MHz, DMSO-D$_6$): δ = 159.89, 141.53, 130.24, 119.31, 114.20, 113.05, 55.69, 50.44, 21.34 ppm

Yield: 90 % (84 mg) as a white solid

1-(3',4'-dimethoxyphenyl)ethanaminium chloride (14)

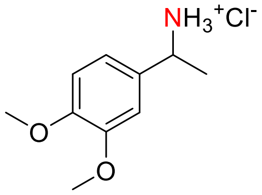

MW = 217.69 g/mol

^{1}H NMR (300 MHz, DMSO-D$_6$): δ = 8.51 (s, 3 H), 7.30-7.26 (m, 1 H), 7.06-6.97 (m, 2 H), 4.42-4.27 (m, 1 H), 3,82 (s, 3 H), 3.77 (s, 3 H), 1,48 (d, J = 7.5 Hz, 3 H) ppm

^{13}C NMR (75 MHz, DMSO-D$_6$): δ = 149.25, 132.16, 119.52, 112.15, 111.37, 56.11, 50.33, 21.21 ppm

Yield: 88 % (96 mg) as a white solid

diphenylmethanaminium chloride (15)

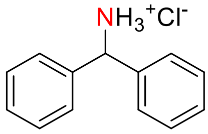

MW = 219.71 g/mol

^{1}H NMR (300 MHz, DMSO-D$_6$): δ = 9.26 (s, 3 H), 7.94-7.37 (m, 10 H), 5.77-5.50 (m, 1 H) ppm

^{13}C NMR (75 MHz, DMSO-D$_6$): δ = 138.84, 135.72, 132.19, 131.39, 129.60, 129.22, 128.73, 127.84, 57.55 ppm

Yield: 80 % (88 mg) as a white solid

1-(Pyridin-4-yl)ethanaminium chloride (16)

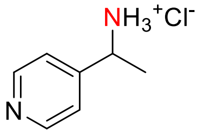

MW = 158.63 g/mol

^{1}H NMR (300 MHz, DMSO-D$_6$): δ = 9.22 (s, 3 H), 9.04-8,98 (d, *J* = 6.9, 2 H), 8.22 (d, *J* = 6.8, 2 H), 4.86-4.59 (m, 1 H), 1.58 (d, J = 7.5 Hz, 3 H) ppm

^{13}C NMR (75 MHz, DMSO-D$_6$): δ = 159.05, 142.32, 125.89, 49.57, 20.62 ppm

Yield: 91 % (72 mg) as a yellow solid

2,3-dihydro-1H-inden-1-aminium chloride (17)

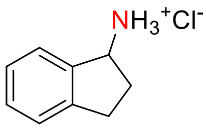

MW = 169.65 g/mol

^{1}H NMR (300 MHz, DMSO-D$_6$): δ = 8.65 (s, 3 H), 7.69 (d, *J* = 6.8, 2 H), 7.40-7.25 (m, 3 H), 4.78-4.63 (m, 1 H), 3.19-3.02 (m, 1 H), 2.97-2.81 (m, 1 H), 2.51-2.40 (m, 1 H), 2.12-1.92 (m, 1 H) ppm

^{13}C NMR (75 MHz, DMSO-D$_6$): δ = 144.39, 139.86, 129.39, 127.08, 125.51, 125.33, 55.04, 30.77, 30.31 ppm

Yield: 62 % (53 mg) as a white solid

1-(4-fluorophenyl)-benzeneethanaminium chloride (18)

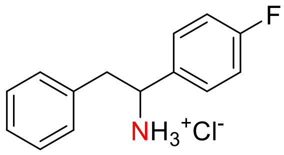

MW = 251.73 g/mol

^{1}H NMR (300 MHz, DMSO-D$_6$): δ = 8.78 (s, 3 H), 7.58-7.46 (m, 2 H), 7.29-7.14 (m, 5 H), 7.12-7.05 (m, 2 H), 4.63-4.48 (m, 1 H), 3.51-3.39 (m, 1 H), 3.20-3.06 (m, 1 H) ppm

^{13}C NMR (75 MHz, DMSO-D$_6$): δ = 160.82, 136.55, 133.80, 130.65, 130.54, 129.72, 128.79, 127.15, 115.91, 115.63, 55.45 ppm

Yield: 74 % (93 mg) as a white solid

Phenylmethanaminium chloride (19)

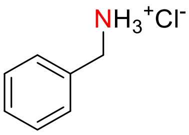

MW = 143.61 g/mol

^{1}H NMR (300 MHz, DMSO-D$_6$): δ = 8.71 (s, 3 H), 7.59-7.51 (m, 2 H), 7.47-7.36 (m, 3 H), 4.01 (s, 2 H) ppm

^{13}C NMR (75 MHz, DMSO-D$_6$): δ = 134.62, 129.46, 128.98, 128.81, 52.56 ppm

Yield: 92 % (66 mg) as a white solid

p-tolylmethanaminium chloride (20)

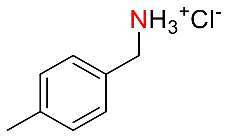

MW = 157.64 g/mol

^{1}H NMR (300 MHz, DMSO-D$_6$): δ = 8.54 (s, 3 H), 7.40 (d, *J* = 7.5 Hz, 2 H), 7.24 (d, *J* = 7.5 Hz, 2 H), 4.04-3.91 (m, 2 H), 2.32 (s, 3 H) ppm

^{13}C NMR (75 MHz, DMSO-D$_6$): δ = 138.22, 131.54, 129.57, 129.39, 42.38, 21.23 ppm

Yield: 85 % (67 mg) as a white solid

(4-Methoxyphenyl)methanaminium chloride (21)

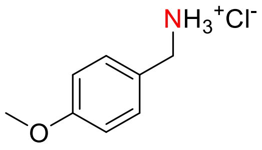

MW = 173.64 g/mol

^{1}H NMR (300 MHz, DMSO-D$_6$): δ = 8.51 (s, 3 H), 7.47 (d, *J* = 7.5 Hz, 2 H), 6.98 (d, *J* = 7.5 Hz, 2 H), 4.01-3.88 (m, 2 H), 3.79-3.77 (s, 3 H) ppm

^{13}C NMR (75 MHz, DMSO-D$_6$): δ = 159.79, 131.04, 126.48, 114.35, 55.69, 42.09 ppm

Yield: 88 % (76 mg) as a white solid

(4-Fluorophenyl)methanaminium chloride (22)

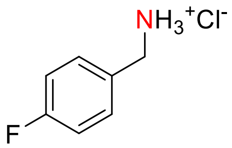

MW = 161.6 g/mol

^{1}H NMR (300 MHz, DMSO-D$_6$): δ = 8.67 (s, 3 H), 7.65-7.57 (m, 2 H), 7.30-7.23 (m, 2 H), 4.07-3.97 (s, 2 H) ppm

^{13}C NMR (75 MHz, DMSO-D$_6$): δ = 164.16, 131.92, 131.81, 130.92, 115.94, 115.66, 41.83 ppm

Yield: 84 % (68 mg) as a white solid

(4-Chlorophenyl)methanaminium chloride (23)

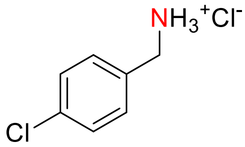

MW = 178.06 g/mol

^{1}H NMR (300 MHz, DMSO-D$_6$): δ = 8.75 (s, 3 H), 7.63-7.56 (m, 2 H), 7.52-7.45 (m, 2 H), 4.08-3.98 (s, 2 H) ppm

^{13}C NMR (75 MHz, DMSO-D$_6$): δ = 133.62, 133.53, 131.51, 128.91, 41.83 ppm

Yield: 91 % (81 mg) as a white solid

Pentan-aminium chloride (24)

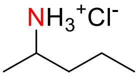

MW = 123.62 g/mol

^{1}H NMR (500 MHz, DMSO-D$_6$): δ = 8.12 (s, 3 H), 3.16-3.08 (m, 1 H), 1.60-1.11 (m, 7 H), 0.92-0.84 (t, J = 0.88, 3 H) ppm

^{13}C NMR (126 MHz, DMSO-D$_6$): δ = 46.95, 36.70, 18.57, 14.16 ppm

Yield: 36 % (22 mg) as a white solid

Dodean-2-aminium chloride (25)

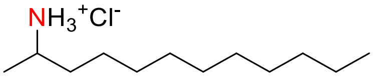

MW = 221.81 g/mol

^{1}H NMR (500 MHz, DMSO-D$_6$): δ = 8.00 (s, 3 H), 3.20-3.05 (m, 1 H), 1.62-1.15 (m, 21 H), 0.88-0.82 (t, *J* = 0.86, 3 H) ppm

^{13}C NMR (126 MHz, DMSO-D$_6$): δ = 47.19, 34.56, 31.77, 29.47, 29.41, 29.32, 29.24, 29.18, 25.23, 22.58, 18.58, 14.44 ppm

Yield: 88 % (98 mg) as a white solid

Heptan-4-aminium chloride (26)

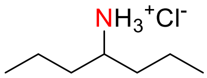

MW = 151.68 g/mol

^{1}H NMR (500 MHz, DMSO-D$_6$): δ = 8.24 (s, 3 H), 3.06-2.97 (m, 1 H), 1.56-1.28 (m, 8 H), 0.88-0.83 (m, 6 H) ppm

^{13}C NMR (126 MHz, DMSO-D$_6$): δ = 50.66, 34.54, 18.28, 14.25 ppm

Yield: 87 % (66 mg) as a white solid

3-Methyl-2-Butylaminium chloride (27)

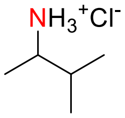

MW = 123.62 g/mol

^{1}H NMR (500 MHz, DMSO-D$_6$): δ = 7.99 (s, 3 H), 3.04-2.98 (m, 1 H), 1.86-1.77 (m, 1 H), 1.11 (d, J = 1.12 Hz, 3 H), 0.94-0.84 (m, 6 H) ppm

^{13}C NMR (126 MHz, DMSO-D$_6$): δ = 52.15, 31.28, 19.21, 17.43, 15.10 ppm

Yield: 58 % (36 mg) as a white solid

4-Methyl-2-Pentylaminium chloride (28)

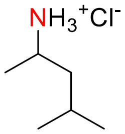

MW = 137.65 g/mol

^{1}H NMR (500 MHz, DMSO-D$_6$): δ = 8.08 (s, 3 H), 3.21-3.10 (m, 1 H), 1.71-1.62 (m, 1 H), 1.50-1.42 (m, 1 H), 1.34-1.28 (m, 1 H), 1.19-1.14 (d, J = 1.18 Hz, 3 H), 0.91-0.84 (m, 6 H) ppm

^{13}C NMR (126 MHz, DMSO-D$_6$): δ = 45.46, 43.72, 24.27, 23.33, 22.23, 18.80 ppm

Yield: 93 % (64 mg) as a white solid

1-Methylbenzylpropylaminium chloride (29)

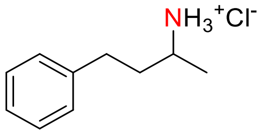

MW = 185.70 g/mol

^{1}H NMR (500 MHz, DMSO-D$_6$): δ = 8.26 (s, 3 H), 7.32-7.16 (m, 5 H), 3.16-3.07 (m, 1 H), 2.72-2.58 (m, 2 H), 1.97-1.88 (m, 1 H), 1.99-1.91 (m, 1 H), 1.24 (d, *J* = 1.23 Hz, 3 H) ppm

^{13}C NMR (126 MHz, DMSO-D$_6$): δ = 141.50, 128.88, 128.70, 126.45, 46.84, 44.60, 30.22, 18.49 ppm

Yield: 91 % (85 mg) as a white solid

Cyclopentylaminium chloride (30)

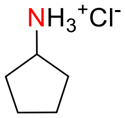

MW = 121.61 g/mol

^{1}H NMR (500 MHz, DMSO-D$_6$): δ = δ = 8.08 (s, 3 H), 3.48-3.40 (m, 1 H), 1.94-1.83 (m, 2 H), ppm 1.72-1.63 (m, 2 H), 1.59-1.44 (m, 2 H) ppm

^{13}C NMR (126 MHz, DMSO-D$_6$): δ = 51.58, 31.08, 23.91 ppm

Yield: 49 % (30 mg) as a white solid

Cyclohexylaminium chloride (31)

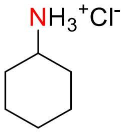

MW = 135.64 g/mol

^{1}H NMR (500 MHz, DMSO-D$_6$): δ = 8.18 (s, 3 H), 2.95-2.87 (m, 1 H), 1.94-1.04 (m, 11 H) ppm

^{13}C NMR (126 MHz, DMSO-D$_6$): δ = 49.69, 30.72, 24.21, 24.18 ppm

Yield: 61 % (41 mg) as a white solid

Cyclododecylaminium chloride (32)

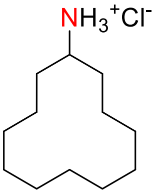

MW = 219.80 g/mol

^{1}H NMR (500 MHz, DMSO-D$_6$): δ = 8,17 (s, 3 H), 2,46-2,39 (m, 4 H), 1,66-1,58 (m, 4 H), 1,33-1,11 (m, 16 H) ppm

^{13}C NMR (126 MHz, DMSO-D$_6$): δ = 65.40, 48.24, 28.02, 24.68, 24.60, 24.22, 22.45 ppm

Yield: 77 % (85 mg) as a white solid

Nabumetone-$NH_3^+Cl^-$ (33)

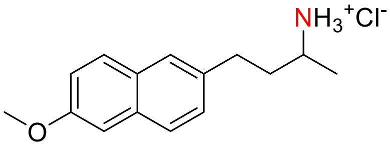

MW = 265.78 g/mol

^{1}H NMR (300 MHz, DMSO-D$_6$): δ = 8,22 (s, 3 H), 7.82-7.65 (m, 3 H), 7.40-7.27 (m, 2 H), 7.19-7.13 (m, 1 H), 3.88 (s, 3 H), 3.23-3.12 (m, 1 H), 2.92-2.70 (m, 1 H), 2.12-1.76 (m, 2 H), 1.32-1.29 (d, *J* = 6 Hz, 3 H) ppm

^{13}C NMR (75 MHz, DMSO-D$_6$): δ = 157.32, 136.54, 133.30, 129.27, 129.05, 128.04, 127.35, 126.43, 119.06, 106.30, 46.91, 36.28, 31.27, 15.57 ppm

Yield: 87 % (116 mg) as a white solid

1-(4-methoxyphenyl)propan-2-aminium chloride (34)

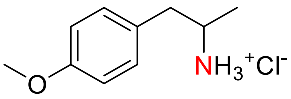

MW = 201.69 g/mol

^{1}H NMR (300 MHz, DMSO-D$_6$): δ = 8.19 (s, 3 H), 7.19 (d, *J* = 7.5 Hz, 2 H), 6.92 (d, *J* = 7.5 Hz, 2 H), 7.19-7.13 (m, 1 H), 3.77 (s, 3 H), 3.39-3.26 (m, 1 H), 3.08-2.94 (m, 1 H), 2.69-2.57 (m, 1 H), 1.12 (d, *J* = 6 Hz, 3 H) ppm

^{13}C NMR (75 MHz, DMSO-D$_6$): δ = 158.58, 130.75, 129.11, 114.46, 55.53, 48.63, 17.92 ppm

Yield: 90 % (91 mg) as a white solid

1-(2-methoxyphenyl)propan-2-aminium chloride (35)

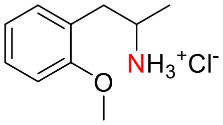

MW = 201.69 g/mol

^{1}H NMR (300 MHz, DMSO-D$_6$): δ = 8.21 (s, 3 H), 7.32-6.91 (m, 4 H), 3.81 (s, 3 H), 3.47-3.39 (m, 1 H), 3.10-2.95 (m, 1 H), 2.81-2.65 (m, 1 H), 1.10 (d, *J* = 6 Hz, 3 H) ppm

^{13}C NMR (75 MHz, DMSO-D$_6$): δ =157.77, 131.40, 128.86, 125.04, 120.87, 111.42, 55.84, 47.18, 35.38, 18.19 ppm

Yield: 73 % (74 mg) as a white solid

Stanolone-$NH_3^+Cl^-$ (36)

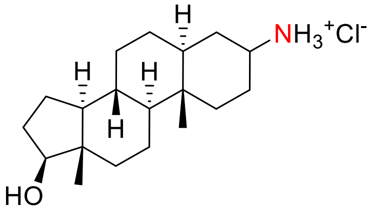

MW = 327.94 g/mol

^{1}H NMR (300 MHz, DMSO-D$_6$): δ = 8.14 (s, 3 H), 2.09-0.75 (m, 25 H), 0.63 (s, 3 H) ppm

^{13}C NMR (75 MHz, DMSO-D$_6$): δ = 80.55, 53.85, 51.31, 46.80, 44.71, 43.07, 38.57, 36.04, 35.57, 31.62, 31.31, 31.07, 30.39, 28.26, 23.52, 20.45, 11.78, 11.57 ppm

Yield: 54 % (89 mg) as a white solid

Further experiments with electron withdrawing substituents and α-keto acids:

The reductive amination of 4-nitro acetophenone couldn’t be accomplished due to the reduction of the nitro group to an amine group. The reductive amination of 4-acetamidoacetophenone, Methyl-4-acetylbenzoate and phenylglyoxylic acid delivered hardly any yield.

5. NMR Spectra

1:

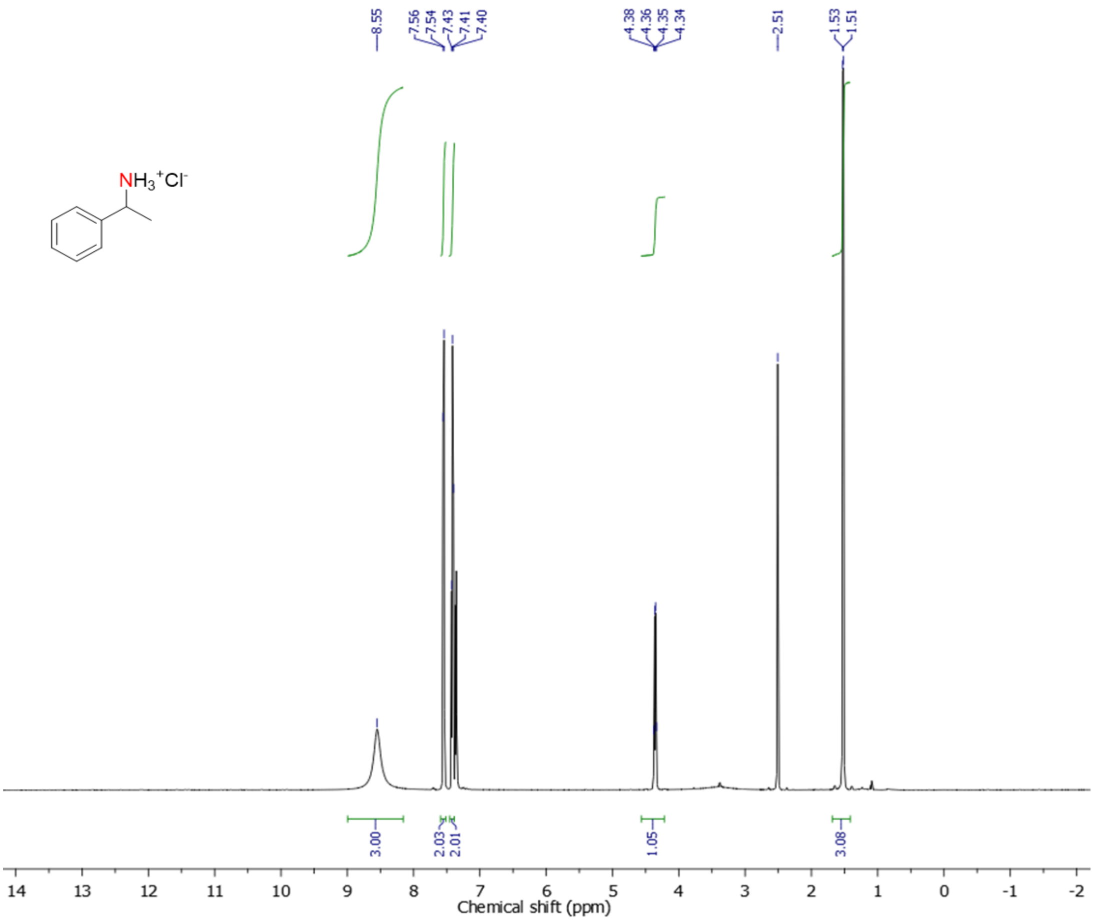

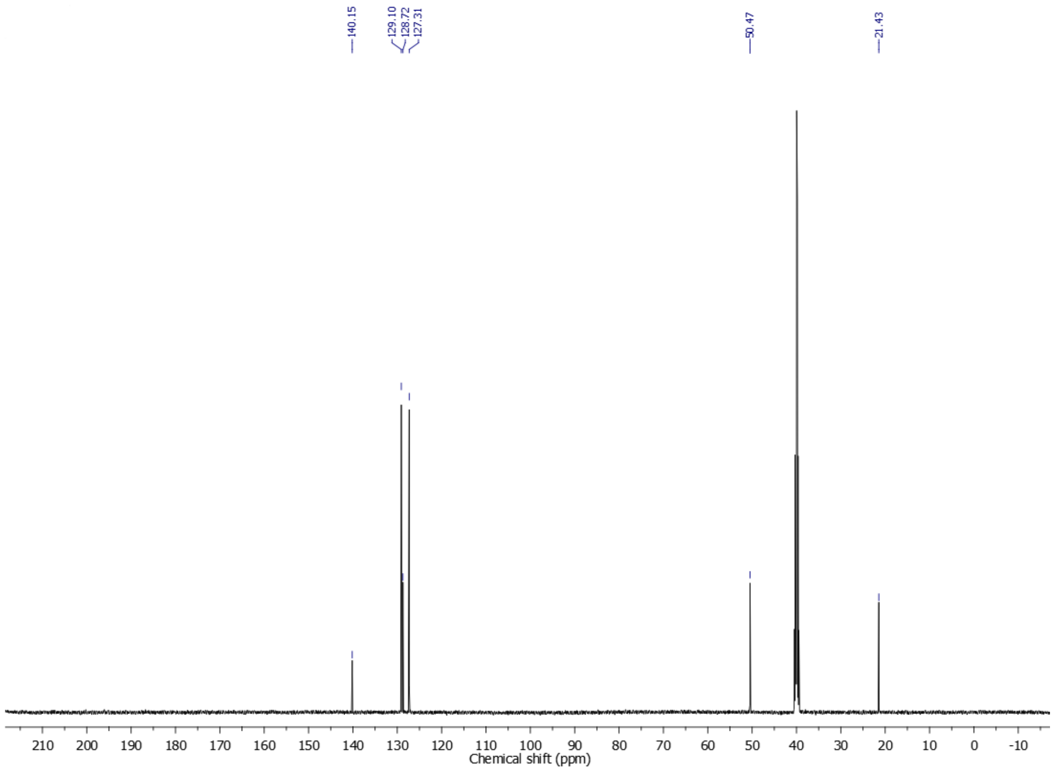
140.15
129.10
128.72
127.31
50.47
21.43
210 200 190 180 170 160 150 140 130 120 110 100 90 80 70 60 50 40 30 20 10 0 -10
Chemical shift (ppm)

2:

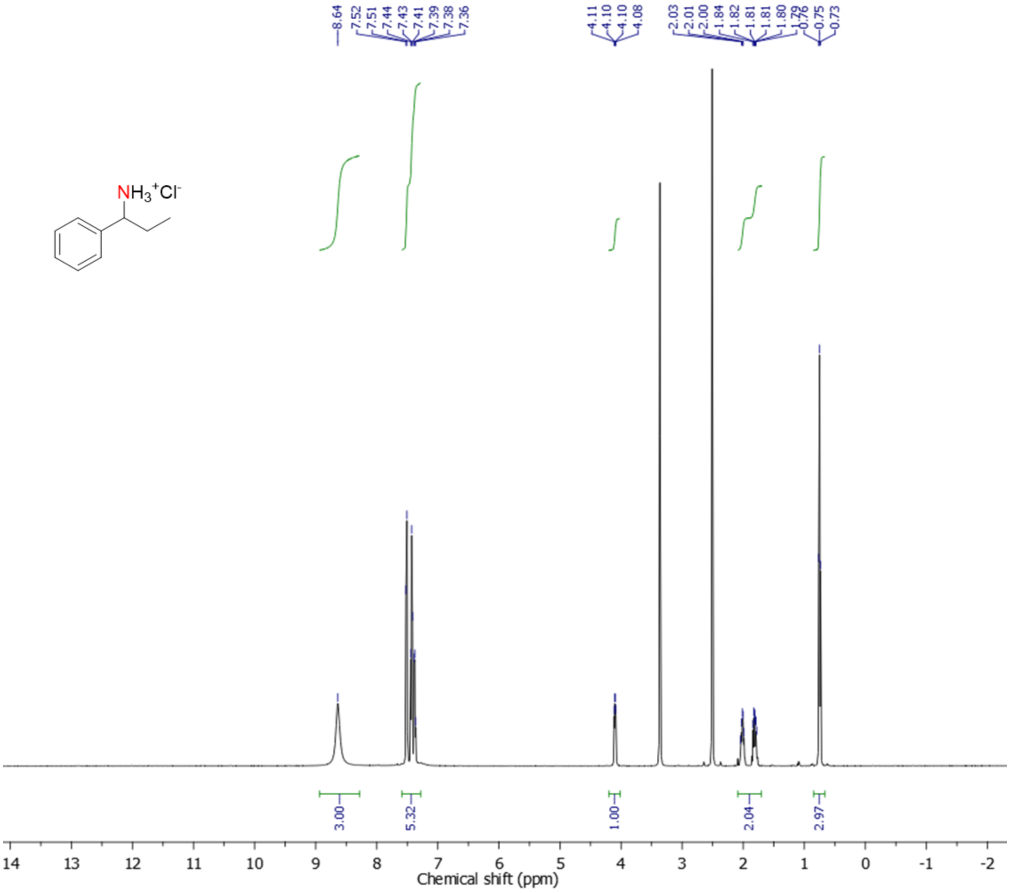
8.64
7.52
7.51
7.44
7.43
7.41
7.39
7.38
7.36
4.11
4.10
4.10
4.08
2.03
2.01
2.00
1.84
1.82
1.81
1.81
1.80
1.79
0.76
0.75
0.73
3.00
5.32
1.00
2.04
2.97
14 13 12 11 10 9 8 7 6 5 4 3 2 1 0 -1 -2
Chemical shift (ppm)

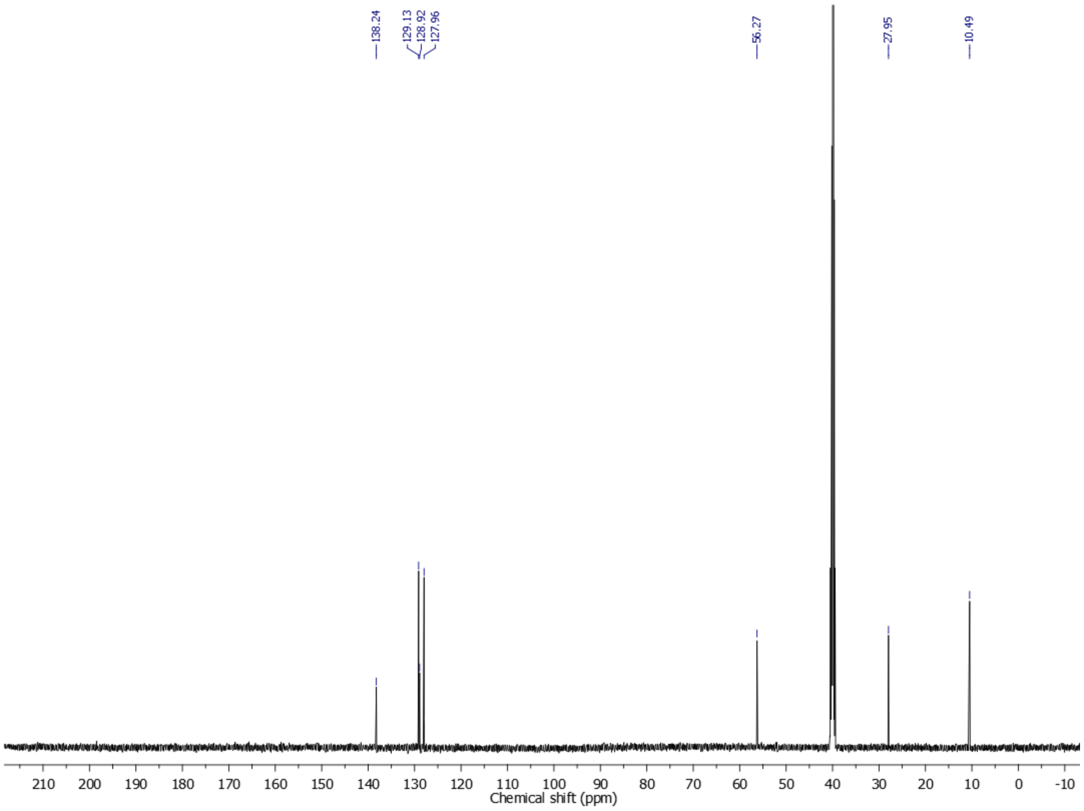
138.24
129.13
128.92
127.96
56.27
27.95
10.49
210 200 190 180 170 160 150 140 130 120 110 100 90 80 70 60 50 40 30 20 10 0 -10
Chemical shift (ppm)

3:

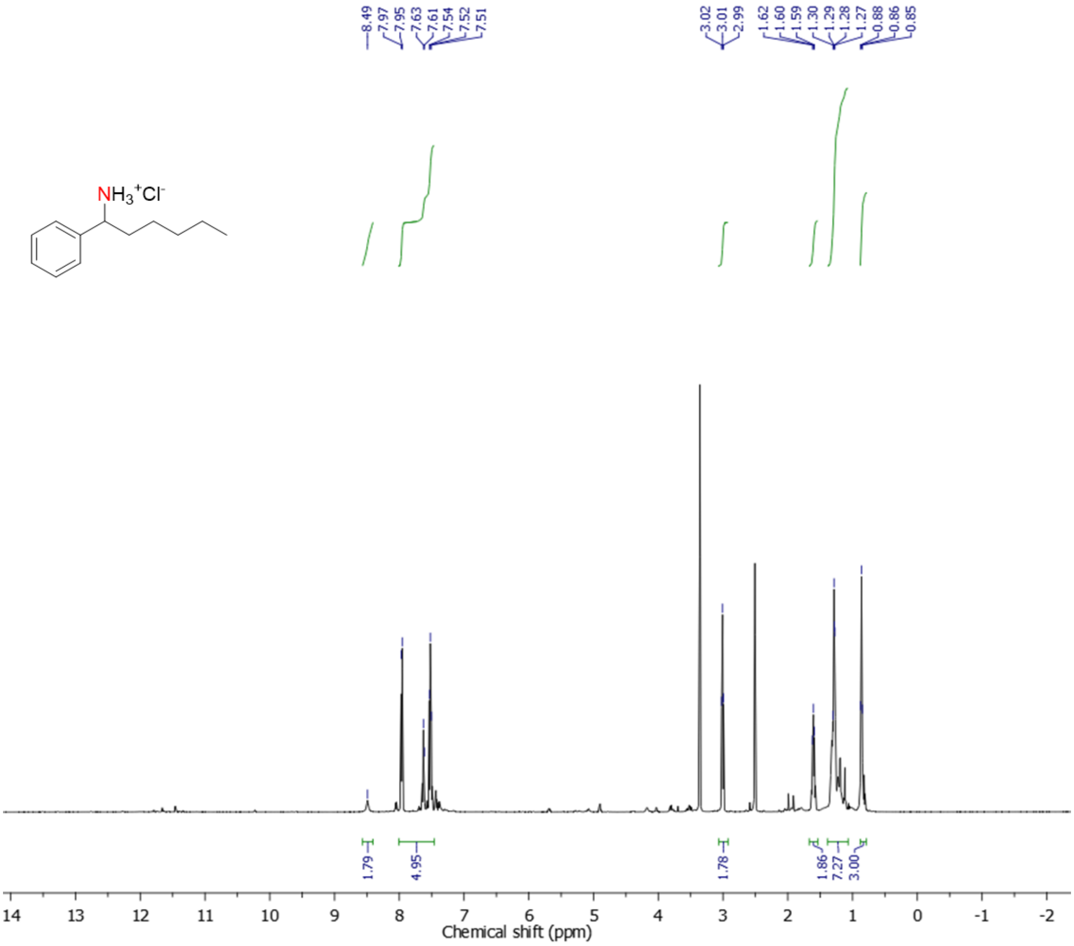
NH3+Cl-
8.49
7.97
7.95
7.63
7.61
7.54
7.52
7.51
3.02
3.01
2.99
1.62
1.60
1.59
1.30
1.29
1.28
1.27
0.88
0.86
0.85
1.79
4.95
1.78
1.86
7.27
3.00
Chemical shift (ppm)

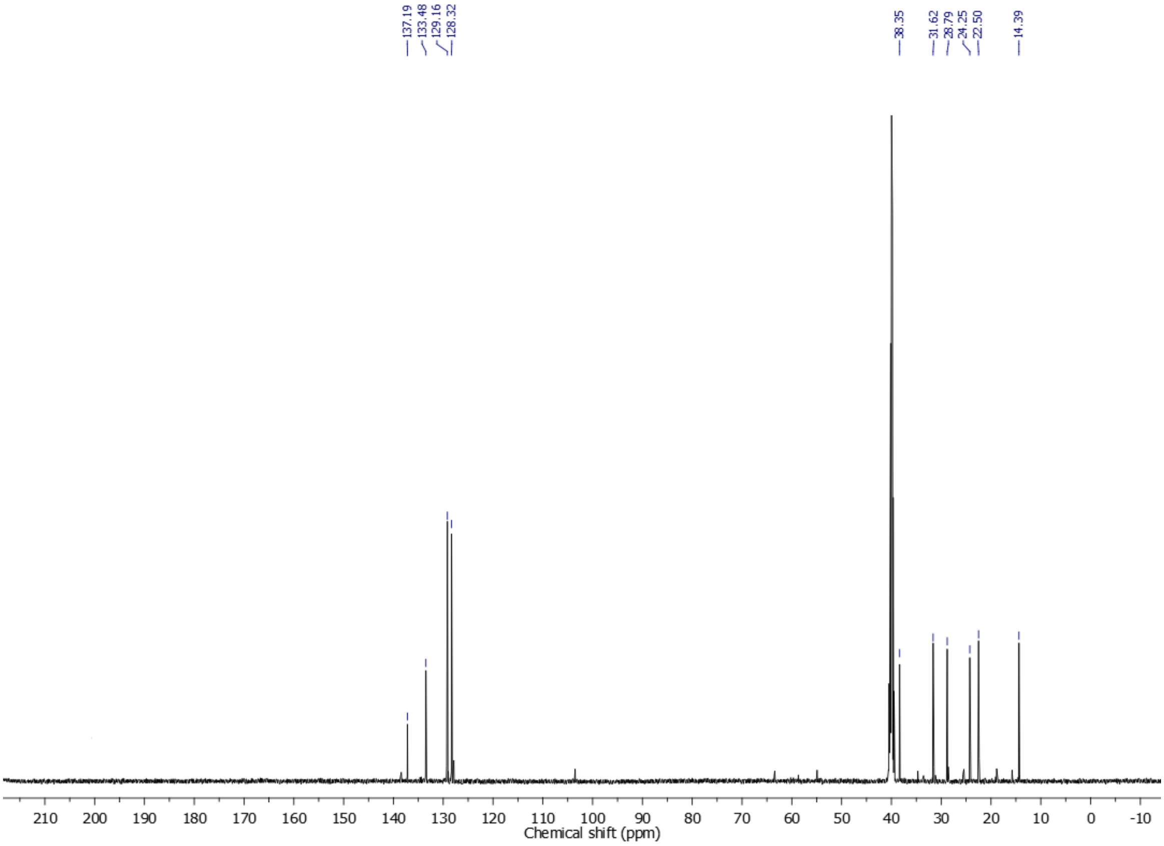
137.19
133.48
129.16
128.32
38.35
31.62
28.79
24.25
22.50
14.39
Chemical shift (ppm)

4:

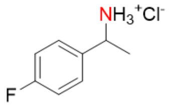
NH3+Cl-
F

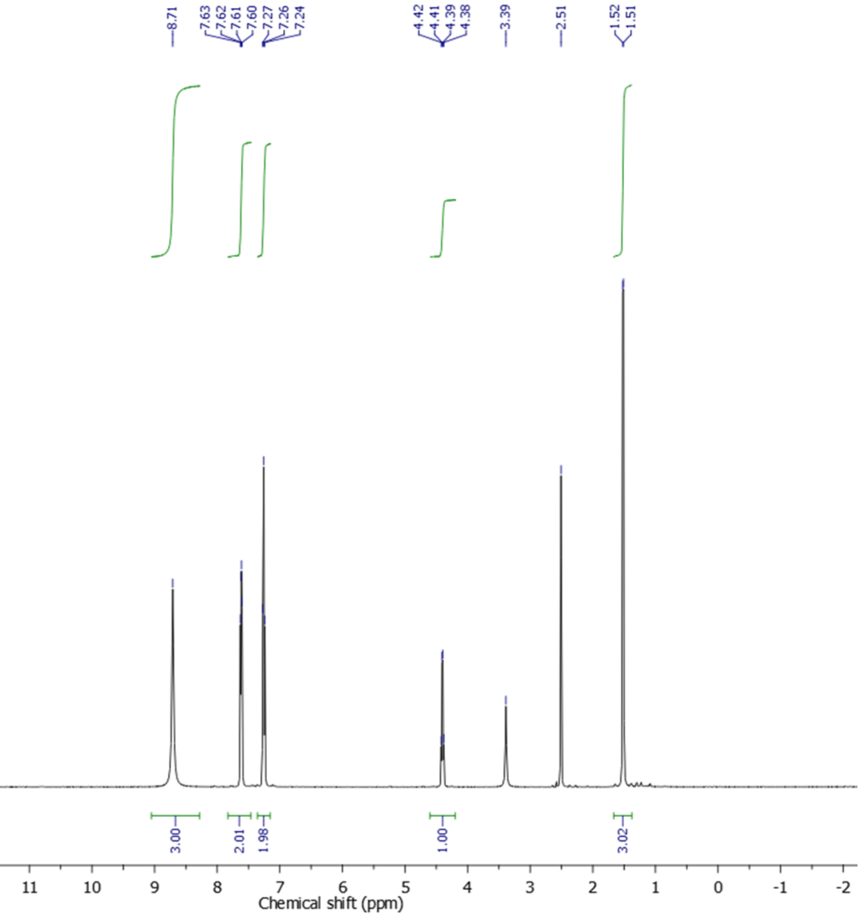
8.71
7.63
7.62
7.61
7.60
7.27
7.26
7.24
4.42
4.41
4.39
4.38
3.39
2.51
1.52
1.51
3.00
2.01
1.98
1.00
3.02
14 13 12 11 10 9 8 7 6 5 4 3 2 1 0 -1 -2
Chemical shift (ppm)

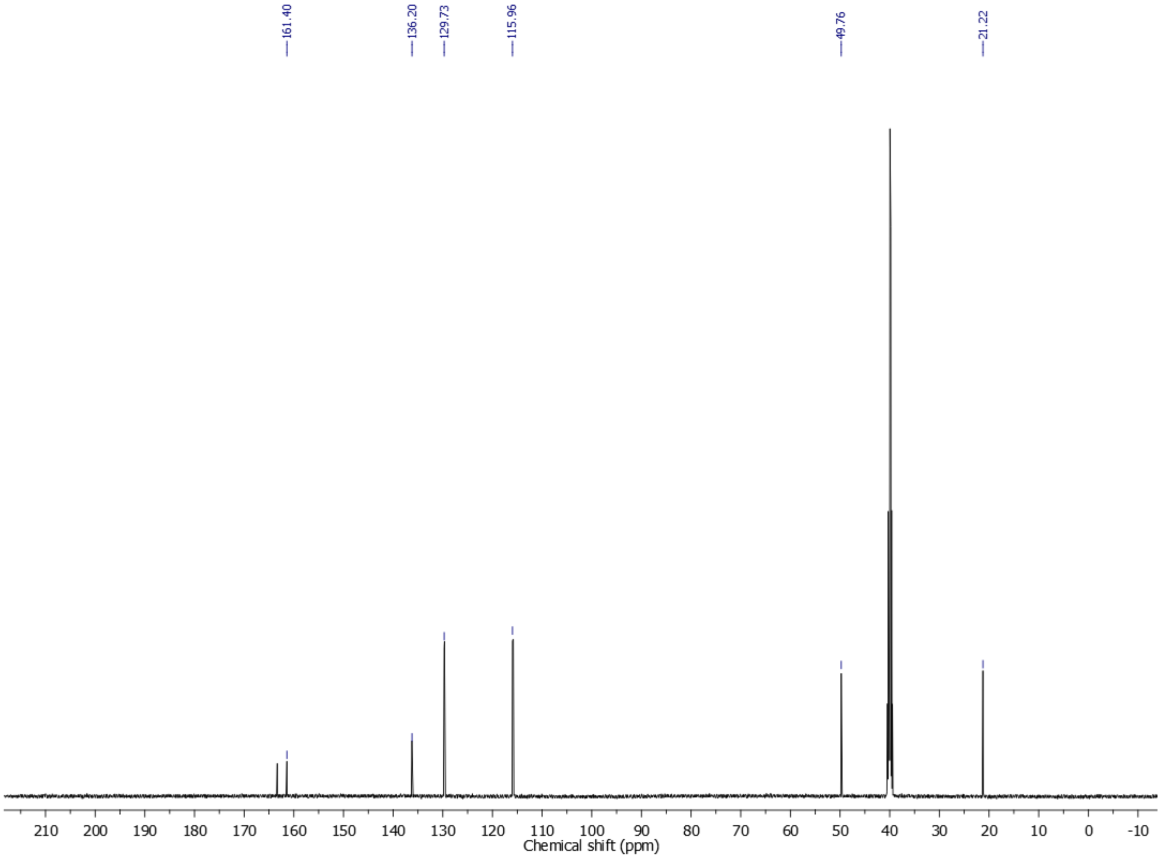
161.40
136.20
129.73
115.96
49.76
21.22
210 200 190 180 170 160 150 140 130 120 110 100 90 80 70 60 50 40 30 20 10 0 -10
Chemical shift (ppm)

5:

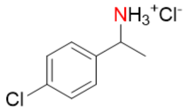
NH3+Cl-
Cl

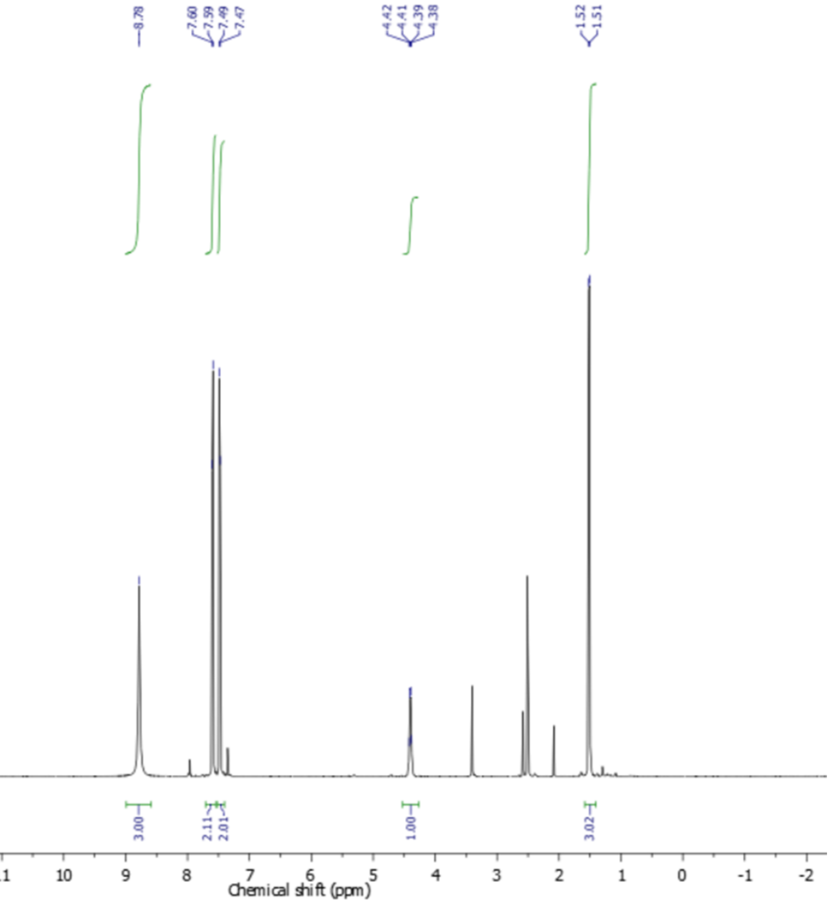
8.78
7.60
7.59
7.49
7.47
4.42
4.41
4.39
4.38
1.52
1.51
3.00
2.11
2.01
1.00
3.02
Chemical shift (ppm)

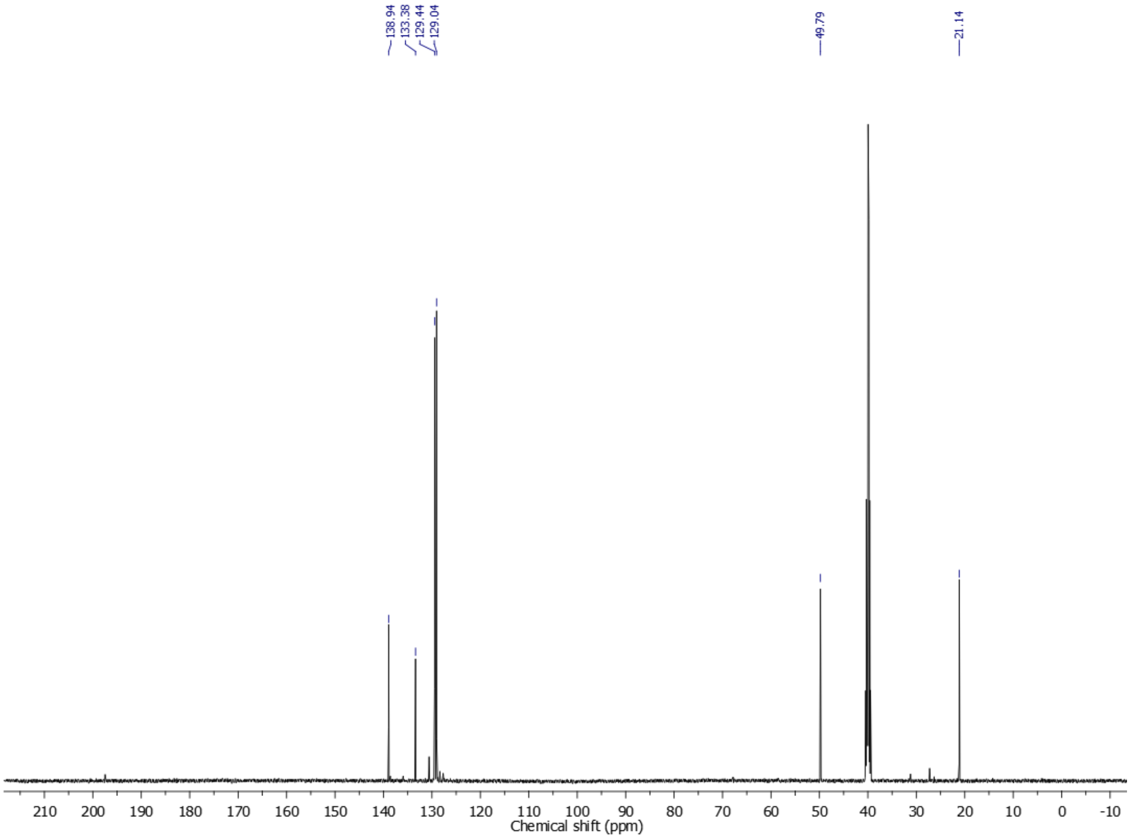
138.94
133.38
129.44
129.04
49.79
21.14
Chemical shift (ppm)

6:

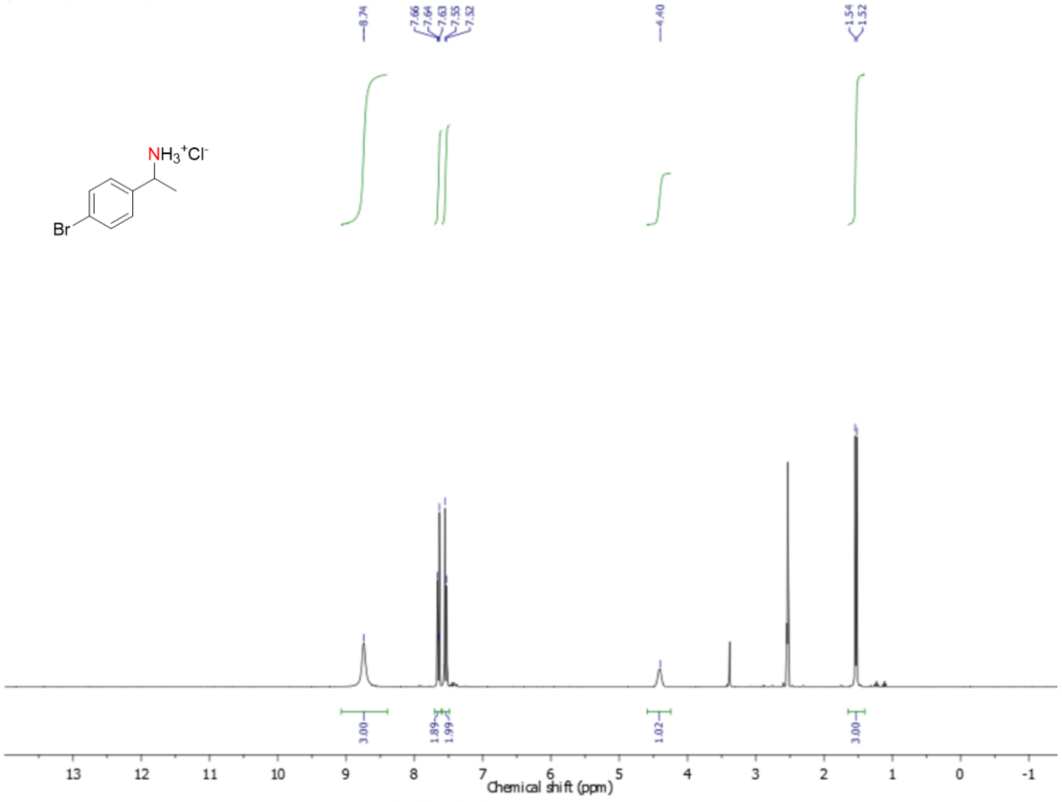
NH3+Cl-
Br
8.74
7.66
7.64
7.63
7.55
7.52
4.40
1.54
1.52
3.00
1.89
1.99
1.02
3.00
Chemical shift (ppm)

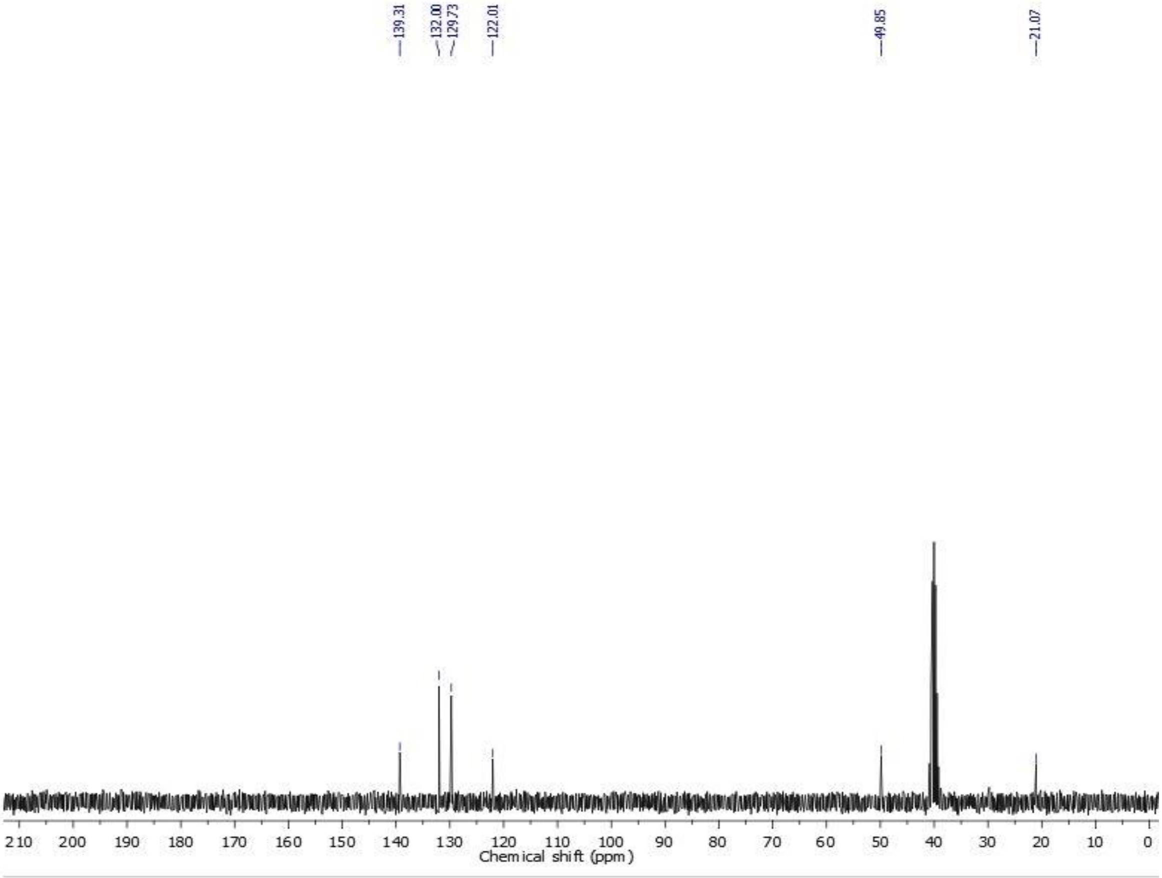
139.31
132.00
129.73
122.01
49.85
21.07
Chemical shift (ppm)

7:

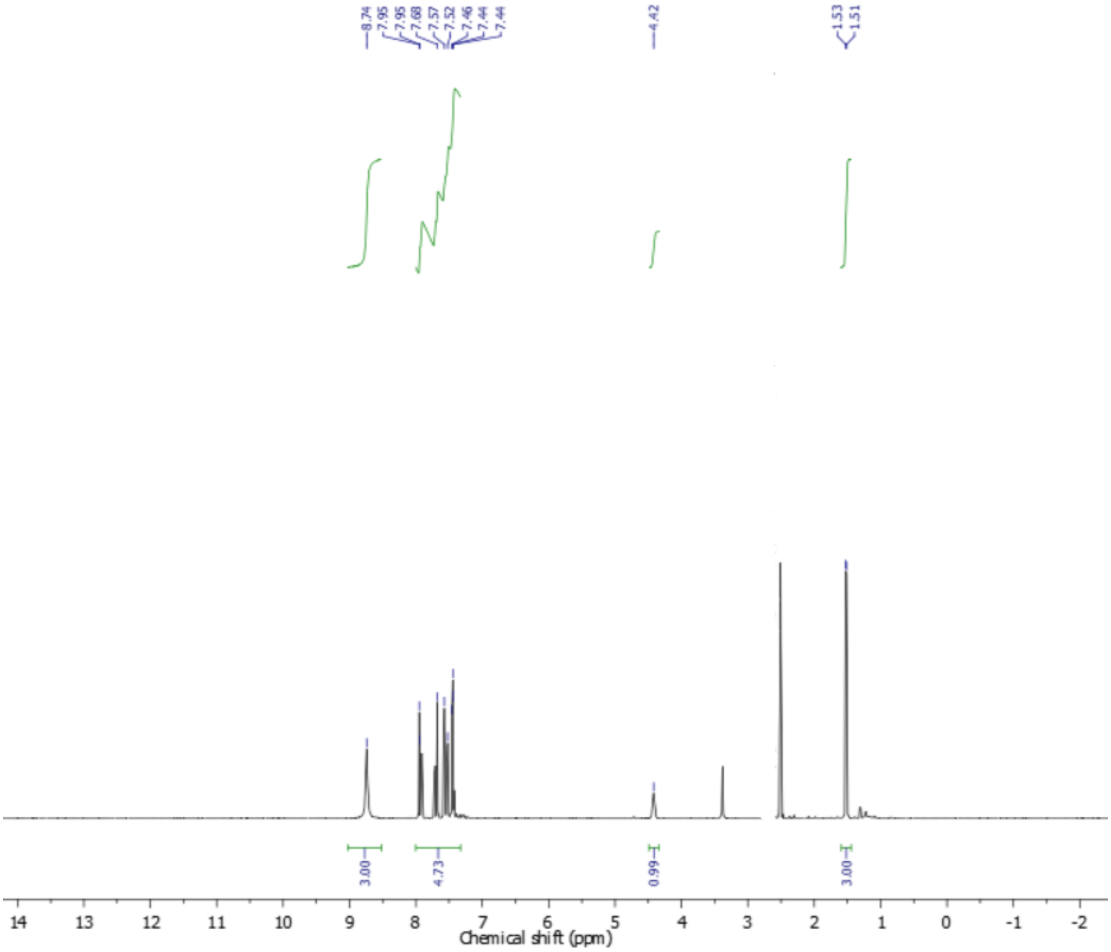
8.74
7.95
7.95
7.68
7.57
7.52
7.46
7.44
7.44
4.42
1.53
1.51
3.00
4.73
0.99
3.00
Chemical shift (ppm)

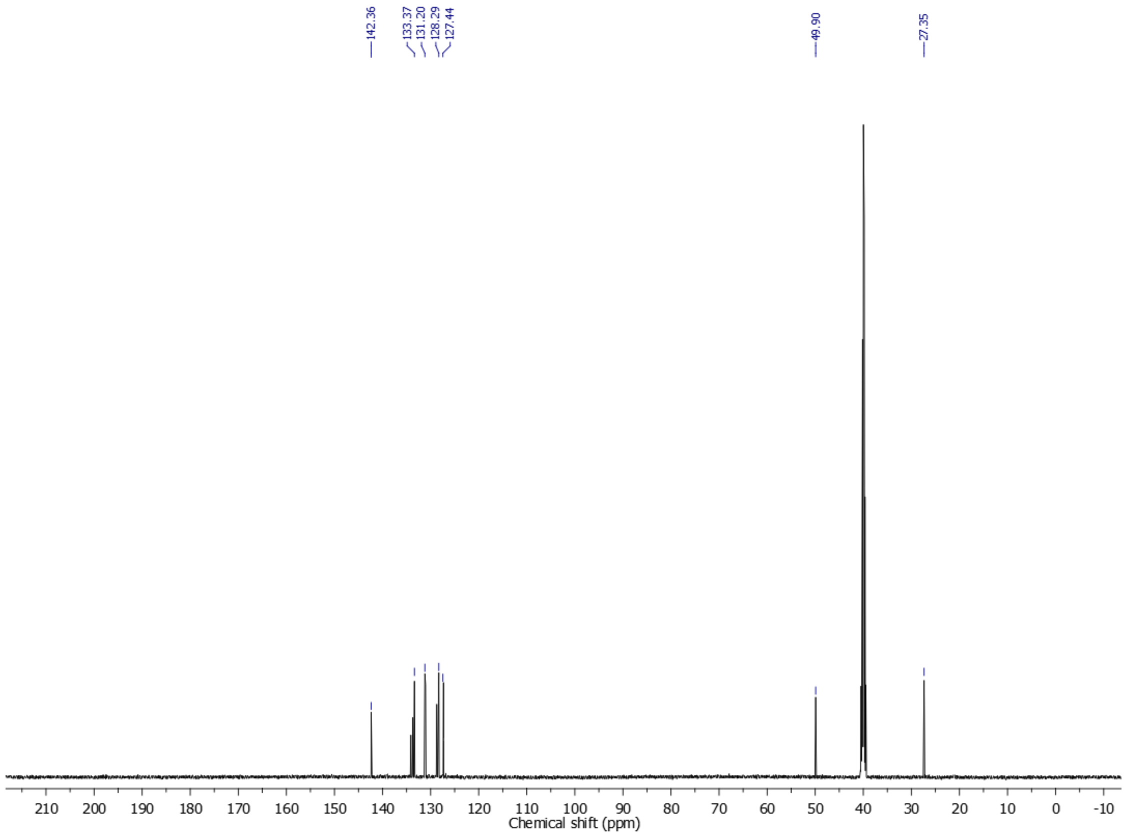
142.36
133.37
131.20
128.29
127.44
49.90
27.35
Chemical shift (ppm)

8:

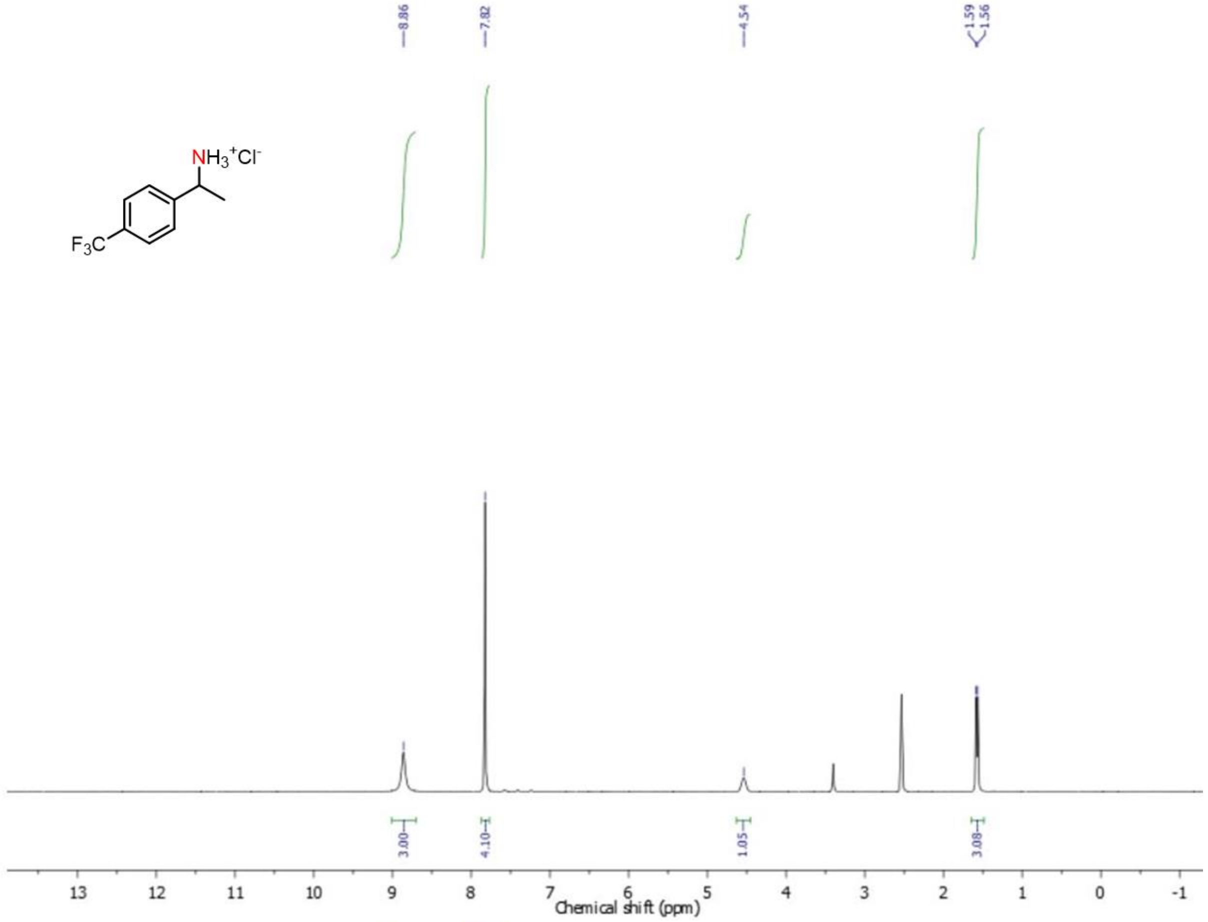
NH3+Cl-
F3C
8.86
7.82
4.54
1.59
1.56
3.00
4.10
1.05
3.08
Chemical shift (ppm)

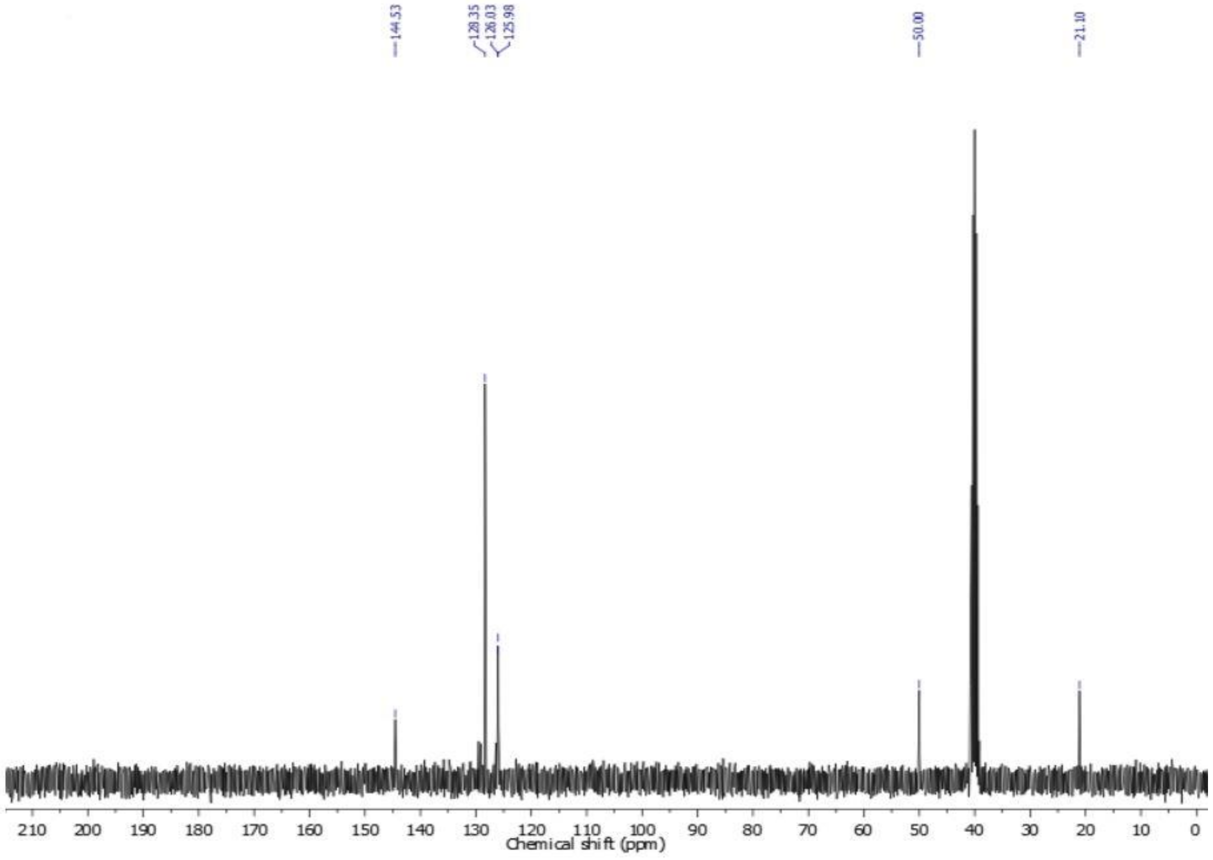
144.53
128.35
126.03
125.98
50.00
21.10
Chemical shift (ppm)

9:

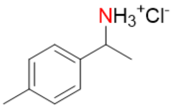
NH3+Cl-

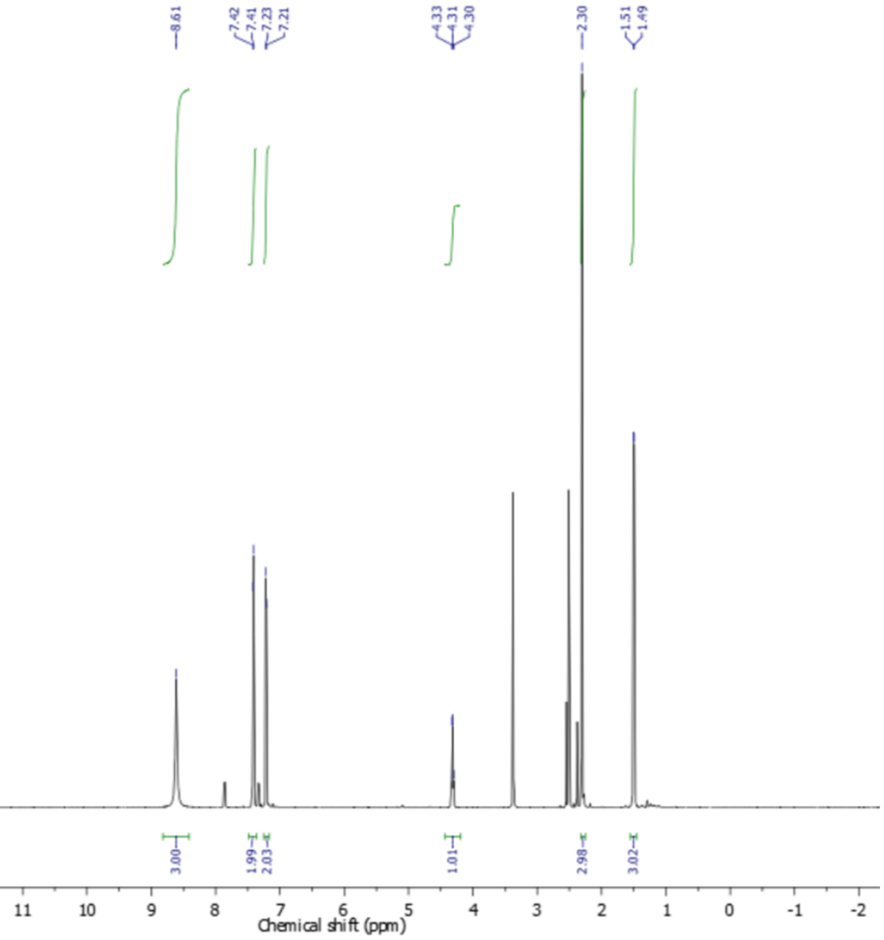
8.61
7.42
7.41
7.23
7.21
4.33
4.31
4.30
2.30
1.51
1.49
3.00
1.99
2.03
1.01
2.98
3.02
Chemical shift (ppm)

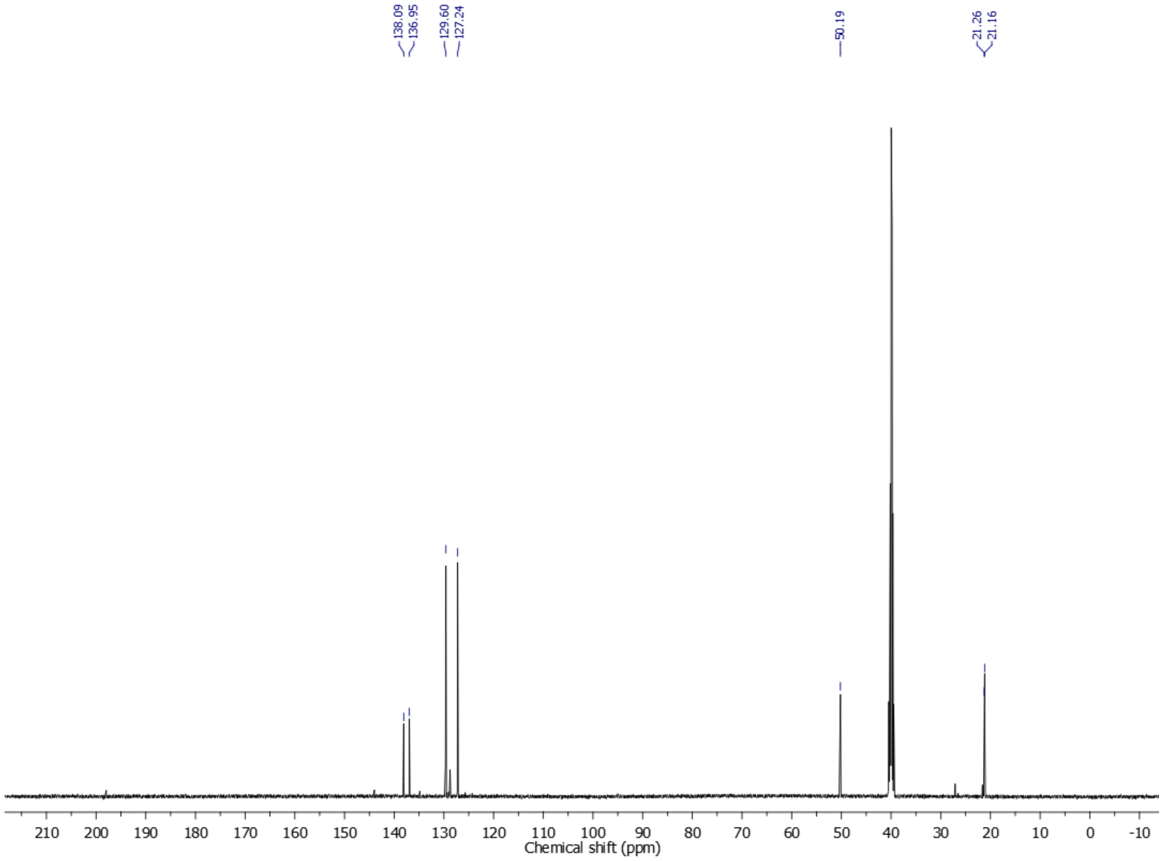
138.09
136.95
129.60
127.24
50.19
21.26
21.16
Chemical shift (ppm)

10:

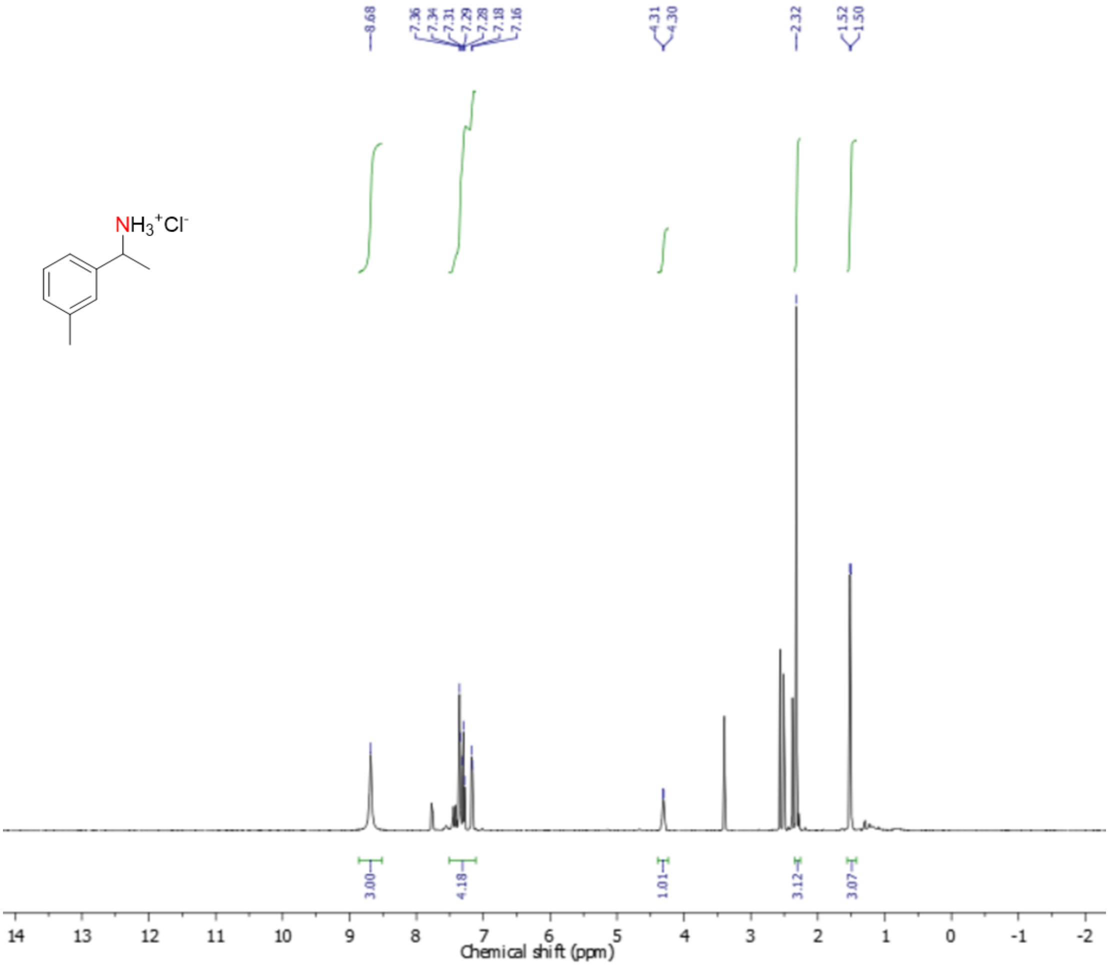
NH3+Cl-
8.68
7.36
7.34
7.31
7.29
7.28
7.18
7.16
4.31
4.30
2.32
1.52
1.50
3.00
4.18
1.01
3.12
3.07
14 13 12 11 10 9 8 7 6 5 4 3 2 1 0 -1 -2
Chemical shift (ppm)

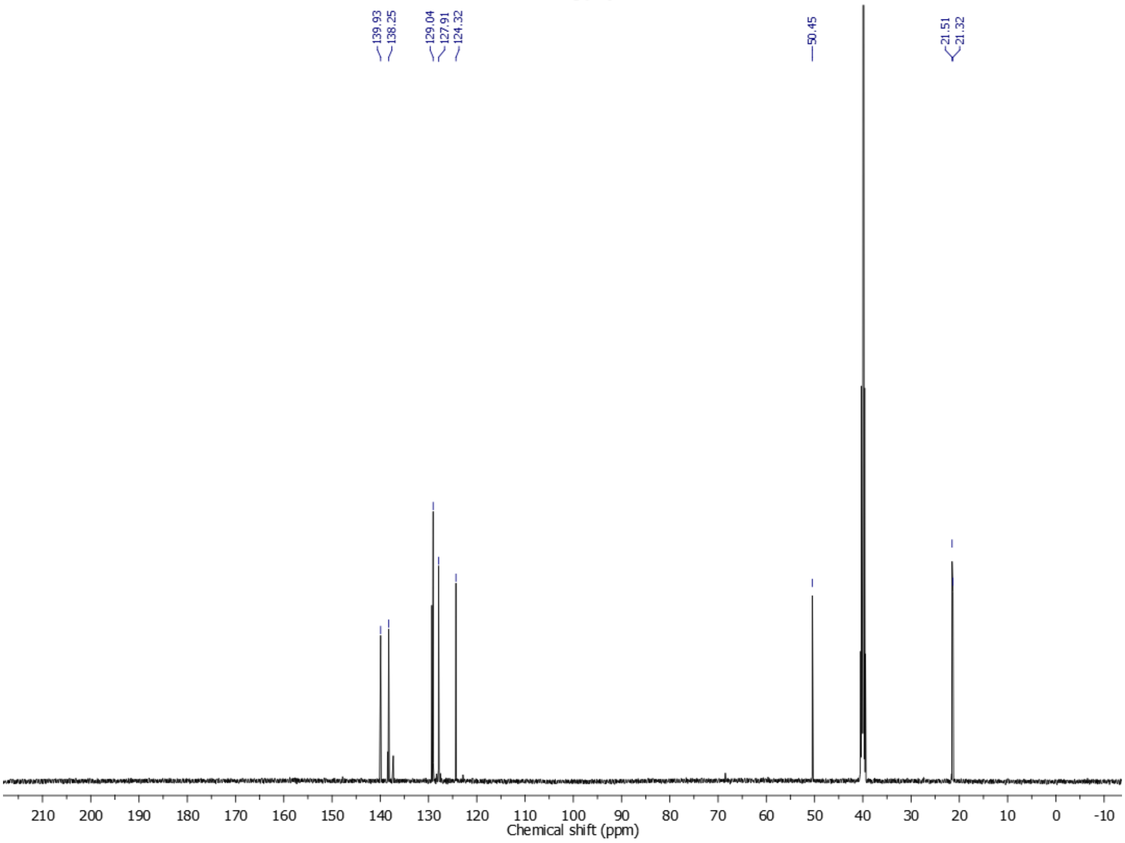
139.93
138.25
129.04
127.91
124.32
50.45
21.51
21.32
210 200 190 180 170 160 150 140 130 120 110 100 90 80 70 60 50 40 30 20 10 0 -10
Chemical shift (ppm)

11:

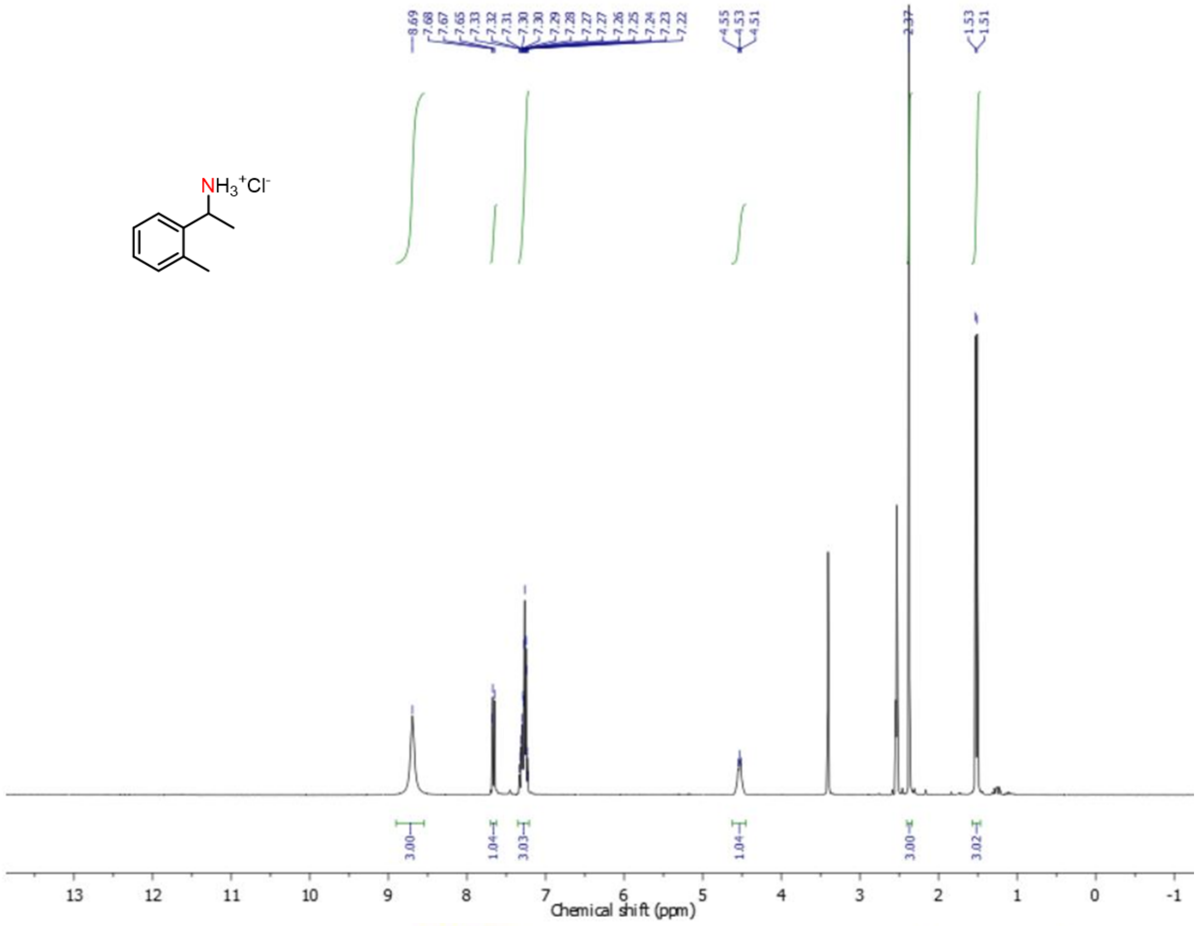

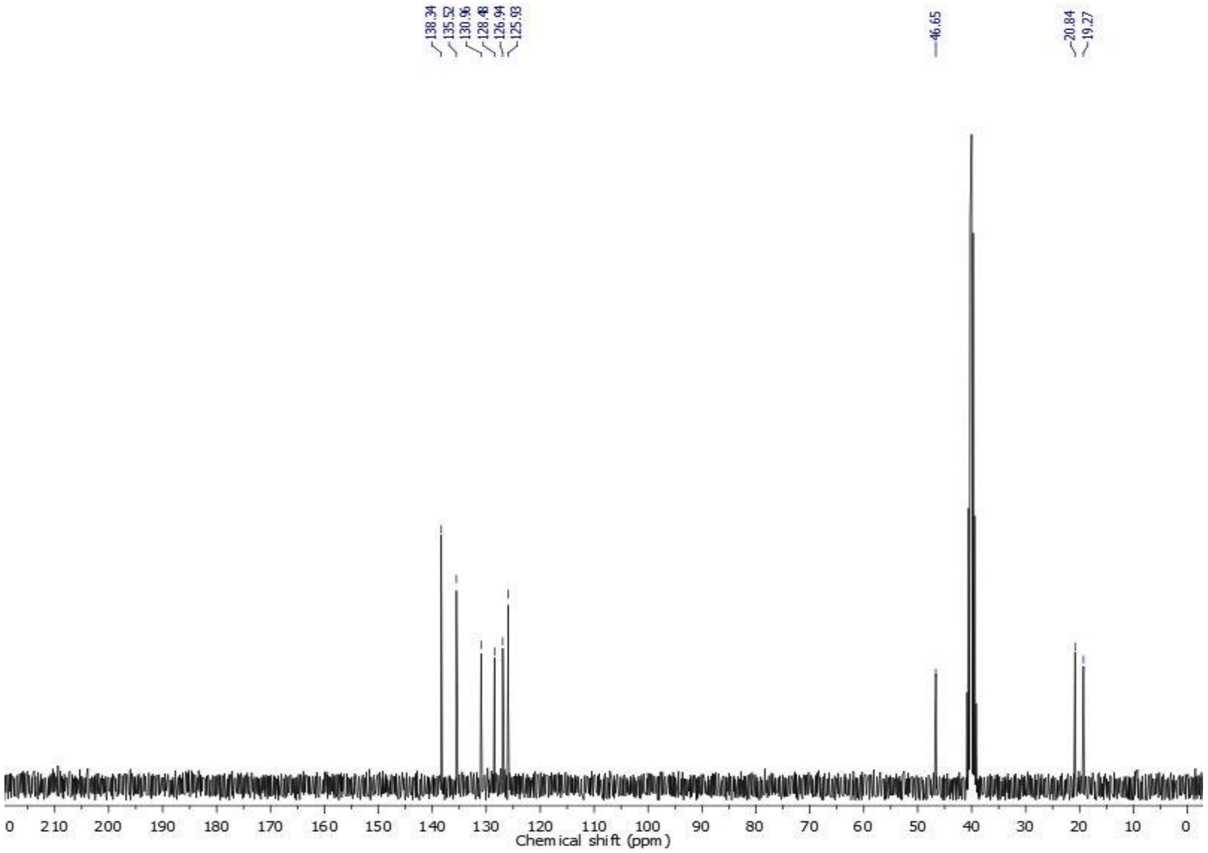

12:

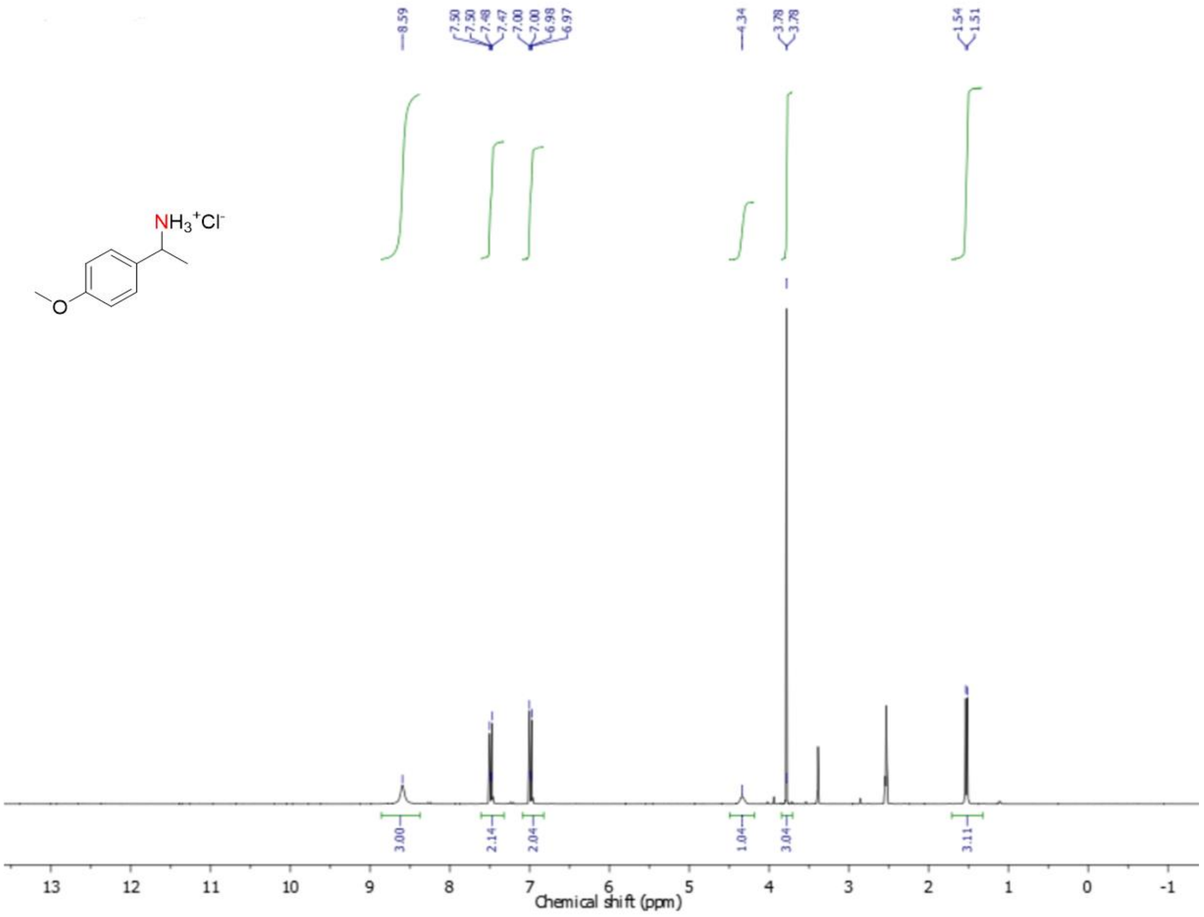
8.59
7.50
7.50
7.48
7.47
7.00
7.00
6.98
6.97
4.34
3.78
3.78
1.54
1.51
NH3+Cl-
O
3.00
2.14
2.04
1.04
3.04
3.11
Chemical shift (ppm)

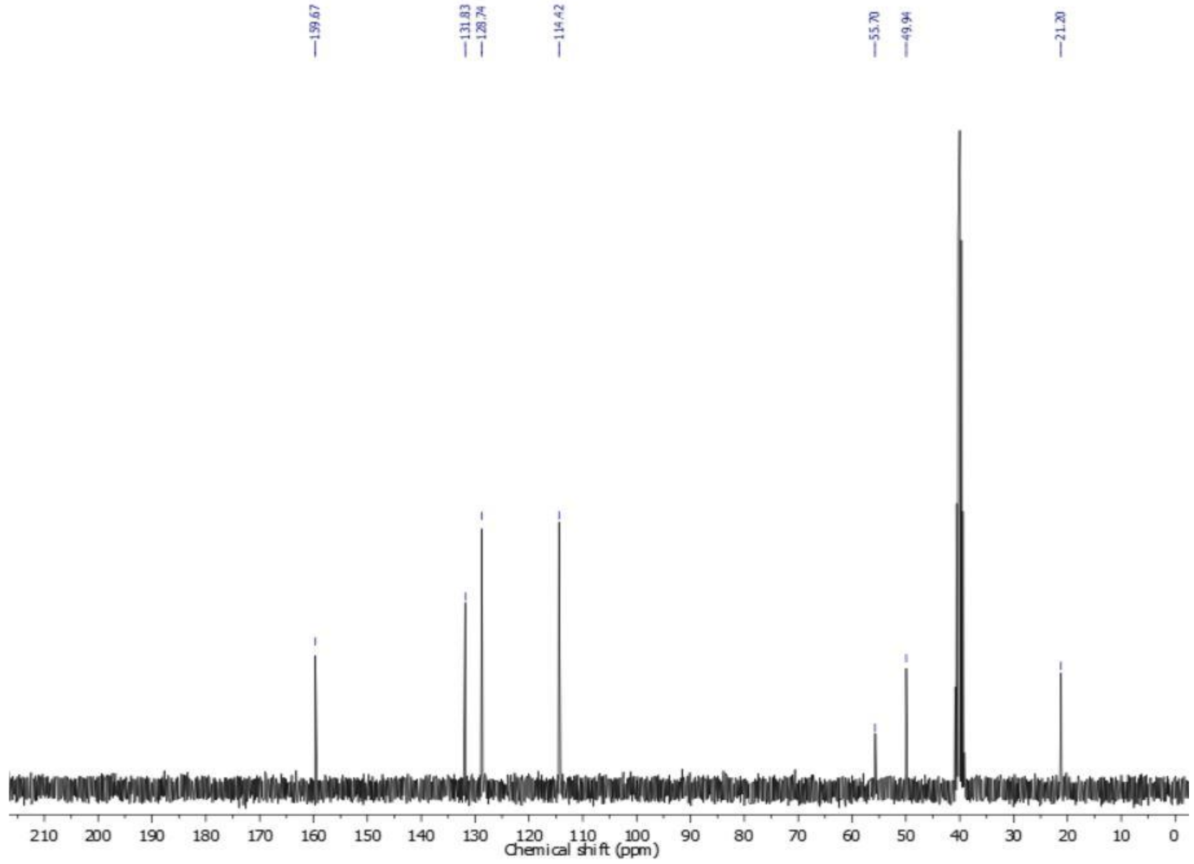
159.67
131.83
128.74
114.42
55.70
49.94
21.20
Chemical shift (ppm)

13:

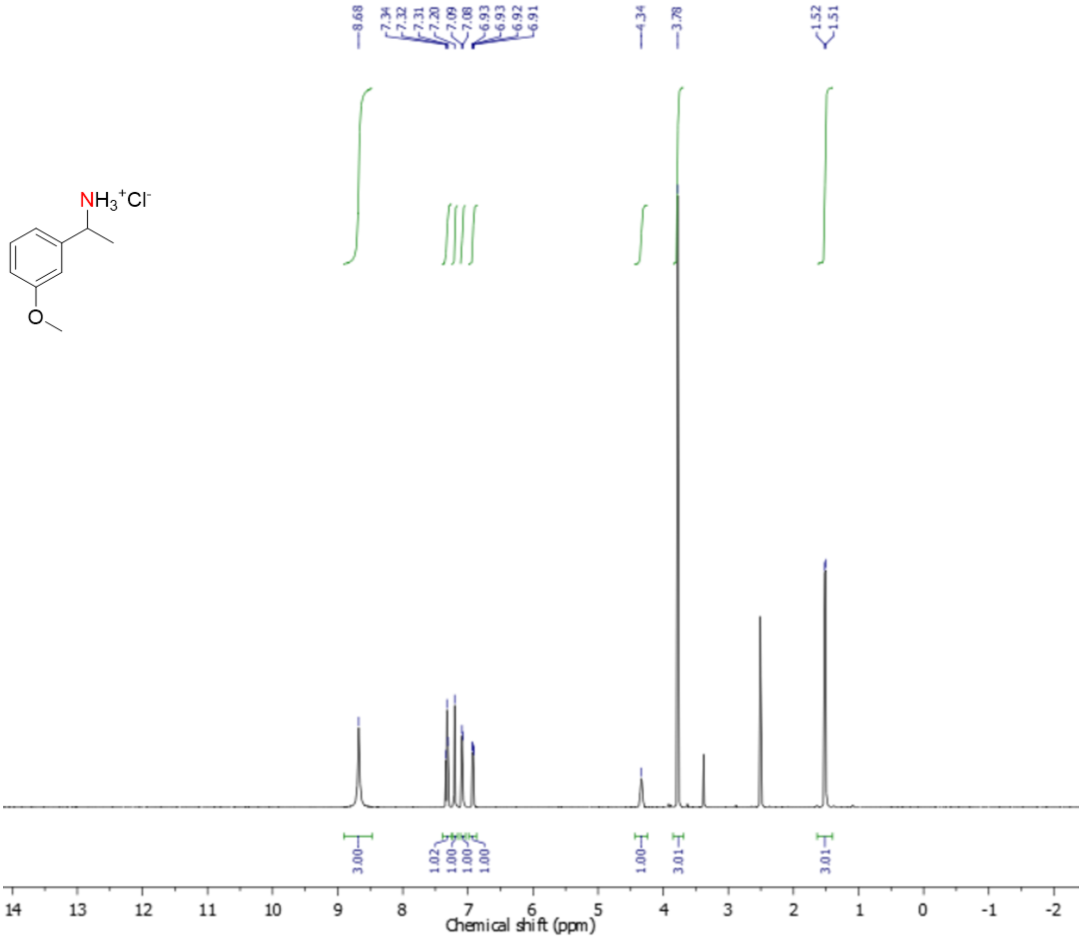
NH3+Cl-
O
8.68
7.34
7.32
7.31
7.20
7.09
7.08
6.93
6.93
6.92
6.91
4.34
3.78
1.52
1.51
3.00
1.02
1.00
1.00
1.00
1.00
3.01
3.01
14
13
12
11
10
9
8
7
6
5
4
3
2
1
0
-1
-2
Chemical shift (ppm)

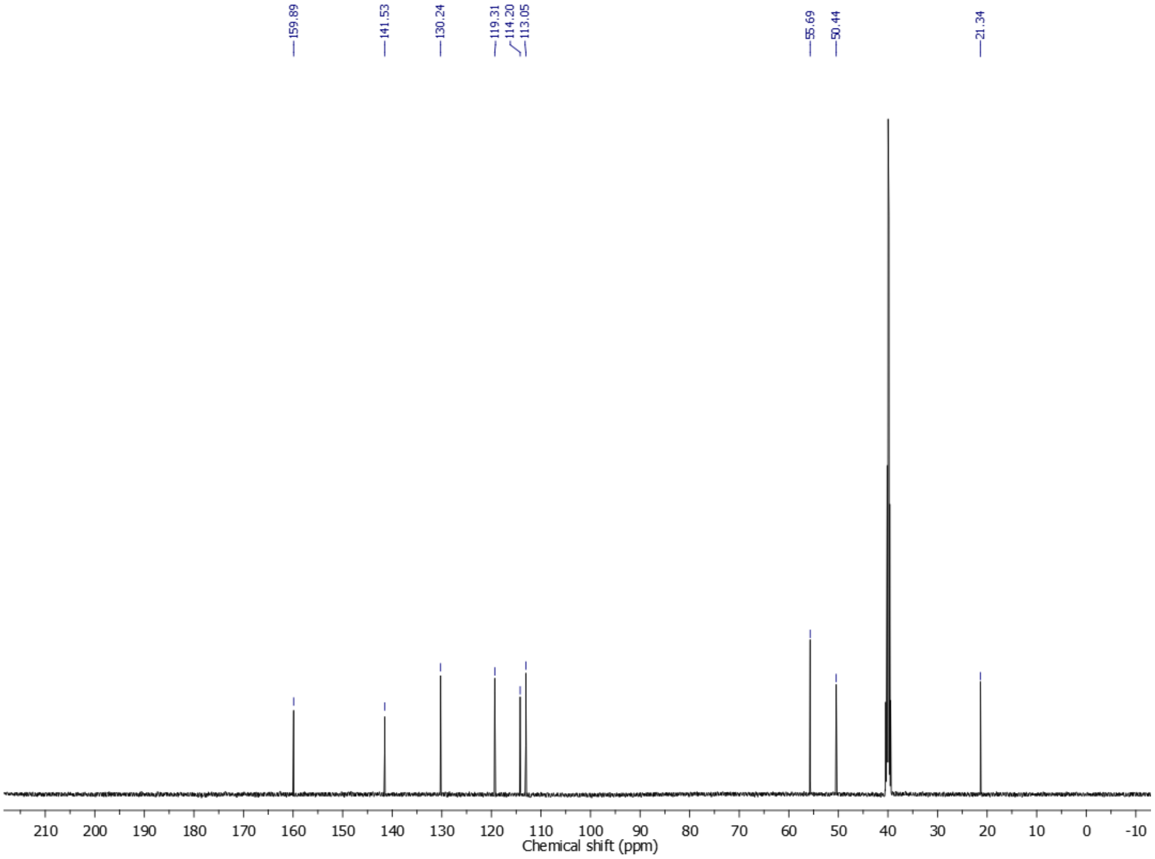
159.89
141.53
130.24
119.31
114.20
113.05
55.69
50.44
21.34
210
200
190
180
170
160
150
140
130
120
110
100
90
80
70
60
50
40
30
20
10
0
-10
Chemical shift (ppm)

14:

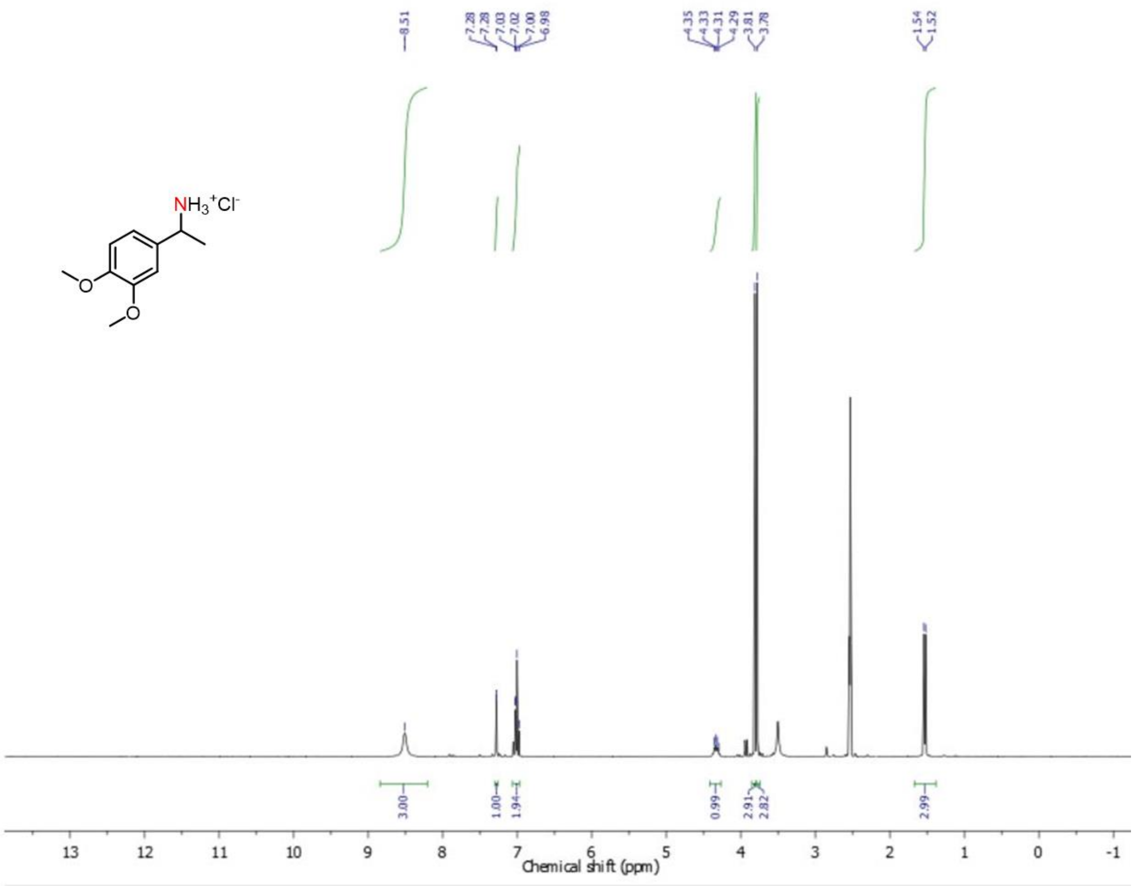
8.51
7.28
7.28
7.03
7.02
7.00
6.98
4.35
4.33
4.31
4.29
3.81
3.78
1.54
1.52
NH3+Cl-
O
O
3.00
1.00
1.94
0.99
2.91
2.82
2.99
13 12 11 10 9 8 7 6 5 4 3 2 1 0 -1
Chemical shift (ppm)

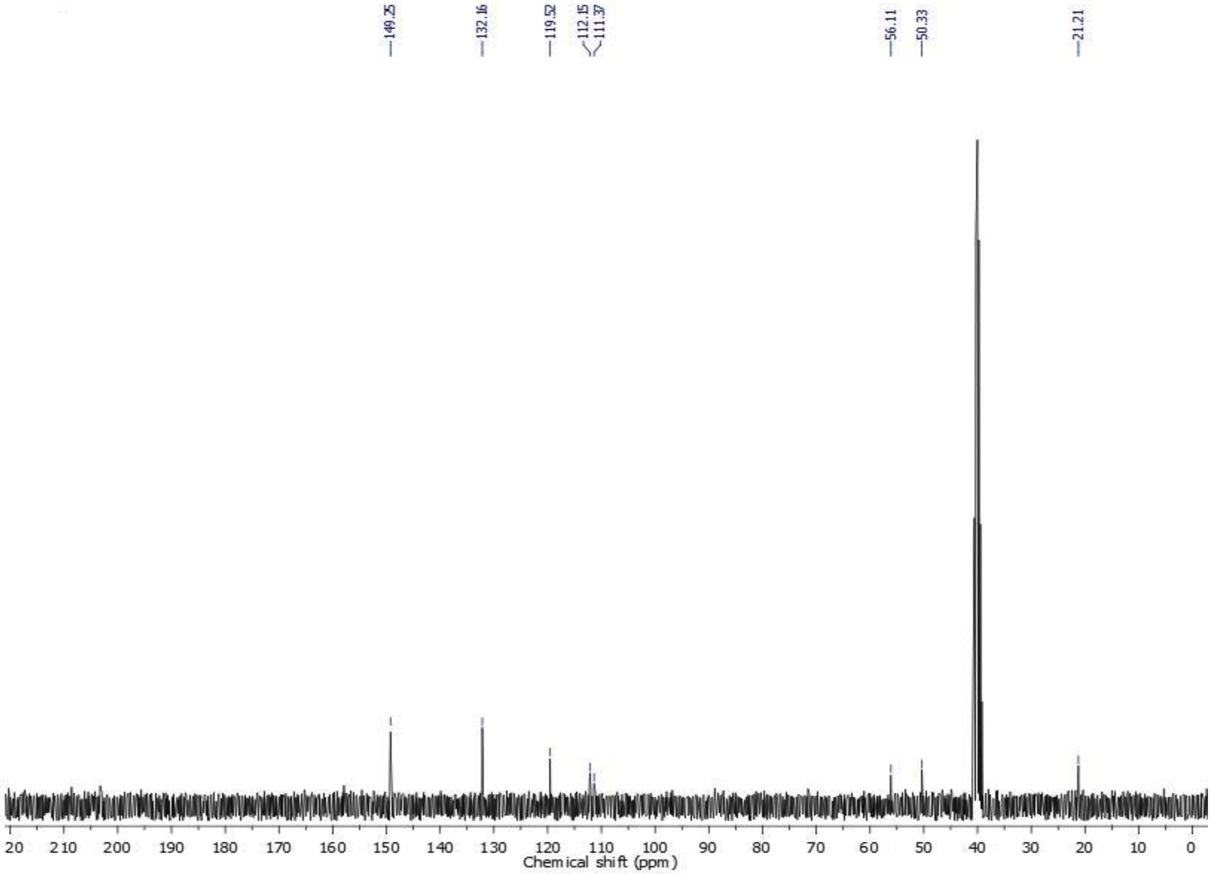
149.25
132.16
119.52
112.15
111.37
56.11
50.33
21.21
20 210 200 190 180 170 160 150 140 130 120 110 100 90 80 70 60 50 40 30 20 10 0
Chemical shift (ppm)

15:

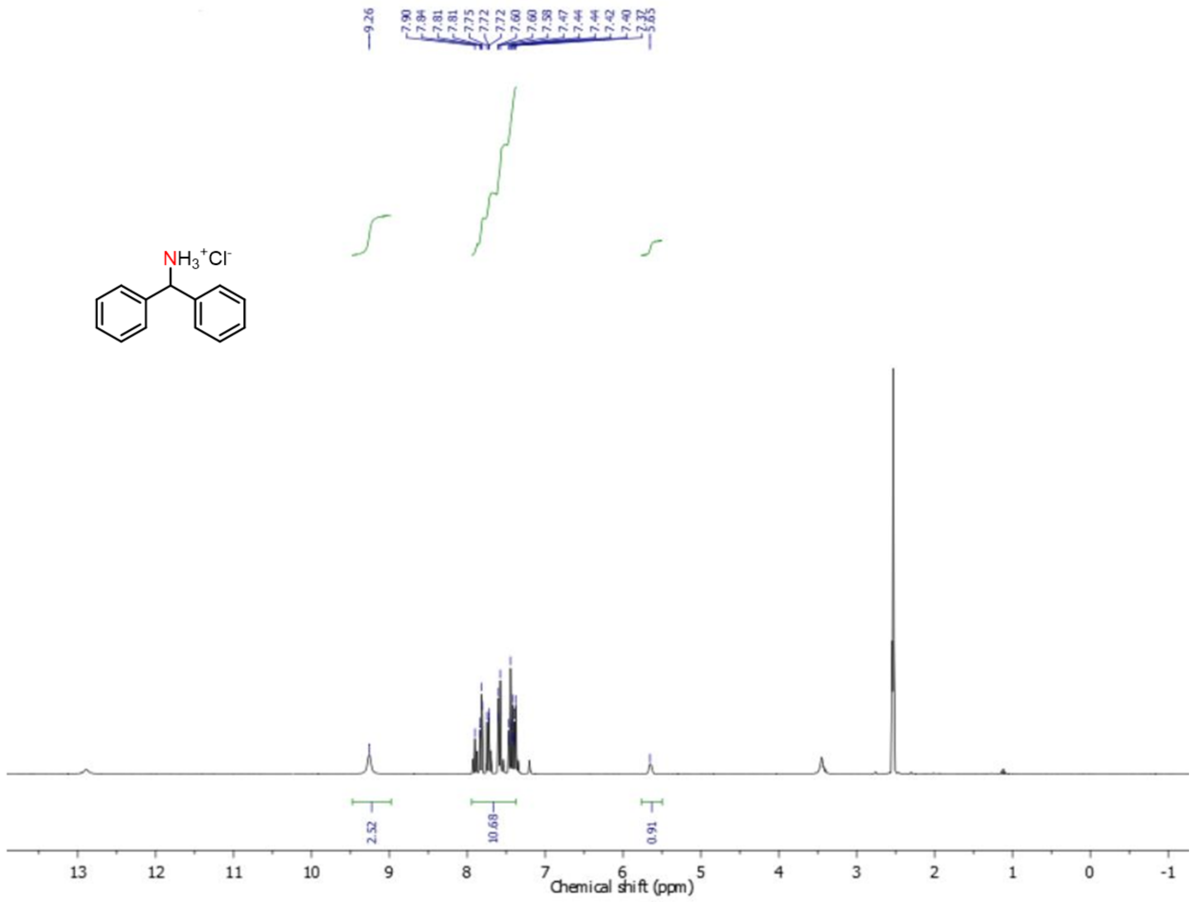
NH3+Cl-
2.52
10.68
0.91
Chemical shift (ppm)

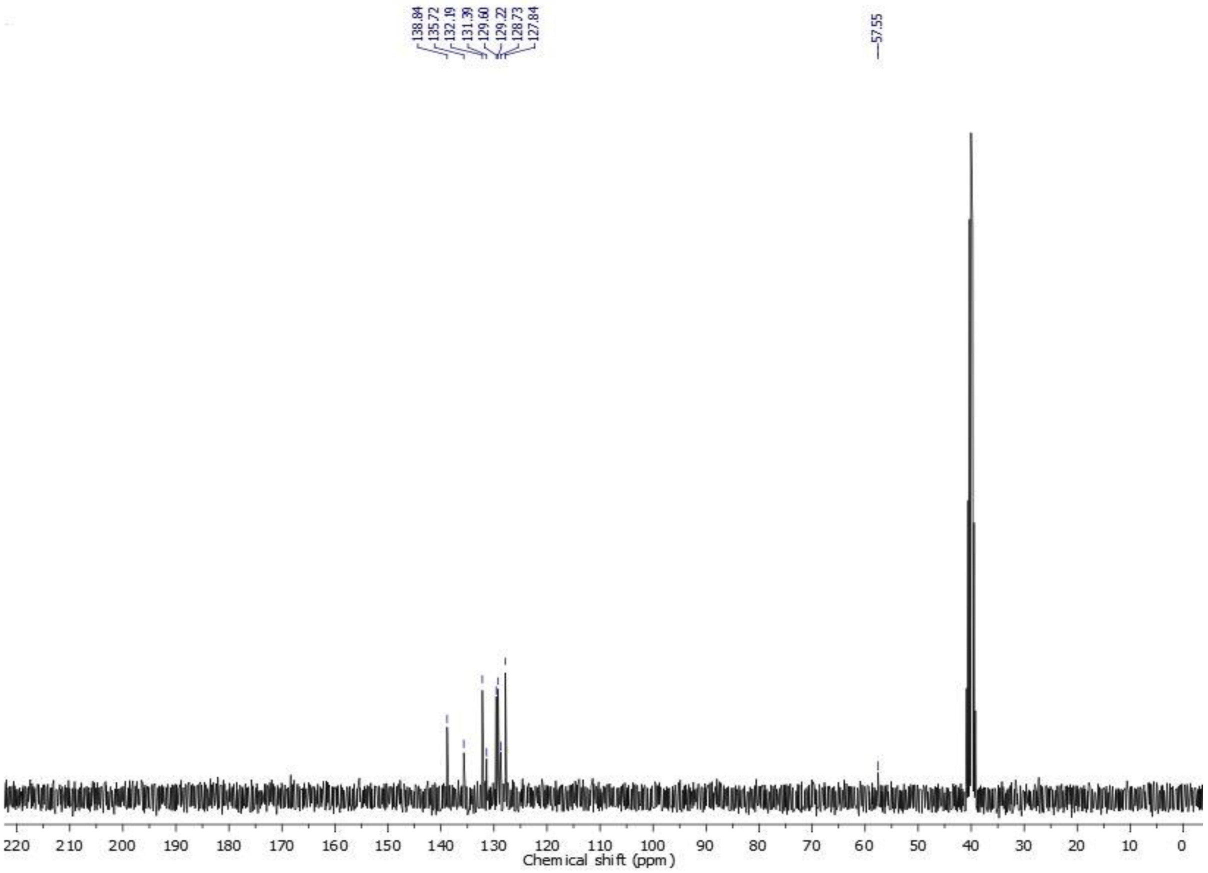
138.84
135.72
132.19
131.39
129.60
129.22
128.73
127.84
57.55
Chemical shift (ppm)

16:

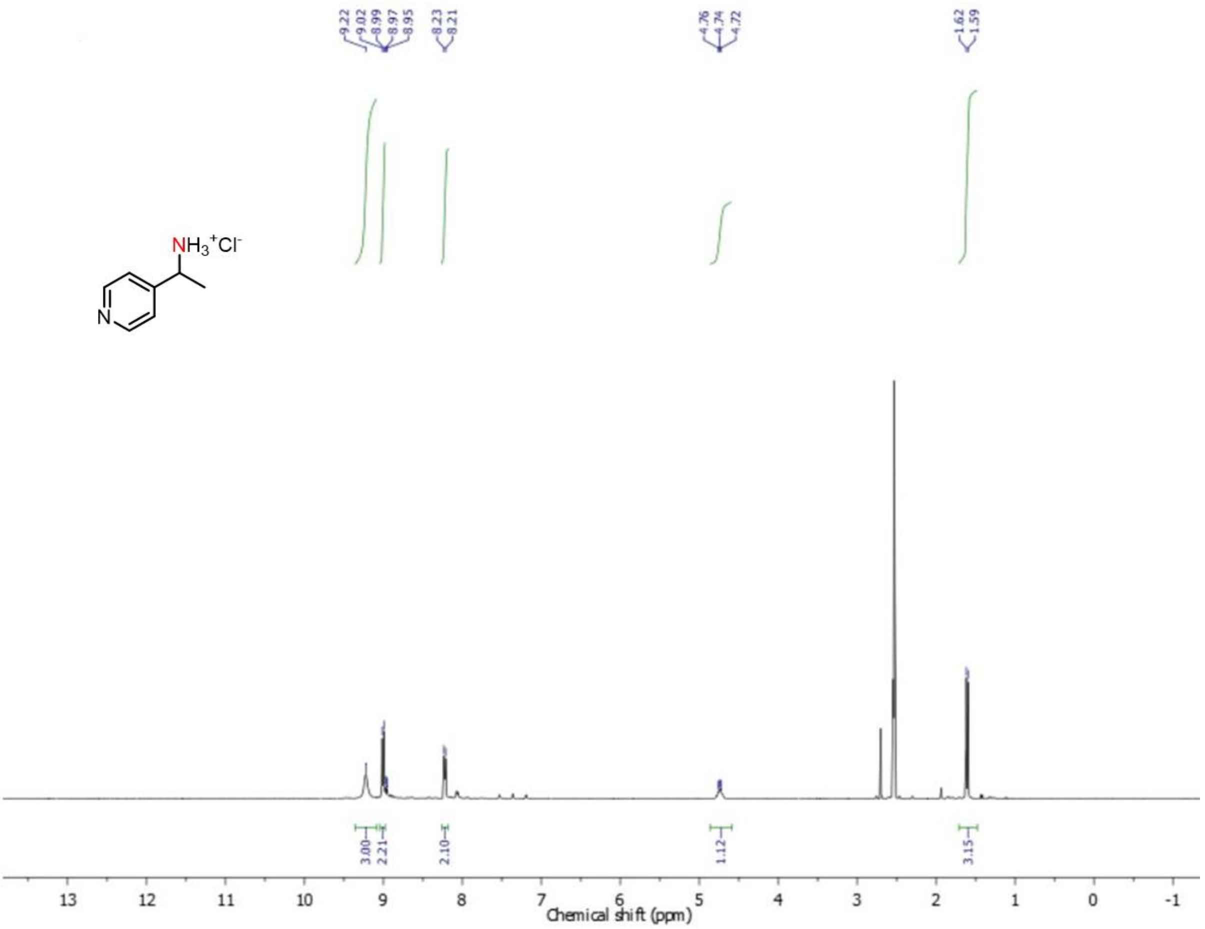
9.22
9.02
8.99
8.97
8.95
8.23
8.21
4.76
4.74
4.72
1.62
1.59
NH3+Cl-
N
3.00
2.21
2.10
1.12
3.15
Chemical shift (ppm)

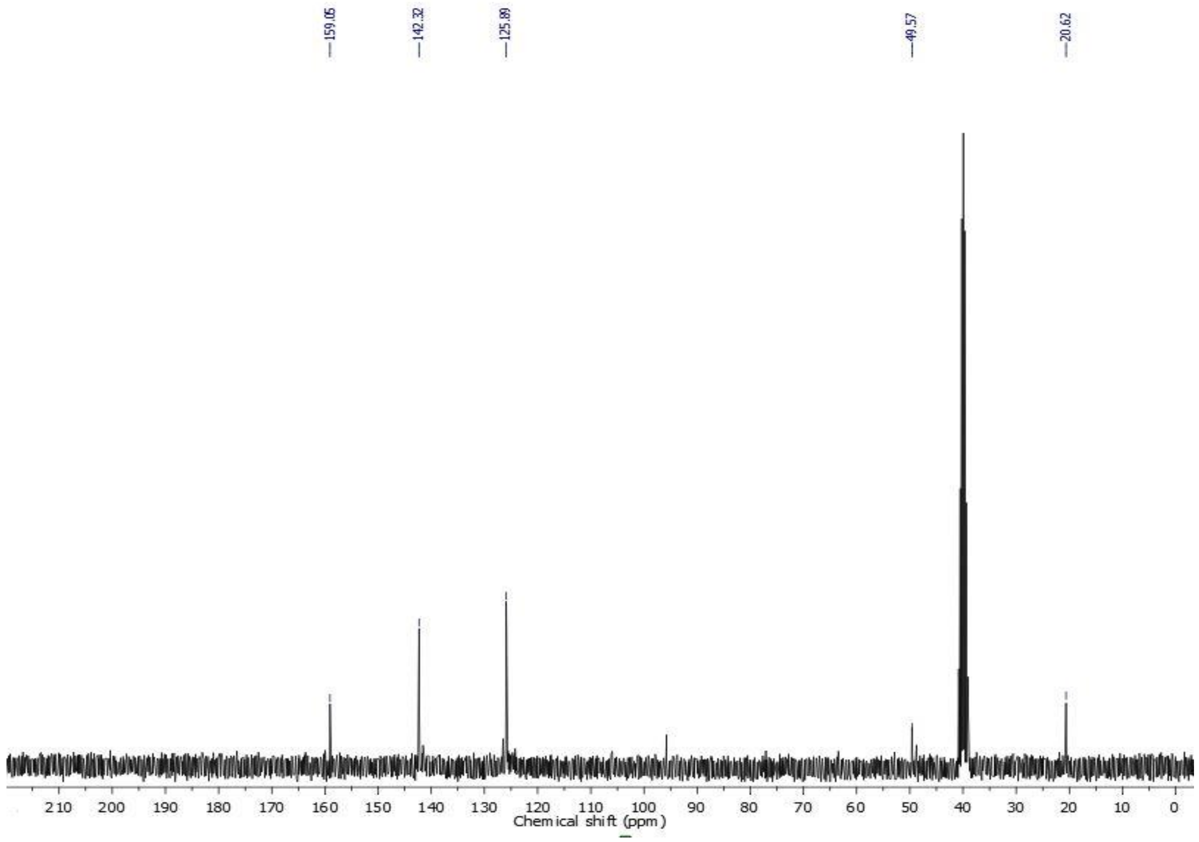
159.05
142.32
125.89
49.57
20.62
Chemical shift (ppm)

17:

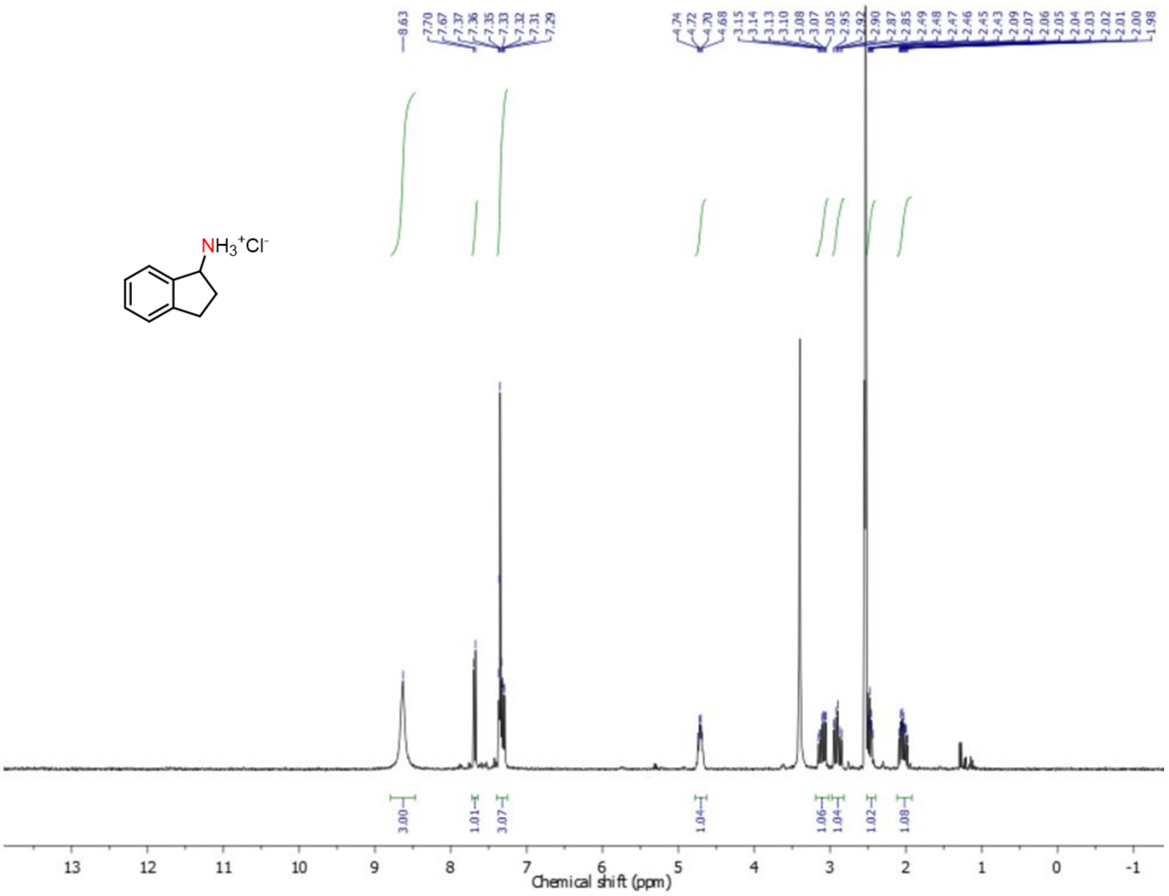

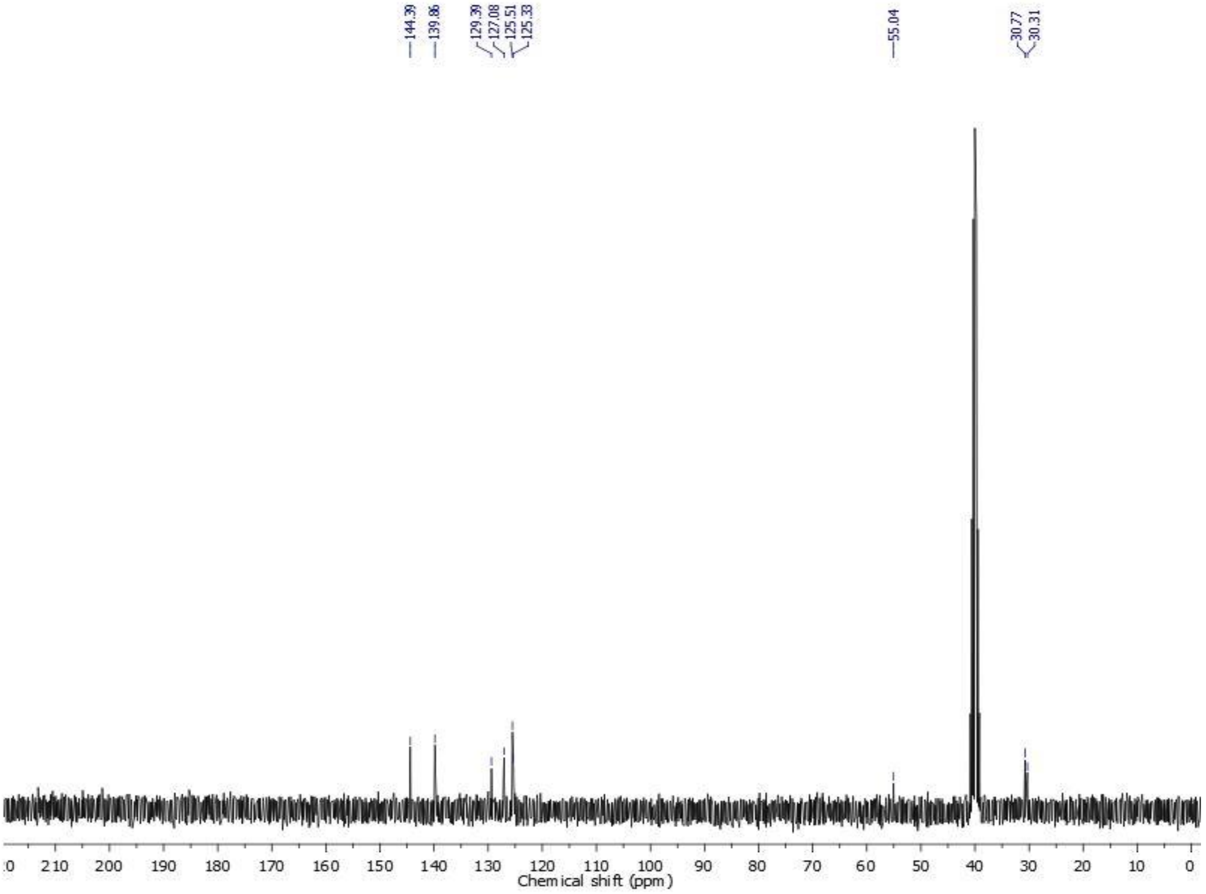

18:

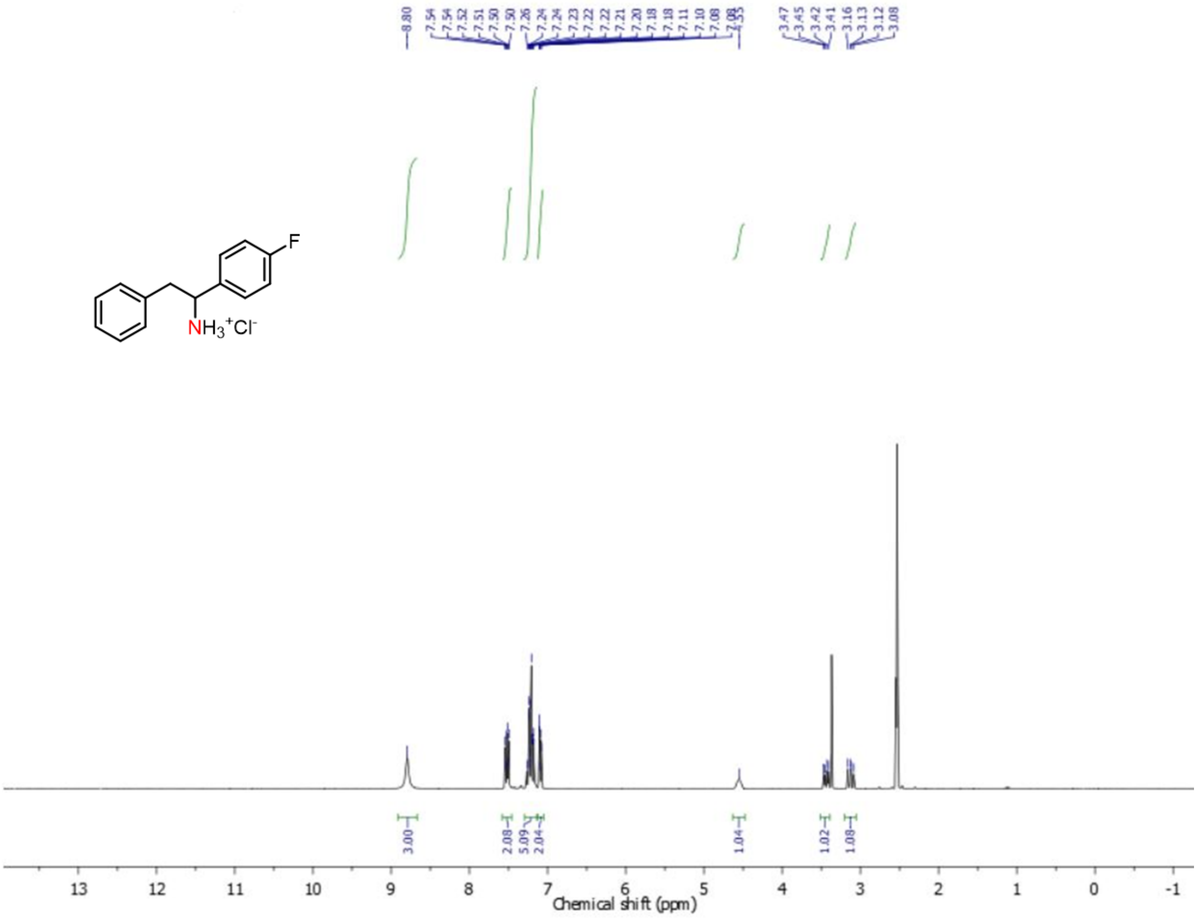

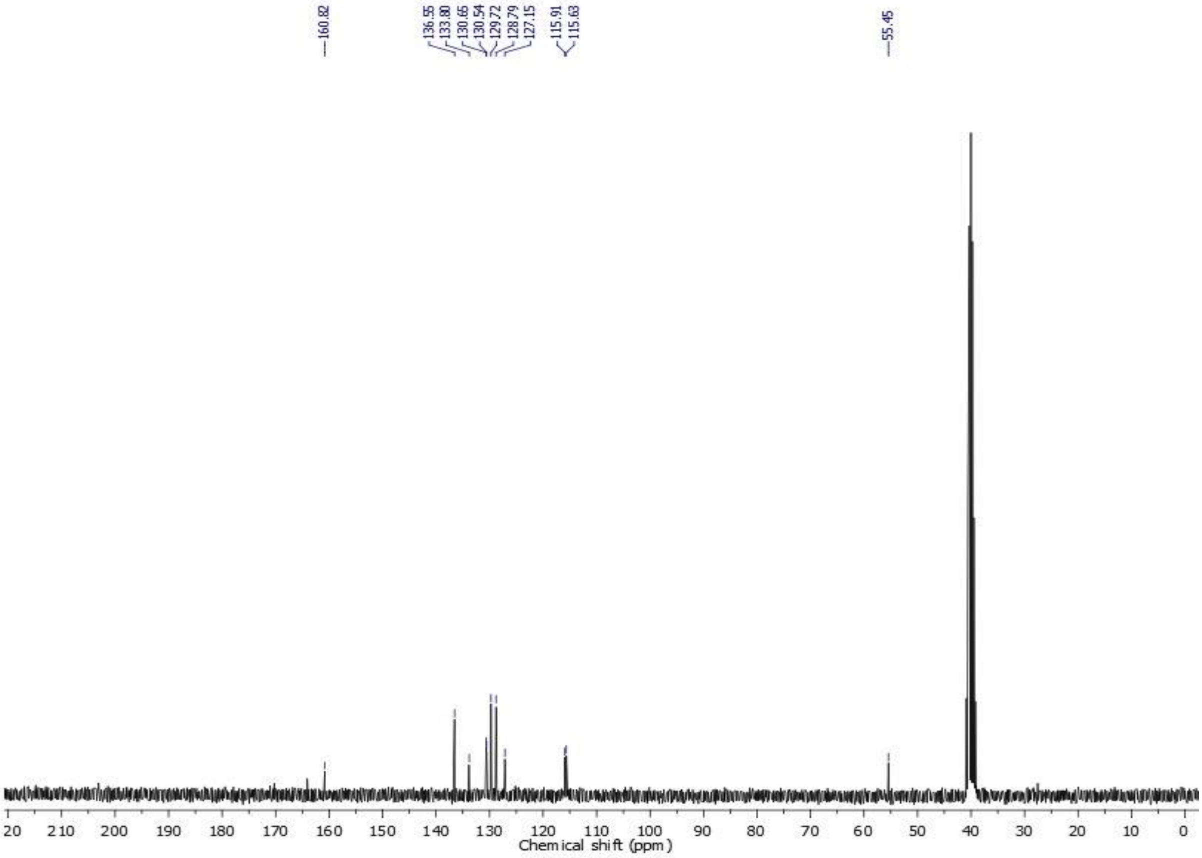

19:

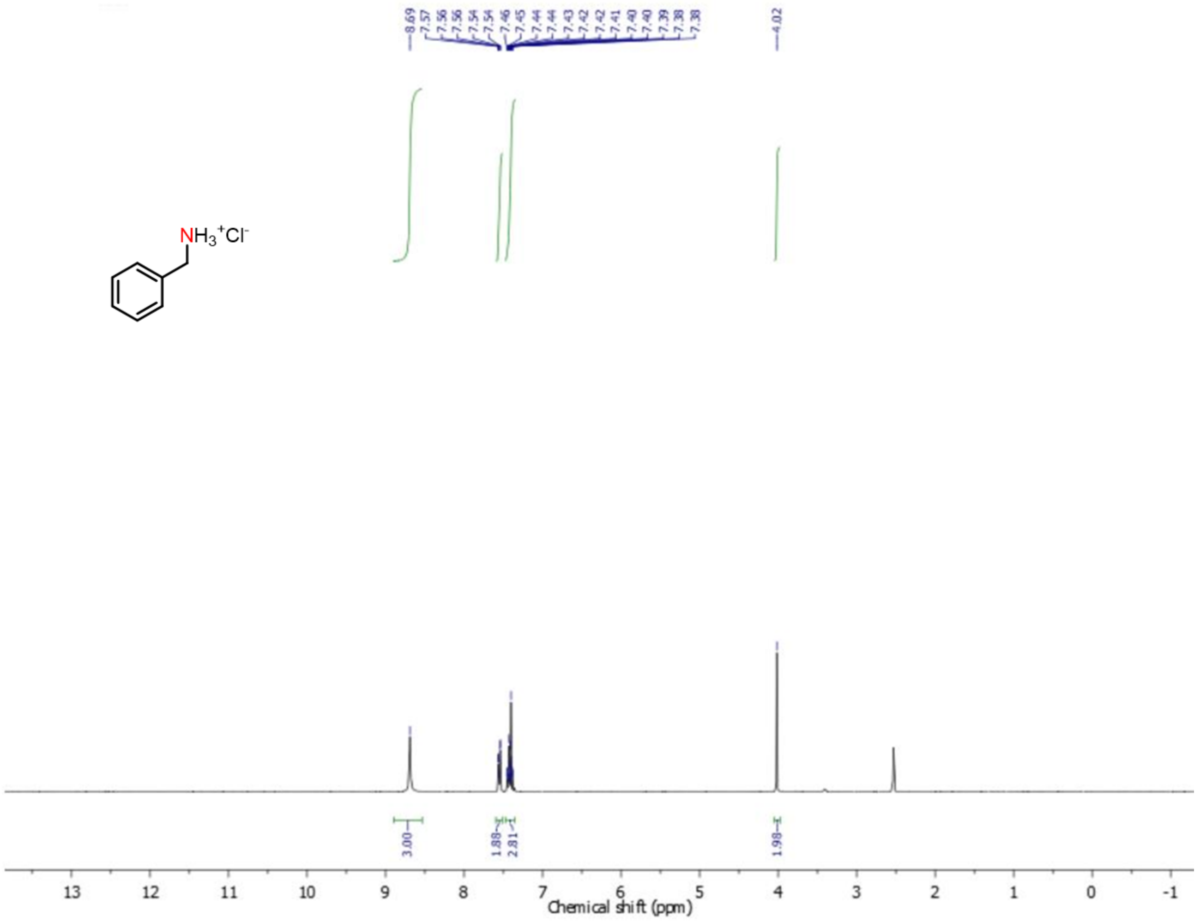
NH3+Cl-
8.69
7.57
7.56
7.56
7.54
7.54
7.46
7.45
7.44
7.44
7.43
7.42
7.42
7.41
7.40
7.40
7.39
7.38
7.38
4.02
3.00
1.88
2.81
1.98
Chemical shift (ppm)

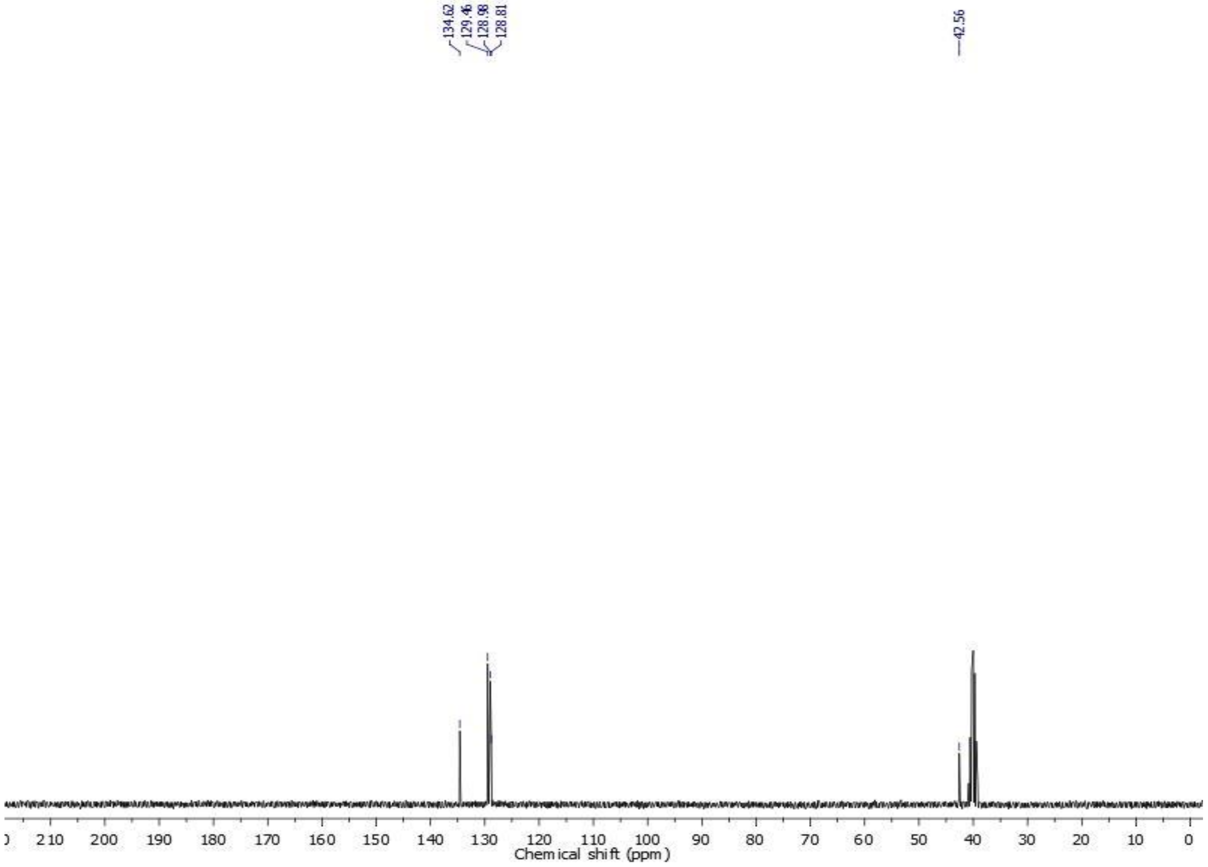
134.62
129.46
128.98
128.81
42.56
Chemical shift (ppm)

20:

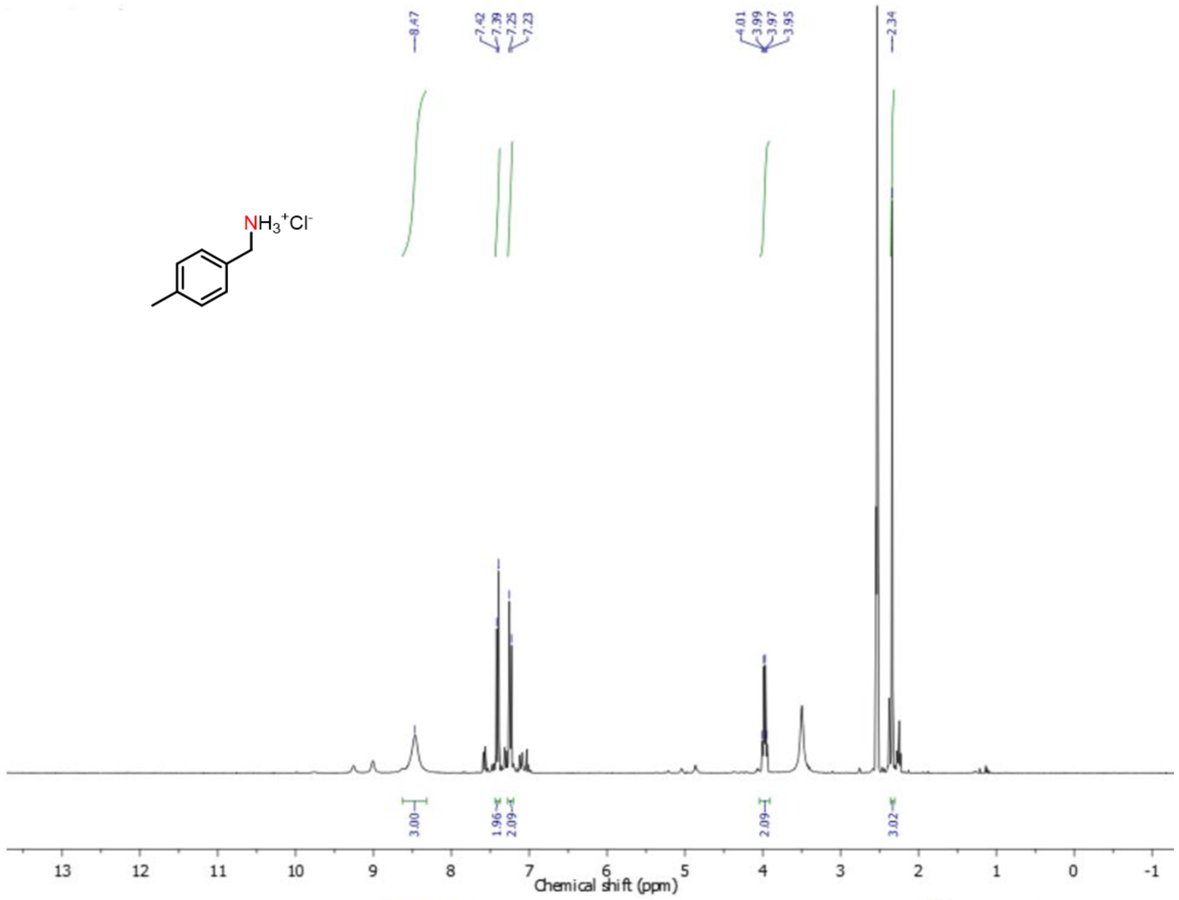
NH3+Cl-
8.47
7.42
7.39
7.25
7.23
4.01
3.99
3.97
3.95
2.34
3.00
1.96
2.09
2.09
3.02
Chemical shift (ppm)

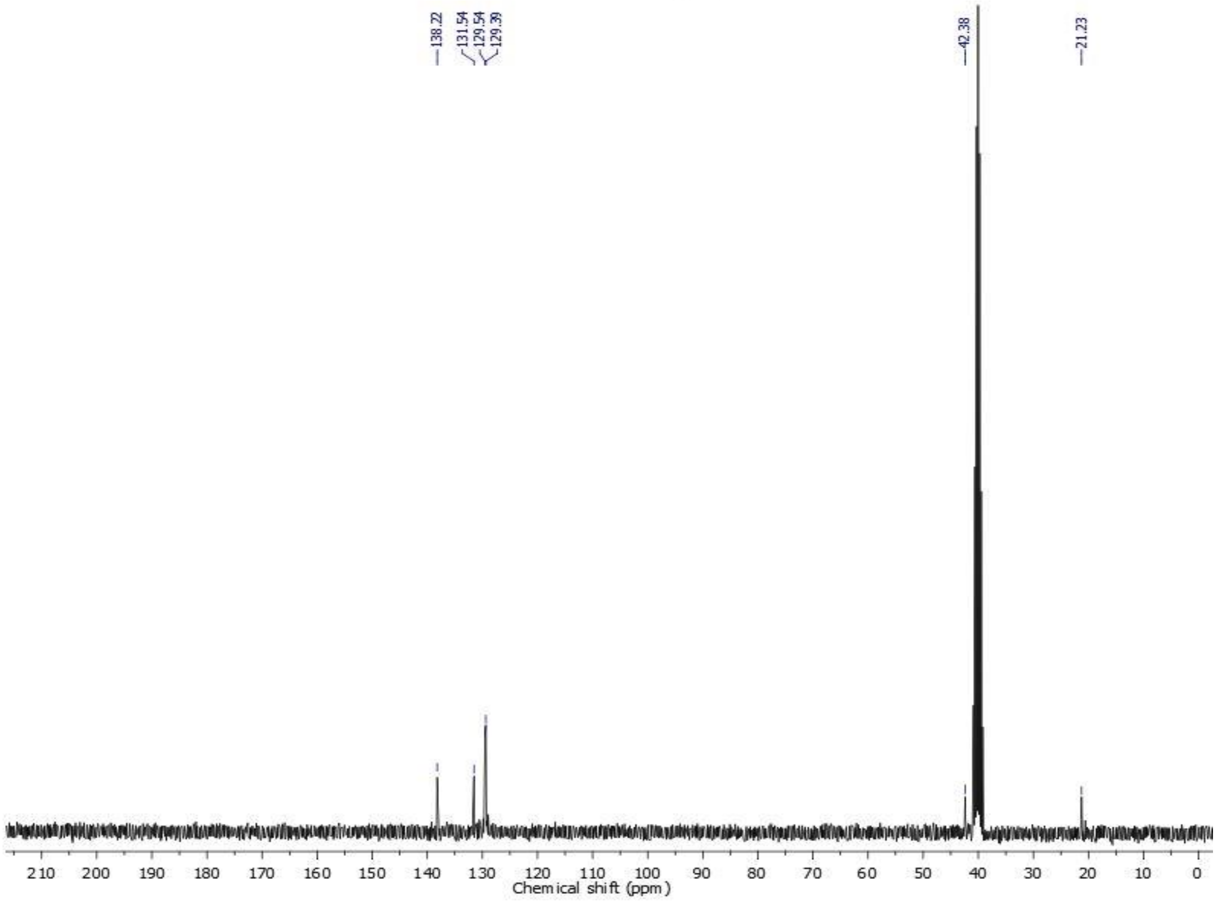
138.22
131.54
129.54
129.39
42.38
21.23
Chemical shift (ppm)

21:

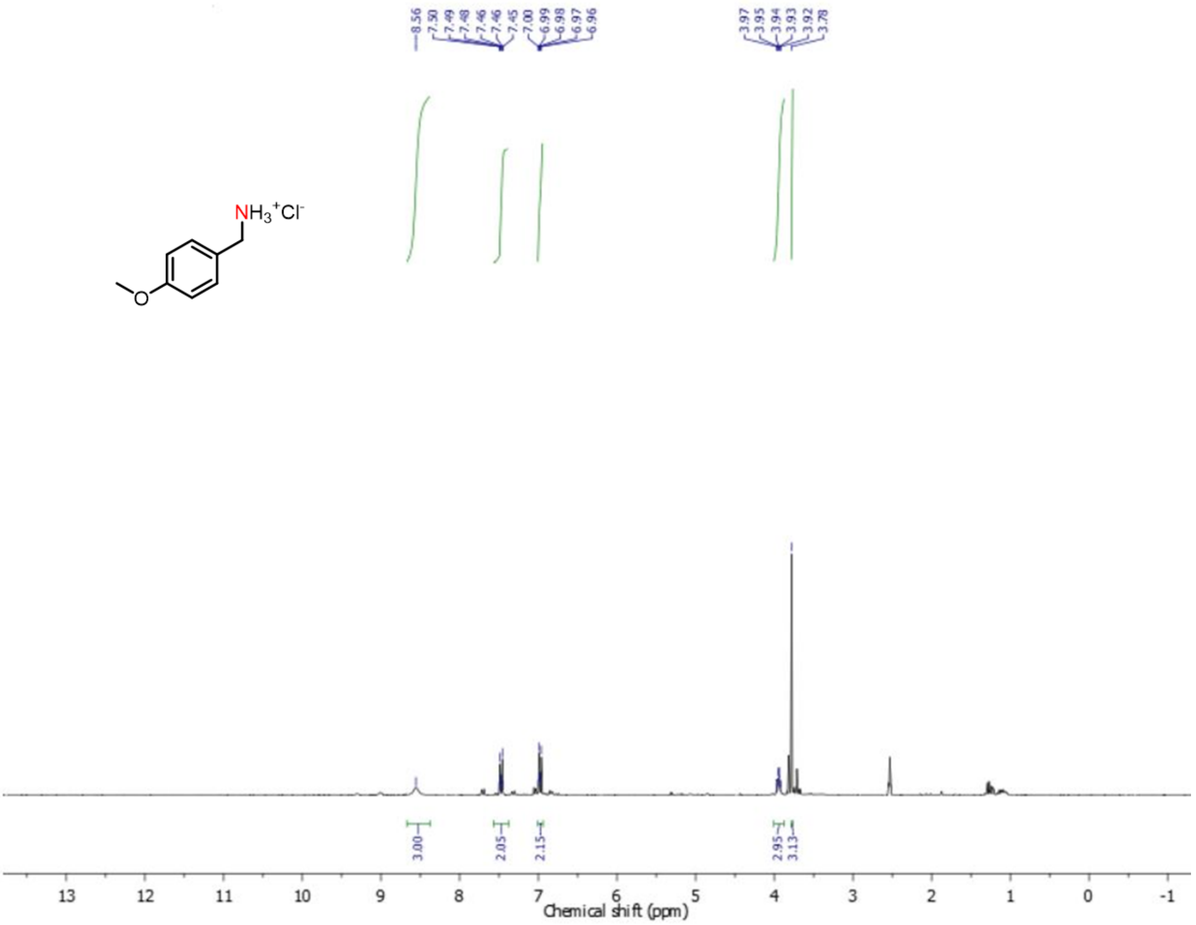
NH3+Cl-
O
8.56
3.97
3.78
3.00
2.05
2.15
2.95
3.13
Chemical shift (ppm)

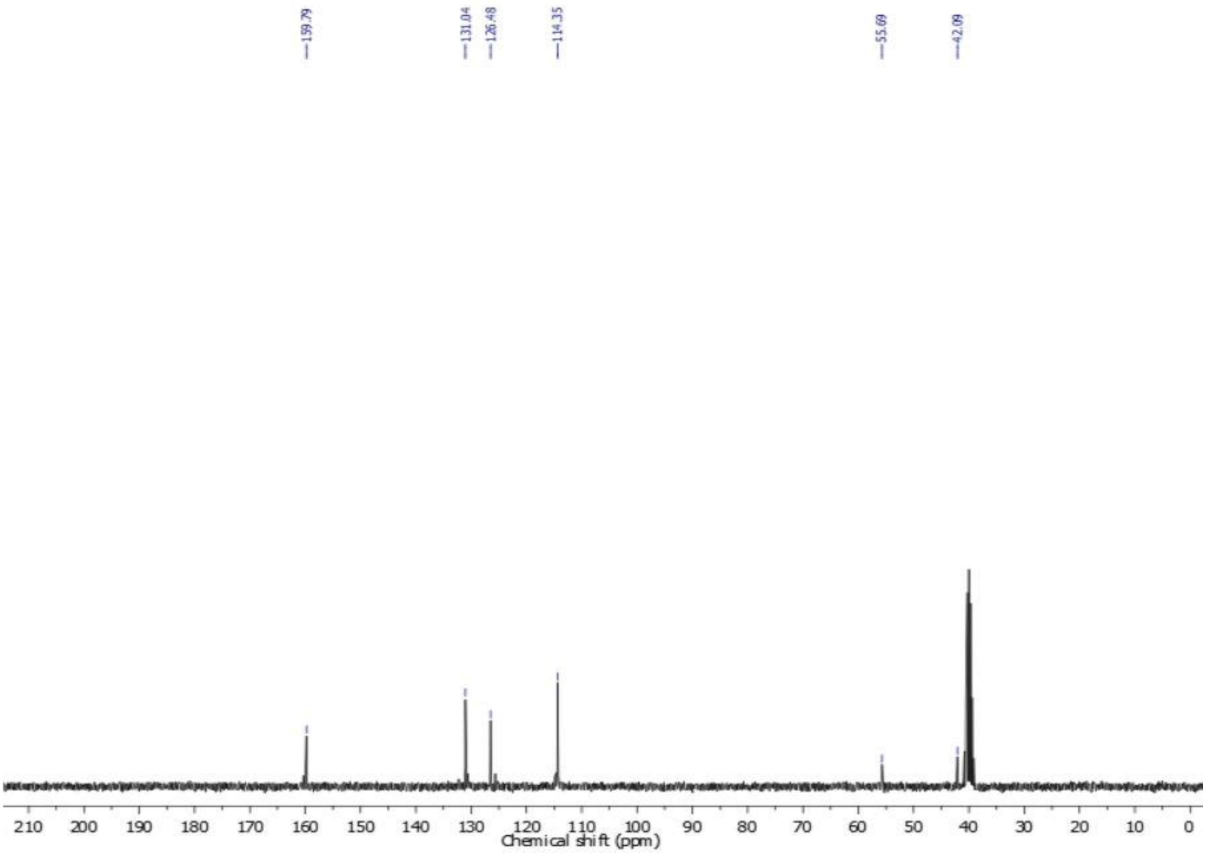
159.79
131.04
126.48
114.35
55.69
42.09
Chemical shift (ppm)

22:

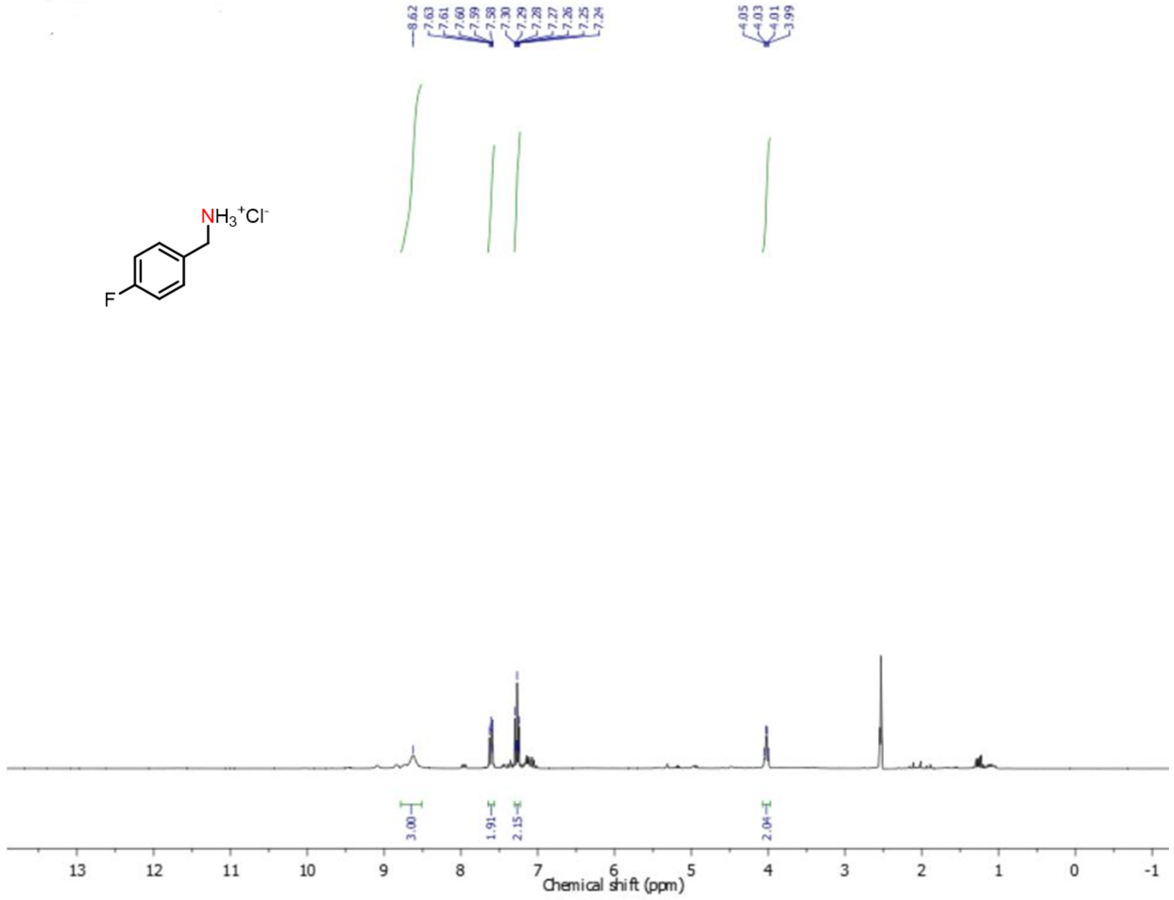
8.62
7.63
7.61
7.60
7.59
7.58
7.30
7.29
7.28
7.27
7.26
7.25
7.24
4.05
4.03
4.01
3.99
NH3+Cl-
F
3.00
1.91
2.15
2.04
Chemical shift (ppm)

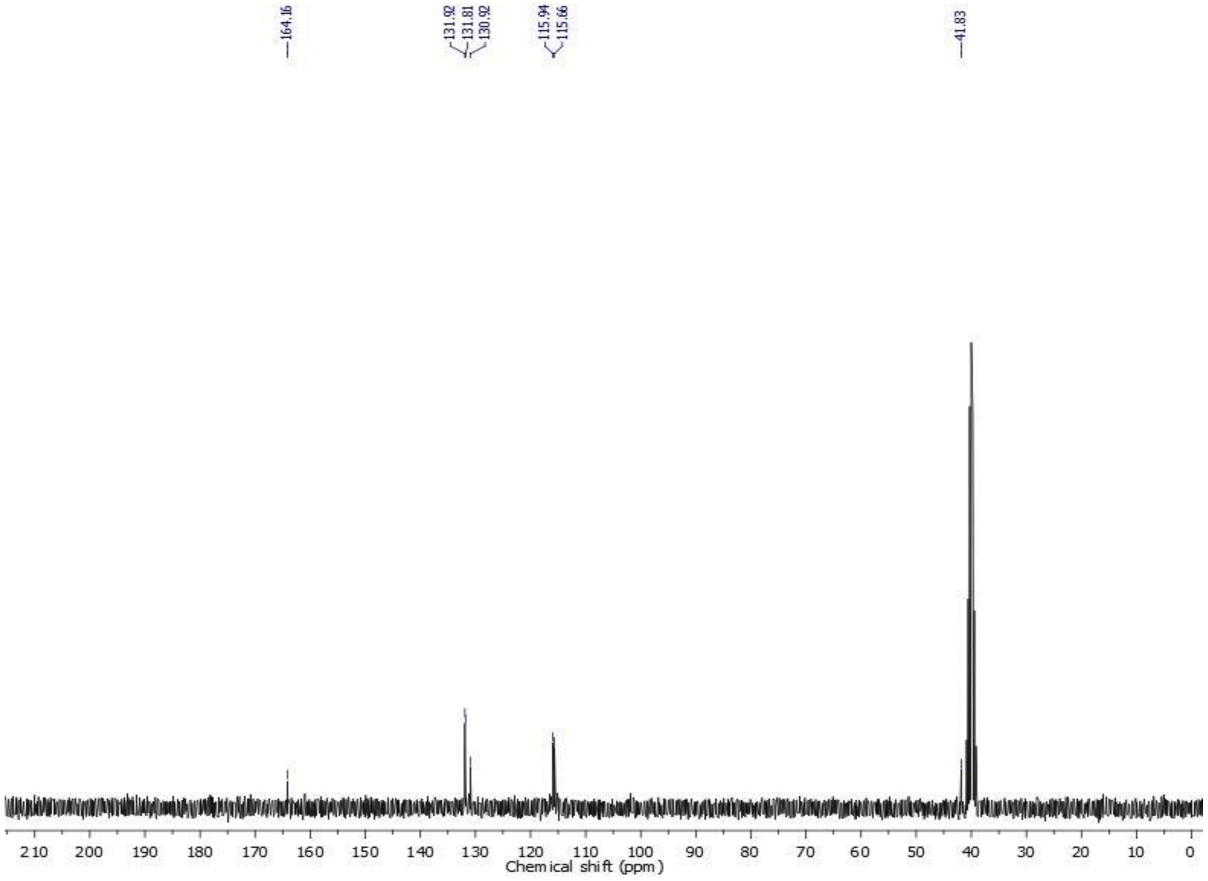
164.16
131.92
131.81
130.92
115.94
115.66
41.83
Chemical shift (ppm)

23:

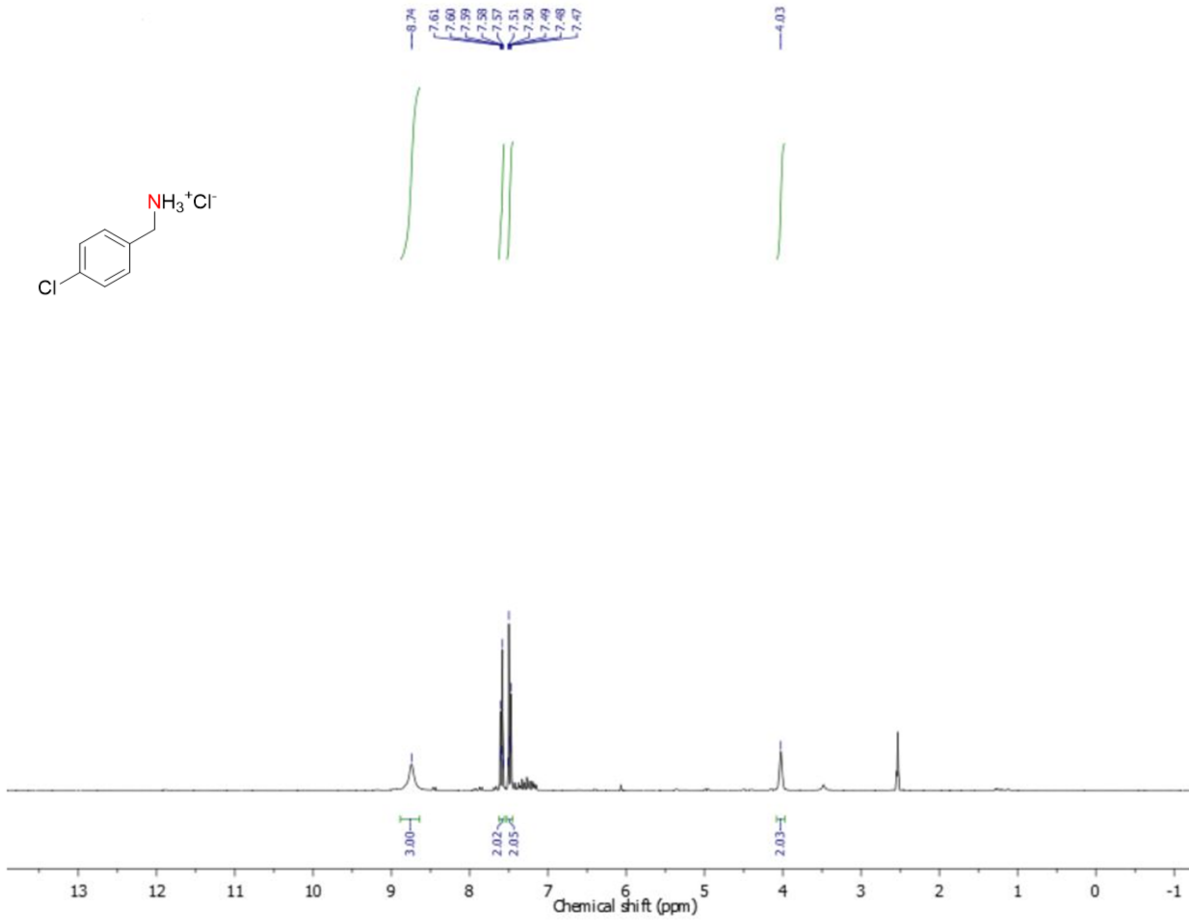
8.74
7.61
7.60
7.59
7.58
7.57
7.51
7.50
7.49
7.48
7.47
4.03
3.00
2.02
2.05
2.03
Chemical shift (ppm)

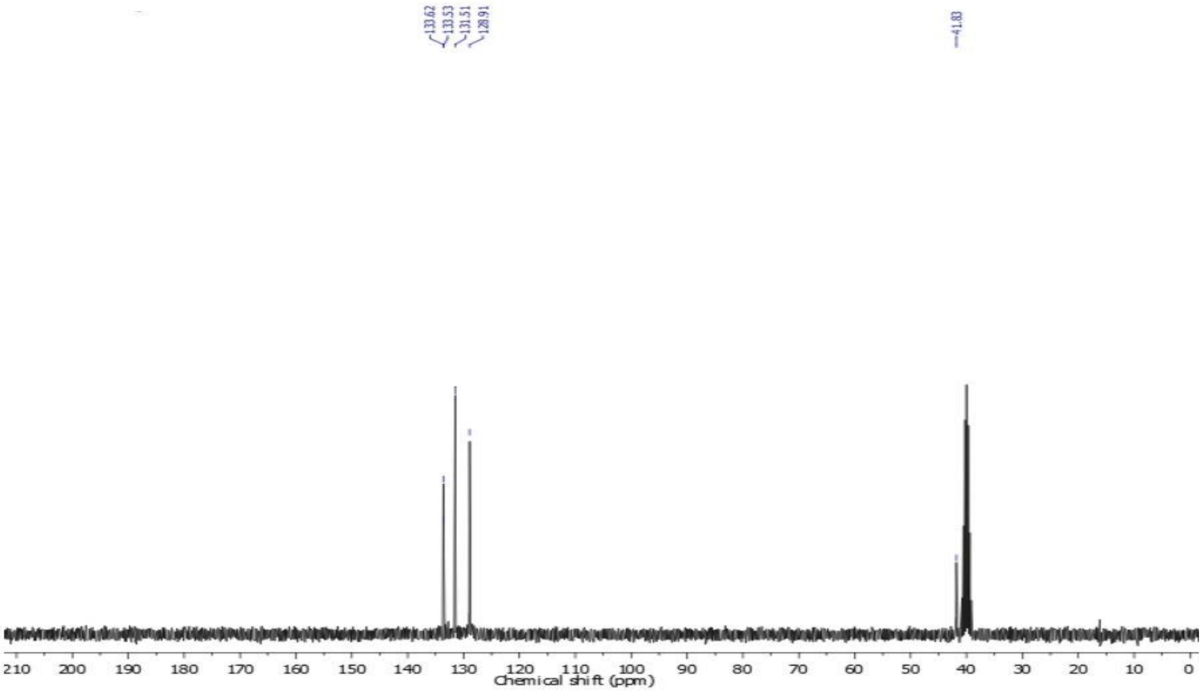
133.62
133.53
131.51
128.91
41.83
Chemical shift (ppm)

24:

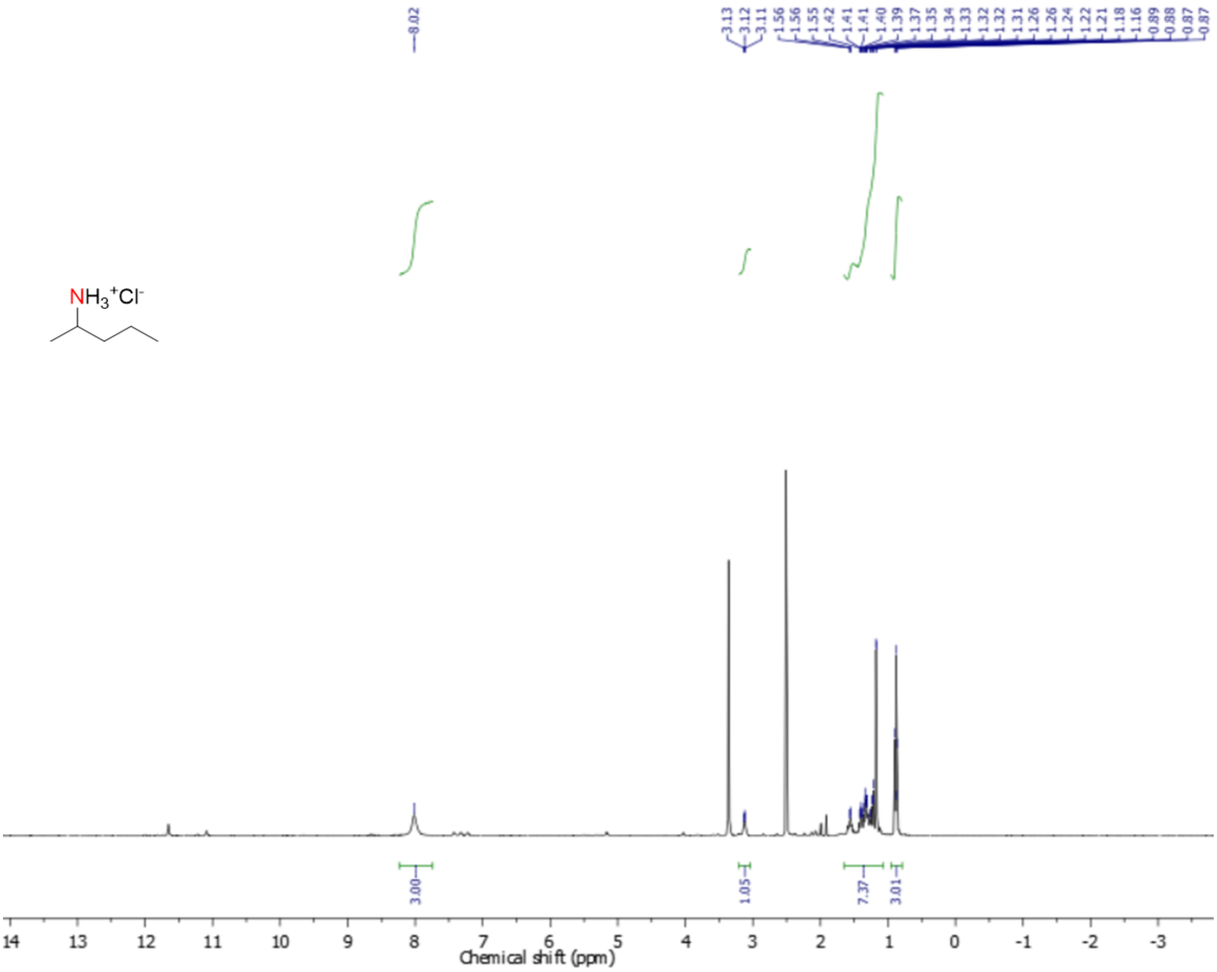
8.02
3.13
3.12
3.11
1.56
1.56
1.55
1.42
1.41
1.41
1.40
1.39
1.37
1.35
1.34
1.33
1.32
1.32
1.31
1.26
1.26
1.24
1.22
1.21
1.18
1.16
0.89
0.88
0.87
0.87
NH3+Cl-
3.00
1.05
7.37
3.01
Chemical shift (ppm)

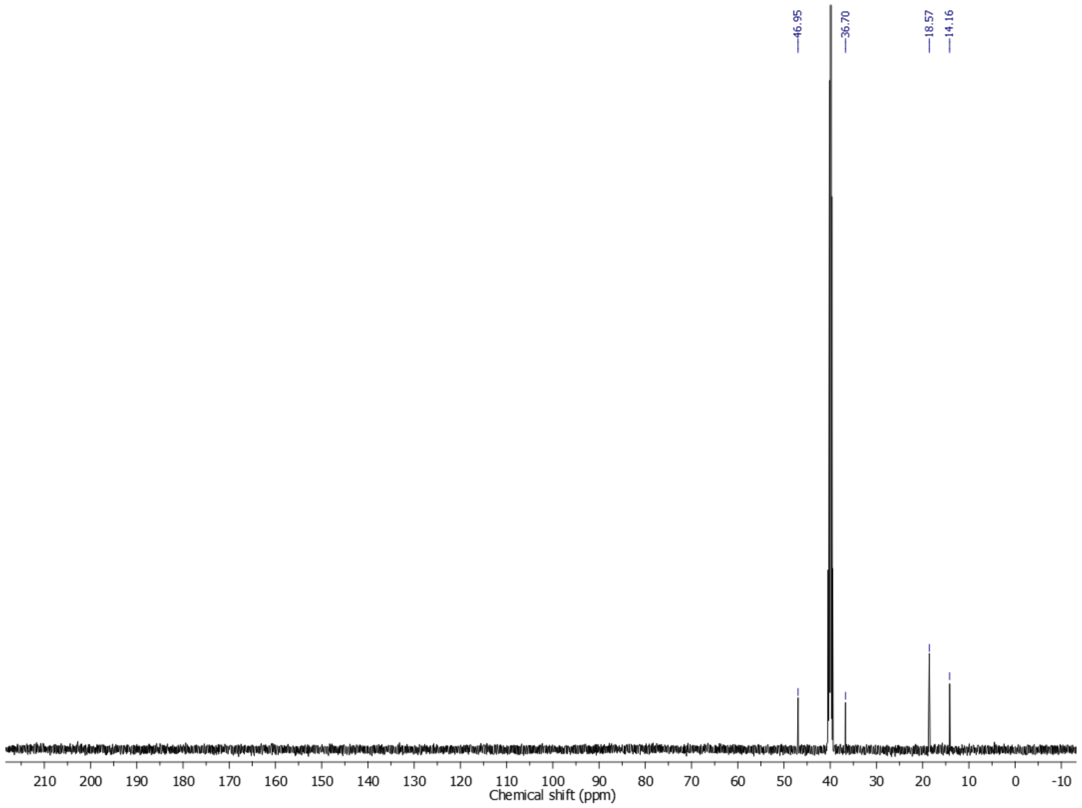
46.95
36.70
18.57
14.16
Chemical shift (ppm)

25:

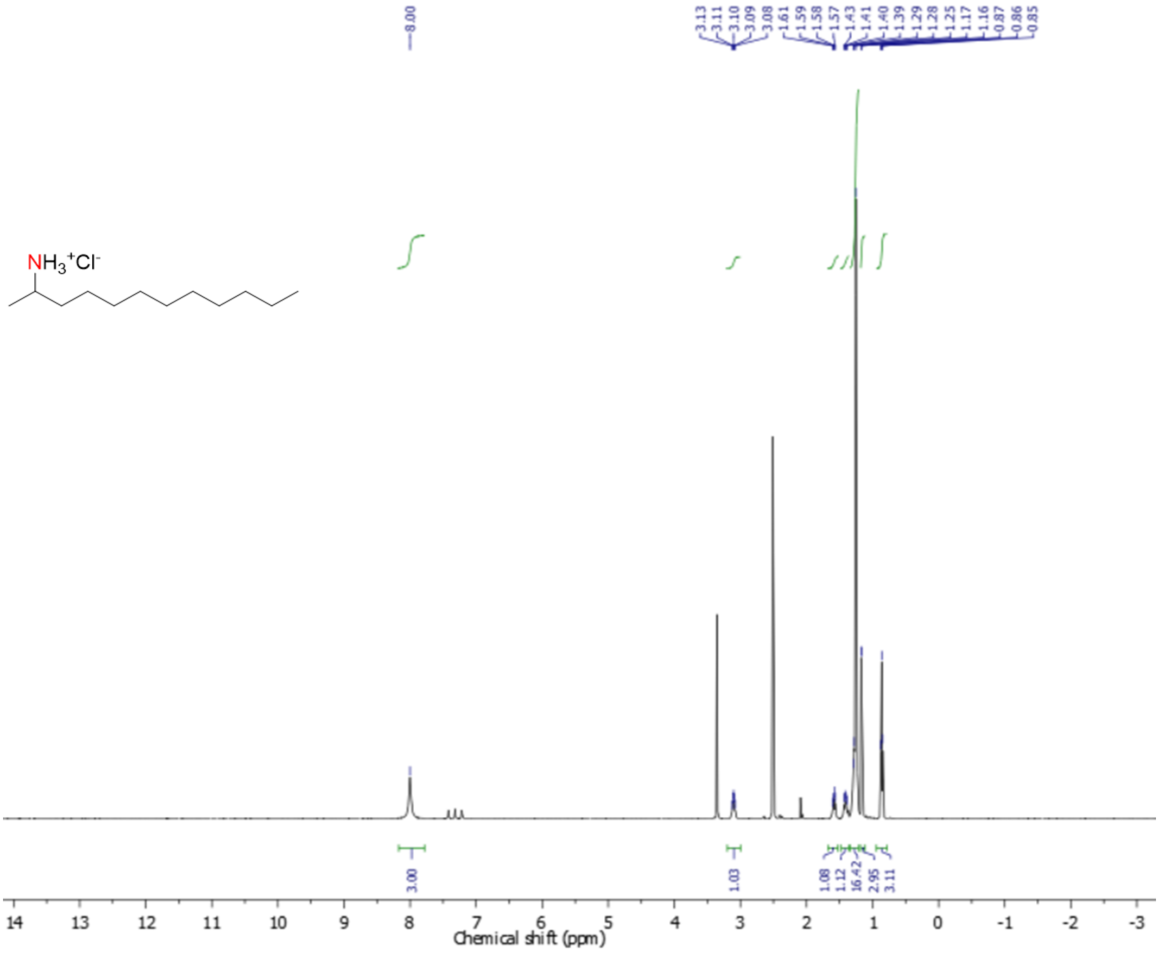
8.00
3.13
3.11
3.10
3.09
3.08
1.61
1.59
1.58
1.57
1.43
1.41
1.40
1.39
1.29
1.28
1.25
1.17
1.16
0.87
0.86
0.85
NH3+Cl-
3.00
1.03
1.08
1.12
16.42
2.95
3.11
Chemical shift (ppm)

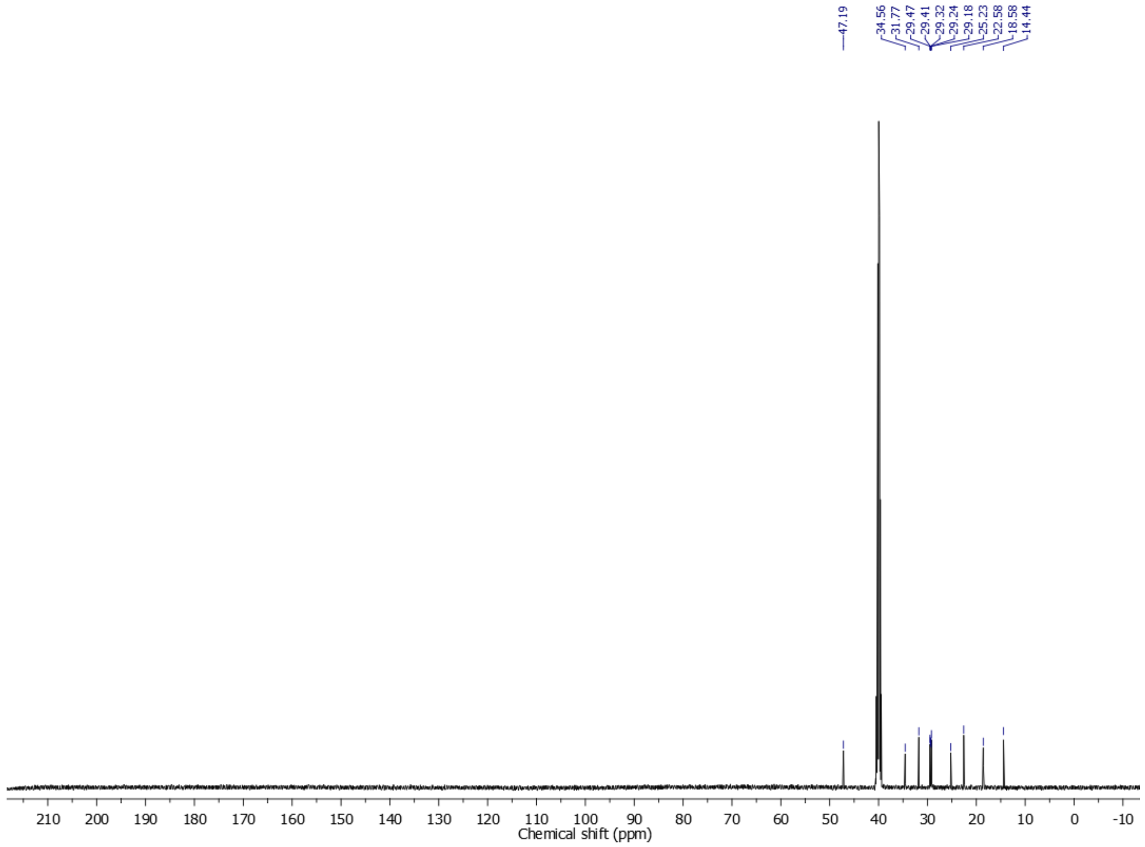
47.19
34.56
31.77
29.47
29.41
29.32
29.24
29.18
25.23
22.58
18.58
14.44
Chemical shift (ppm)

26:

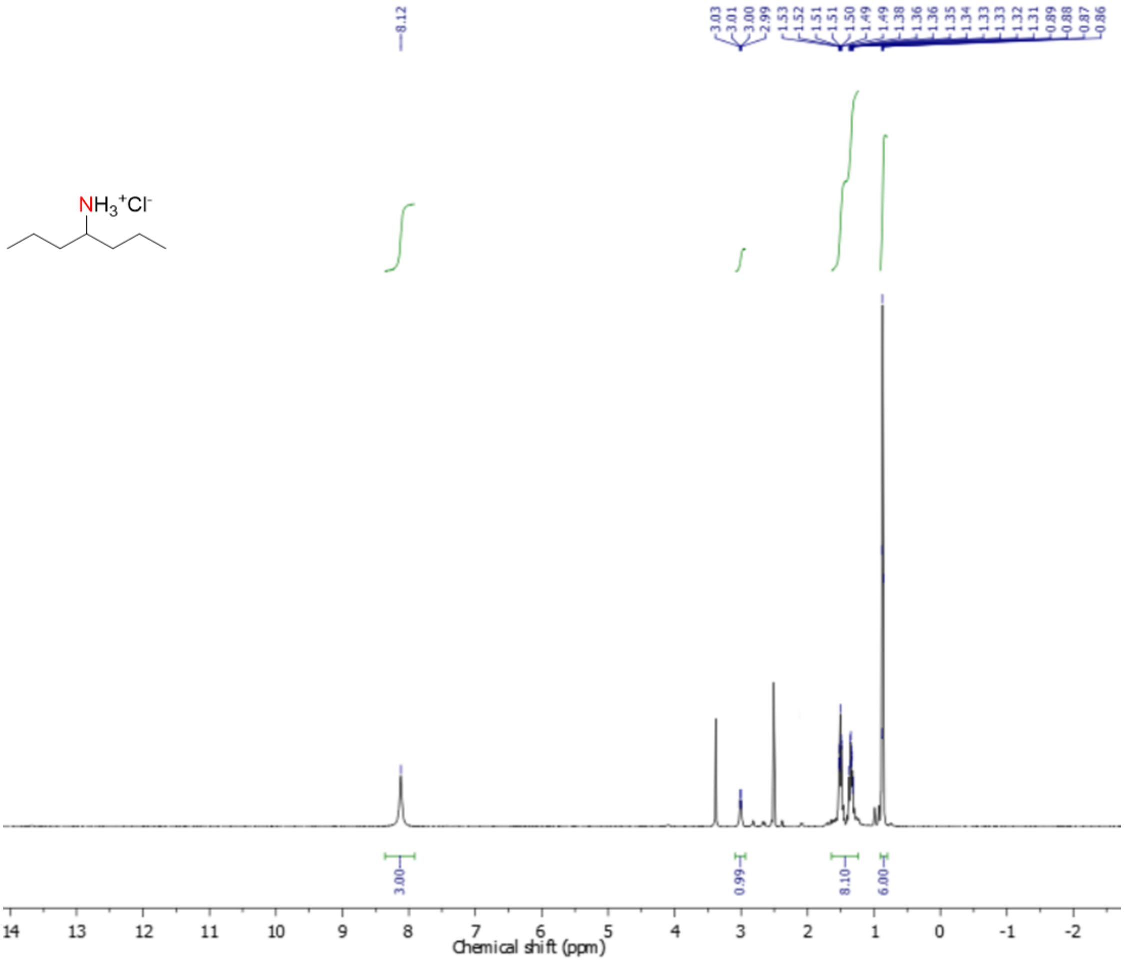
NH3+Cl-
8.12
3.03
3.01
3.00
2.99
1.53
1.52
1.51
1.51
1.50
1.49
1.49
1.38
1.36
1.36
1.35
1.34
1.33
1.33
1.32
1.31
0.89
0.88
0.87
0.86
3.00
0.99
8.10
6.00
Chemical shift (ppm)

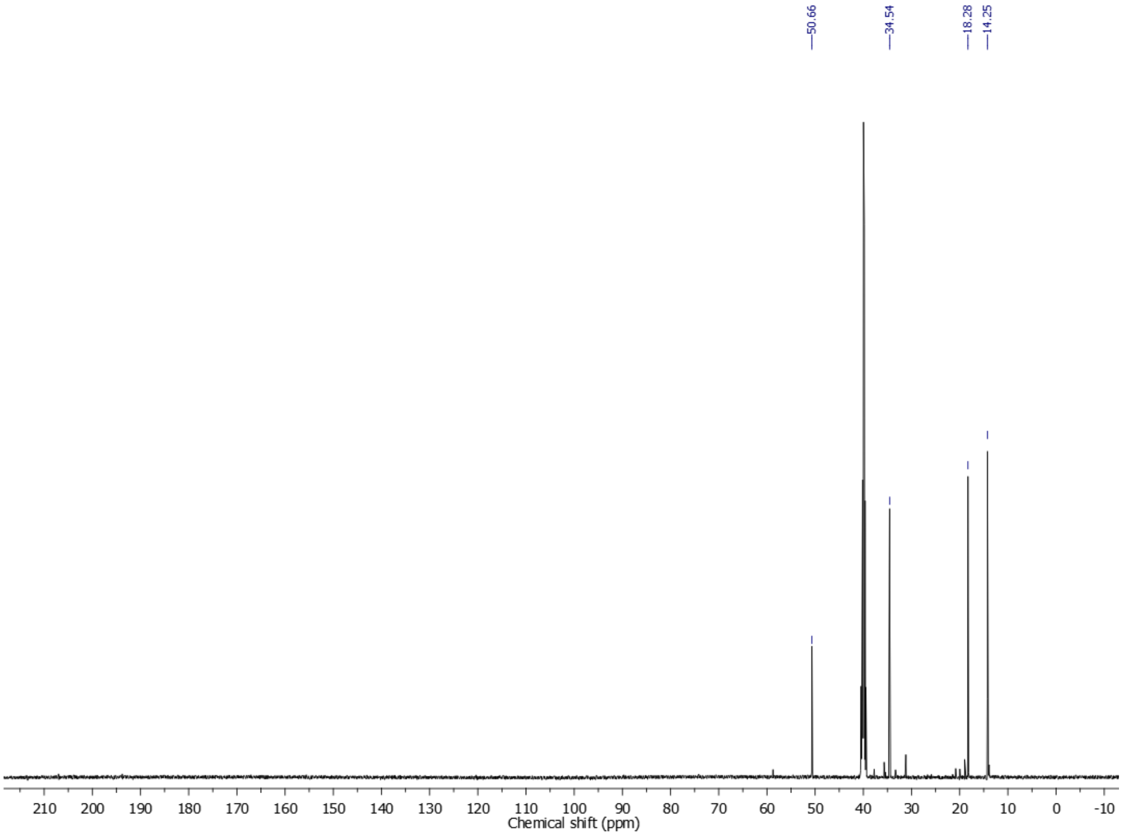
50.66
34.54
18.28
14.25
Chemical shift (ppm)

27:

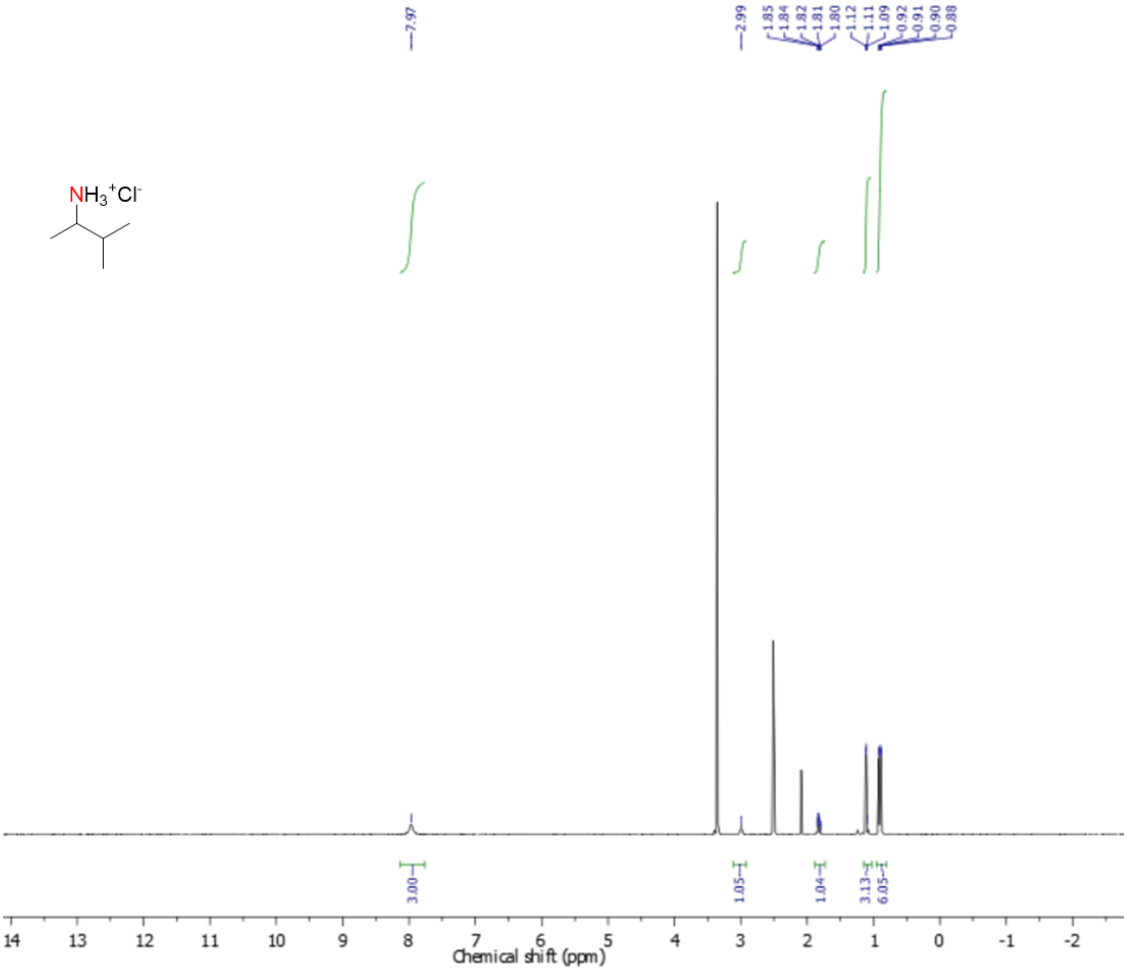
NH3+Cl-
7.97
2.99
1.85
1.84
1.82
1.81
1.80
1.12
1.11
1.09
0.92
0.91
0.90
0.88
3.00
1.05
1.04
3.13
6.05
Chemical shift (ppm)

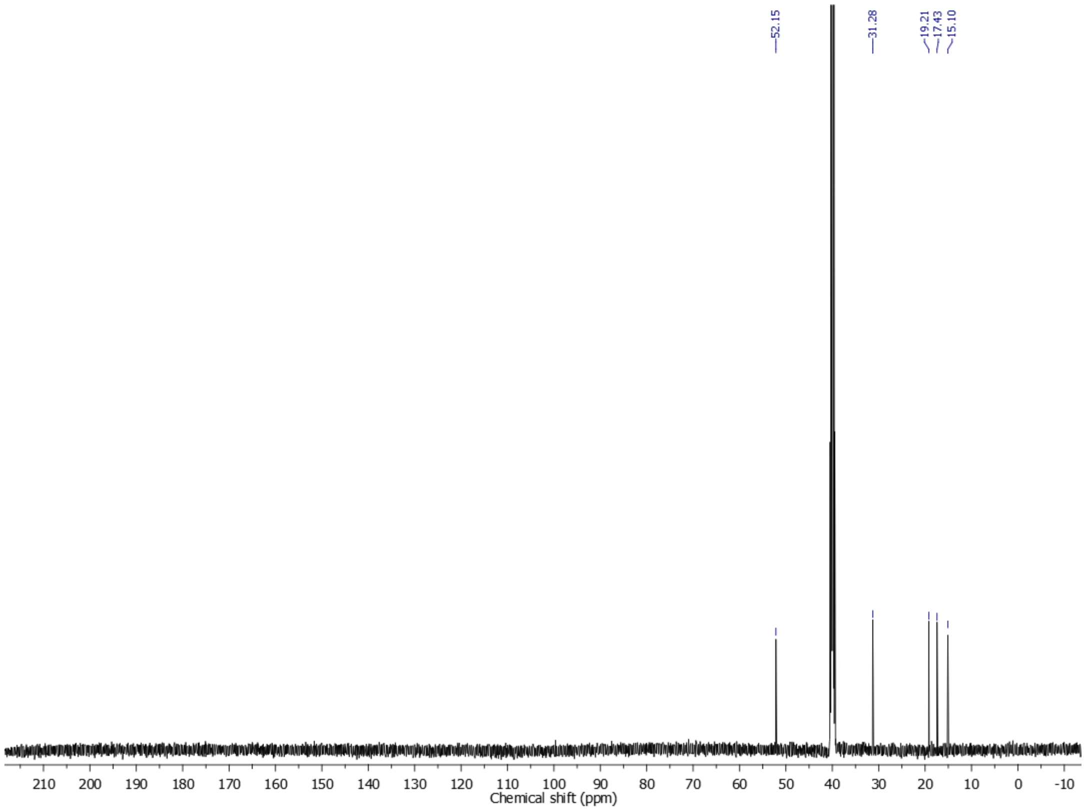
52.15
31.28
19.21
17.43
15.10
Chemical shift (ppm)

28:

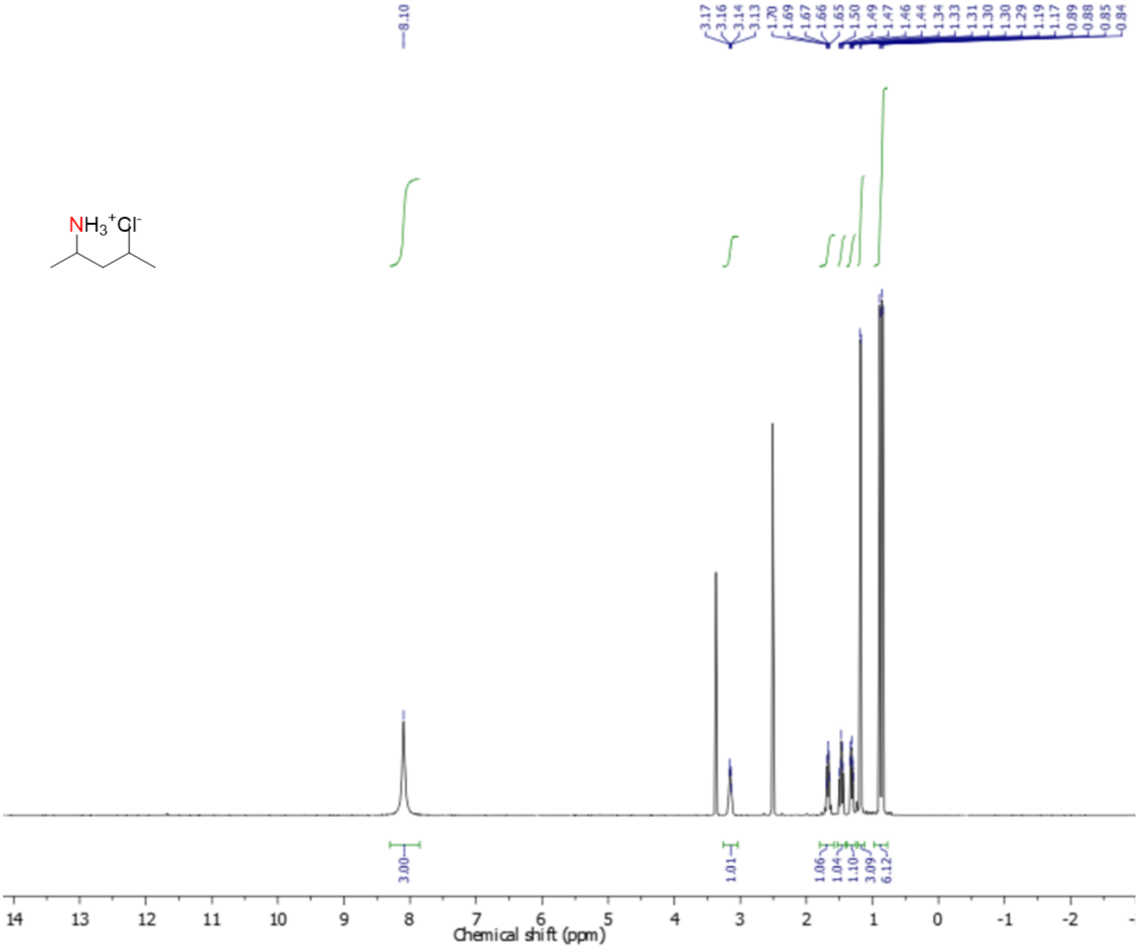
NH3+Cl-
8.10
3.17
3.16
3.14
3.13
1.70
1.69
1.67
1.66
1.65
1.50
1.49
1.47
1.46
1.44
1.34
1.33
1.31
1.30
1.30
1.29
1.19
1.17
0.89
0.88
0.85
0.84
3.00
1.01
1.06
1.04
1.10
3.09
6.12
Chemical shift (ppm)

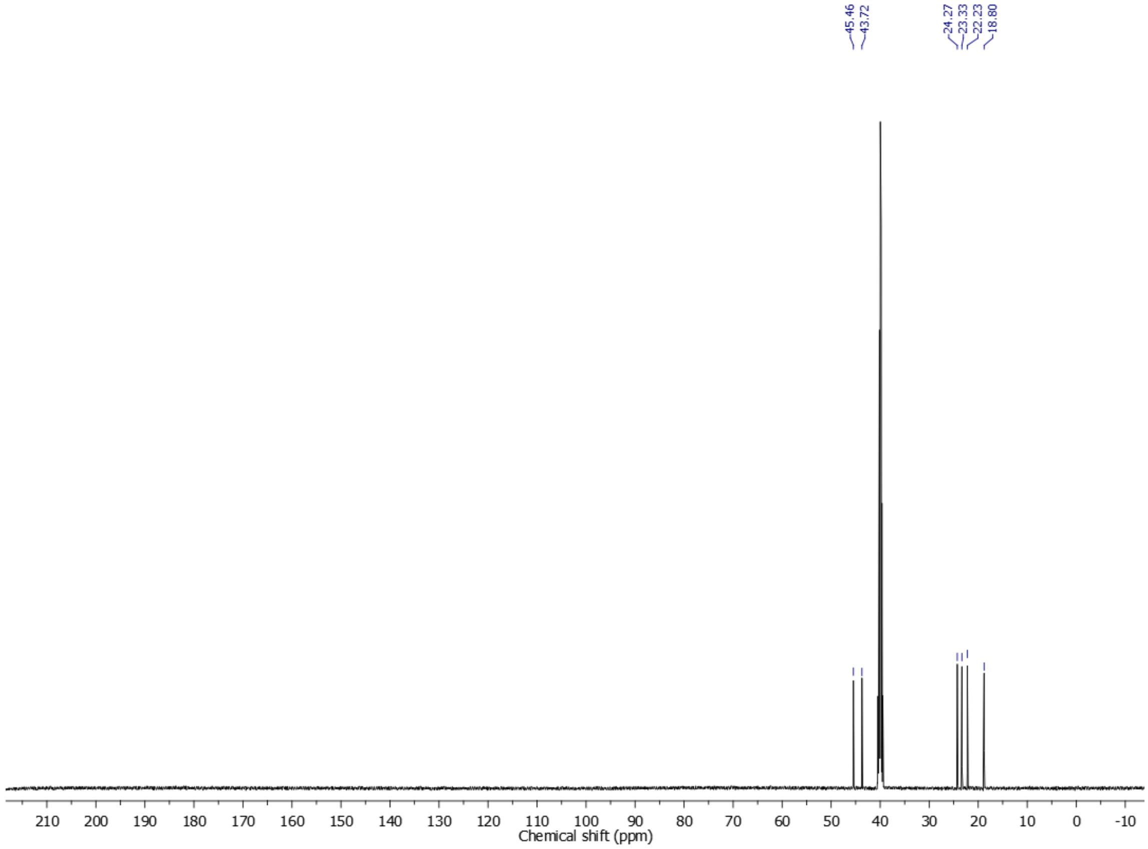
45.46
43.72
24.27
23.33
22.23
18.80
Chemical shift (ppm)

29:

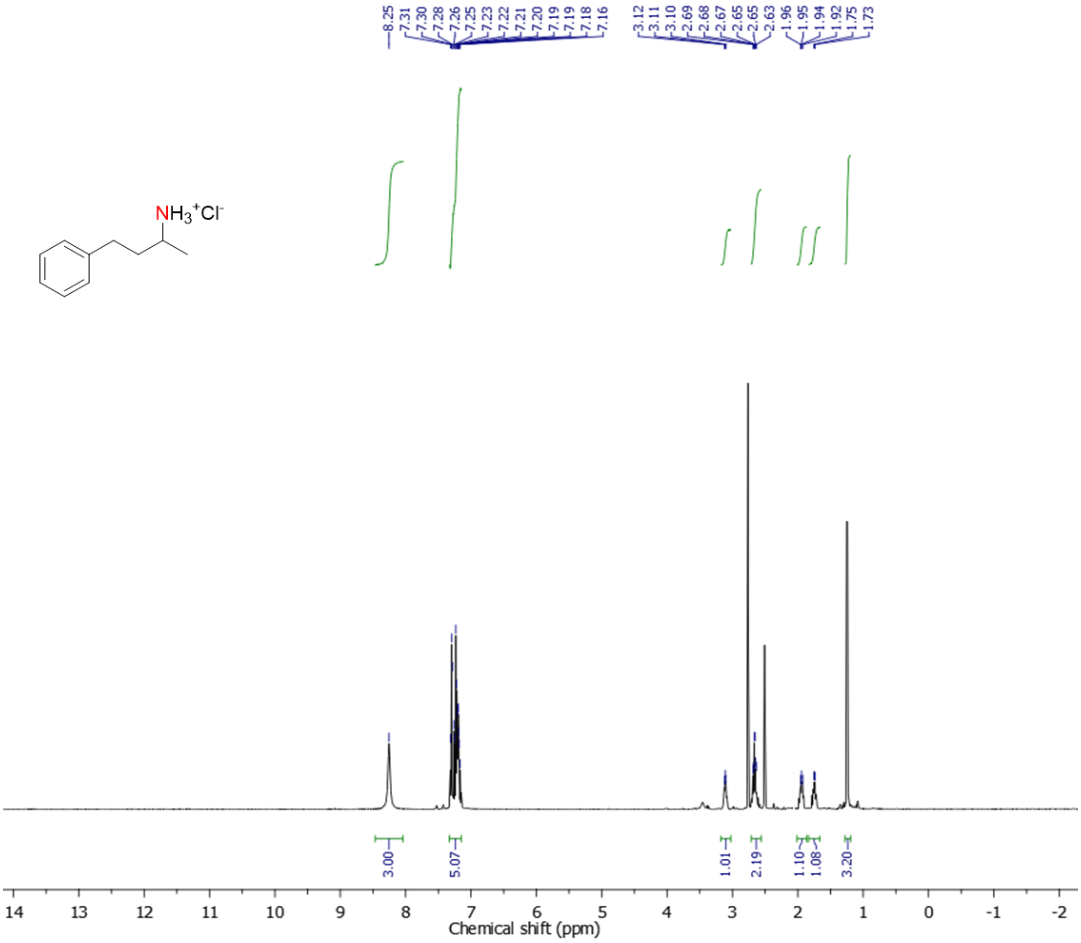

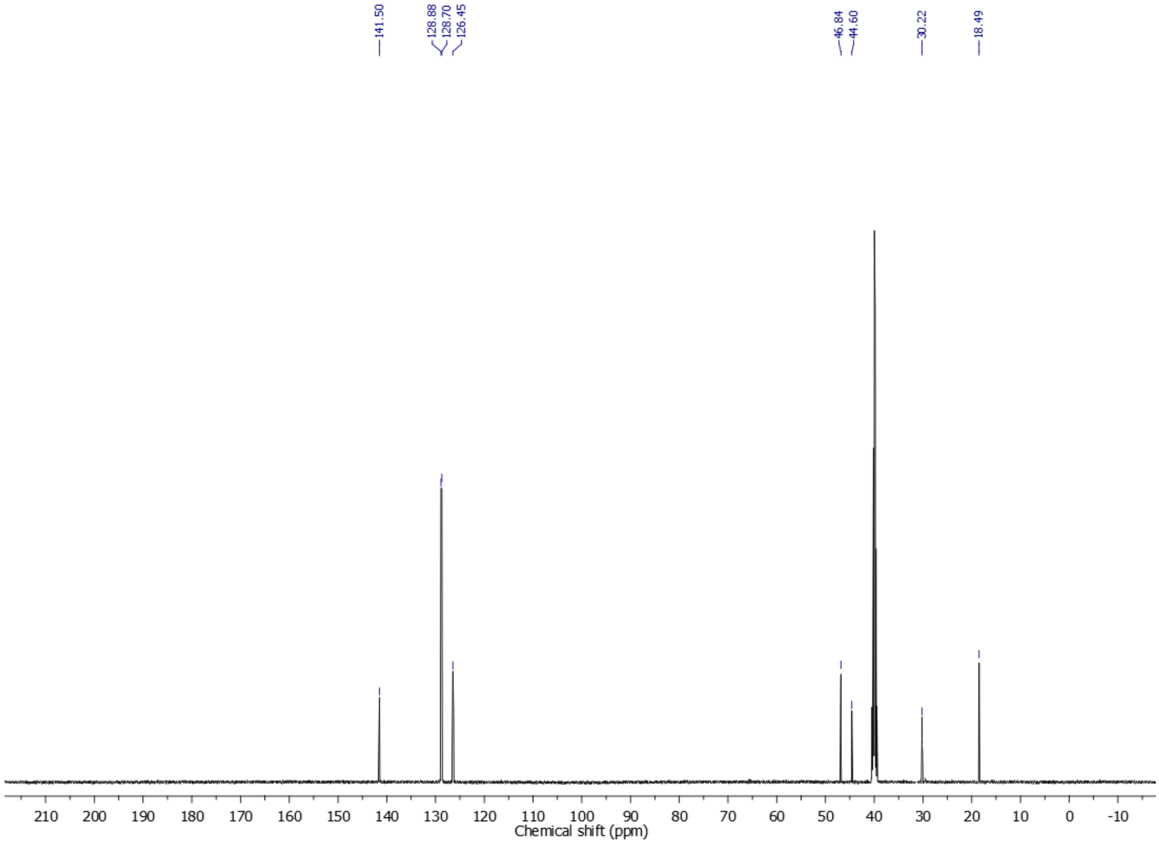

30:

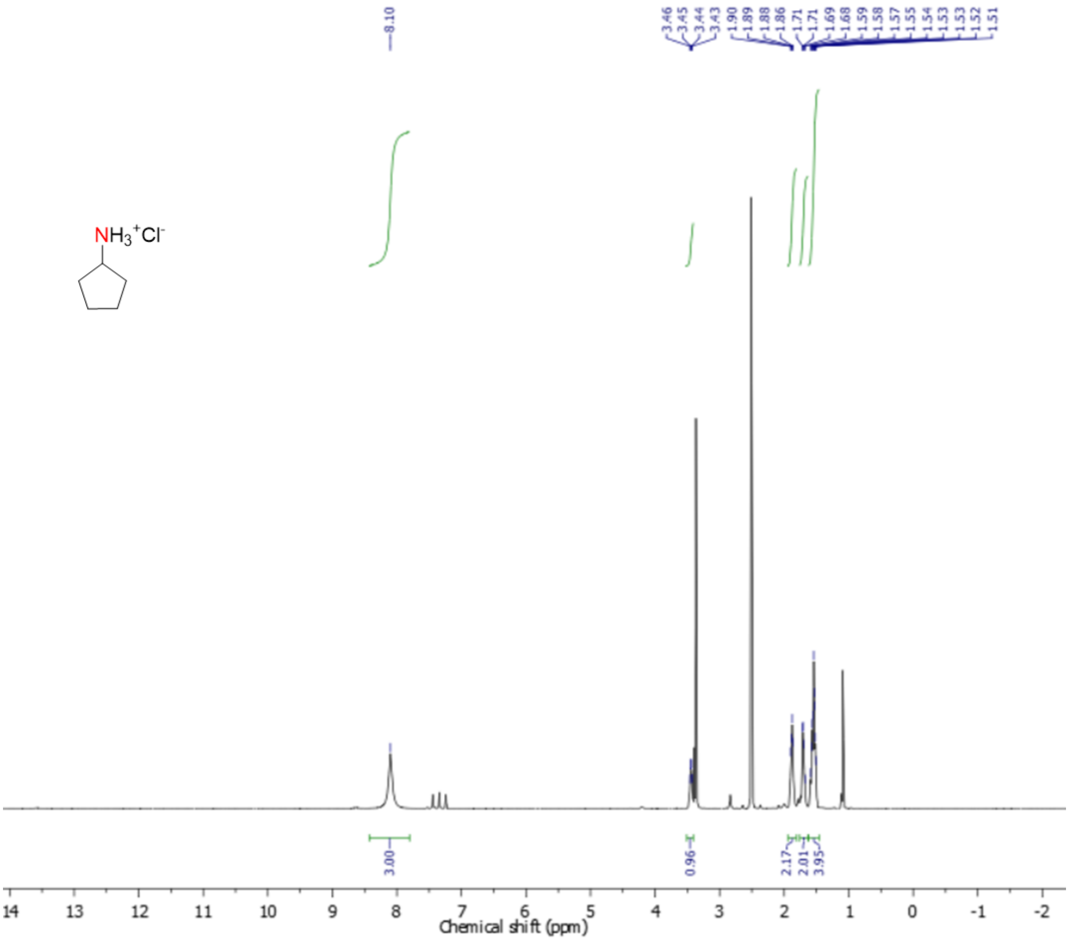
NH3+Cl-
8.10
3.46
3.45
3.44
3.43
1.90
1.89
1.88
1.86
1.71
1.71
1.69
1.68
1.59
1.58
1.57
1.55
1.54
1.53
1.53
1.52
1.51
3.00
0.96
2.17
2.01
3.95
Chemical shift (ppm)

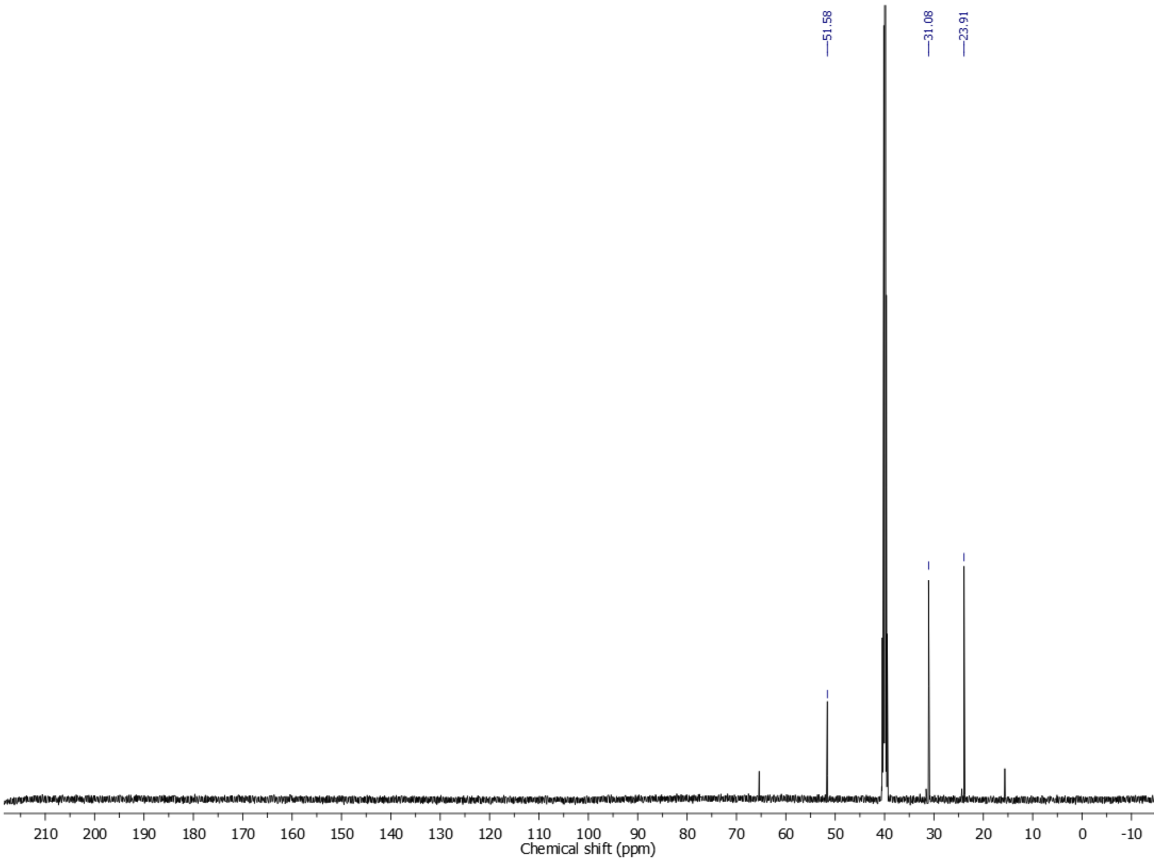
51.58
31.08
23.91
Chemical shift (ppm)

31:

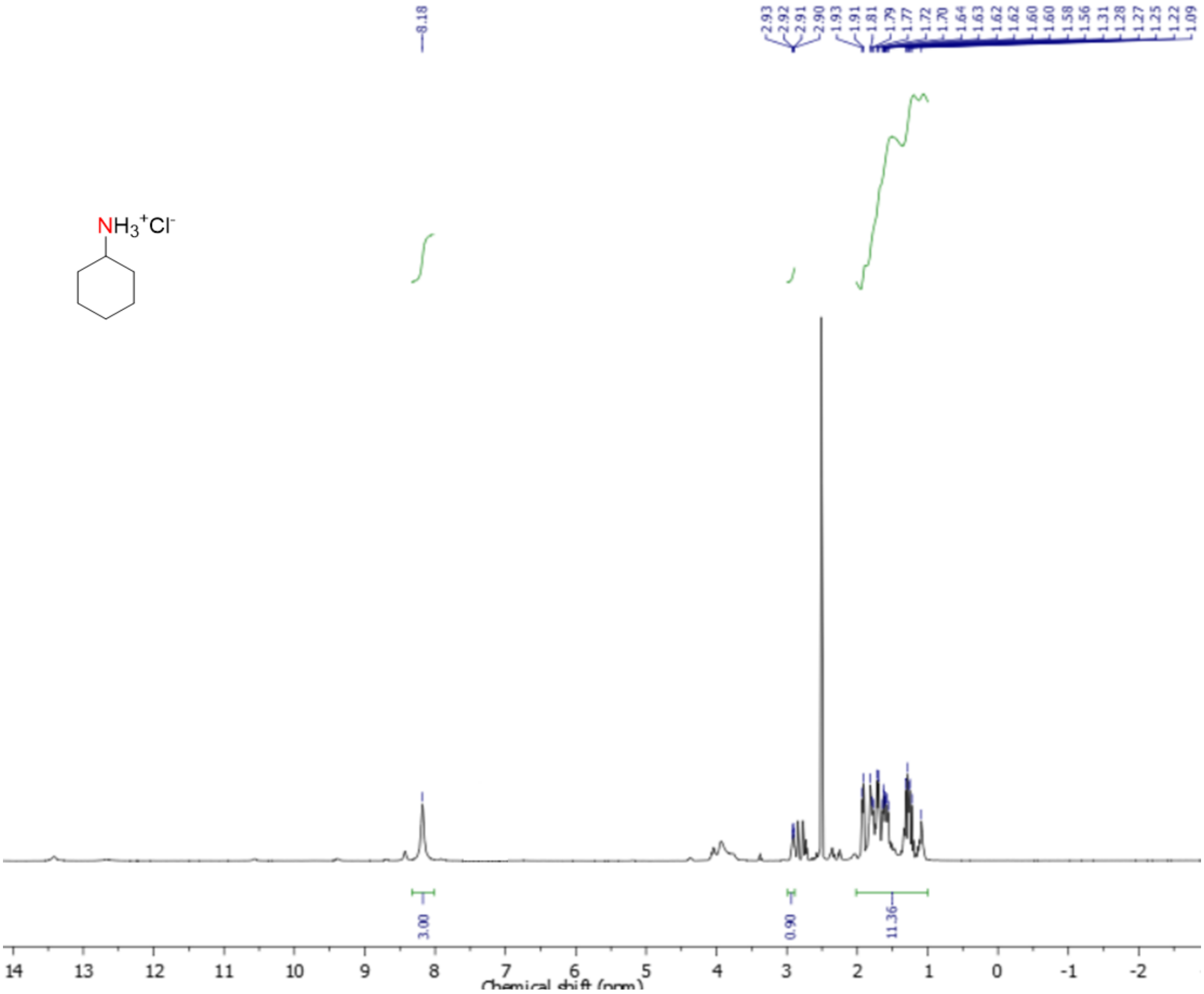
NH3+Cl-
8.18
2.93
2.92
2.91
2.90
1.93
1.91
1.81
1.79
1.77
1.72
1.70
1.64
1.63
1.62
1.62
1.60
1.60
1.58
1.56
1.31
1.28
1.27
1.25
1.22
1.09
3.00
0.90
11.36
Chemical shift (ppm)

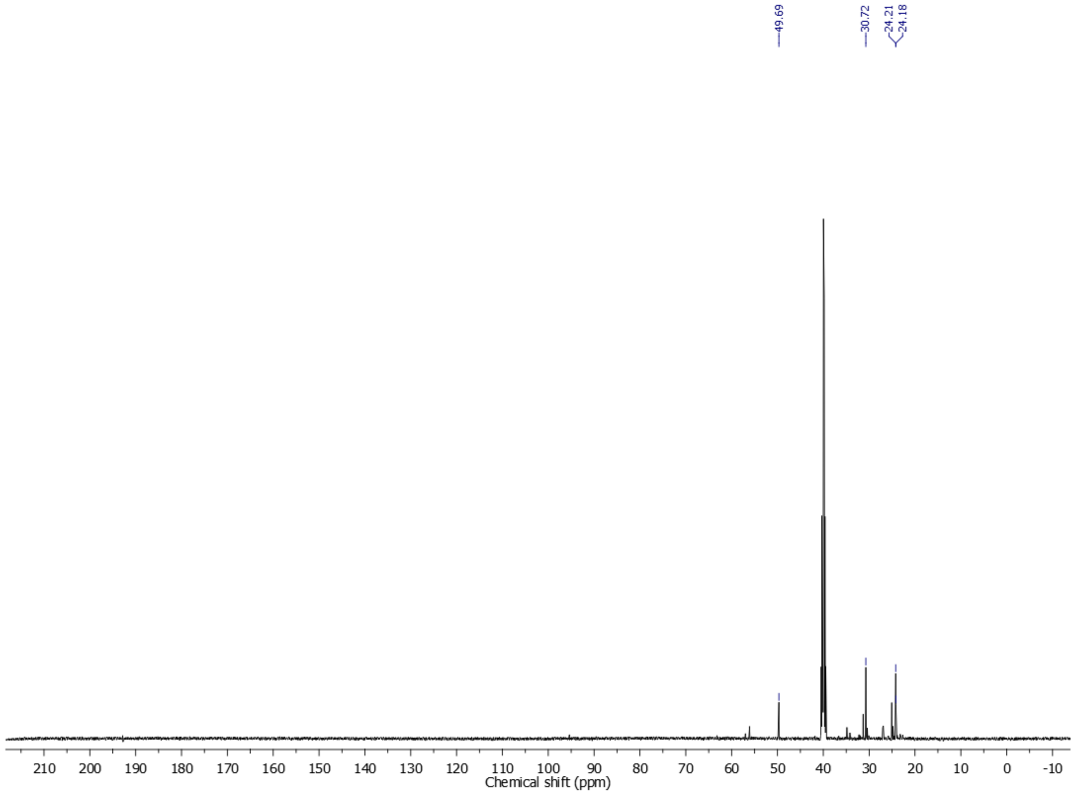
49.69
30.72
24.21
24.18
Chemical shift (ppm)

32:

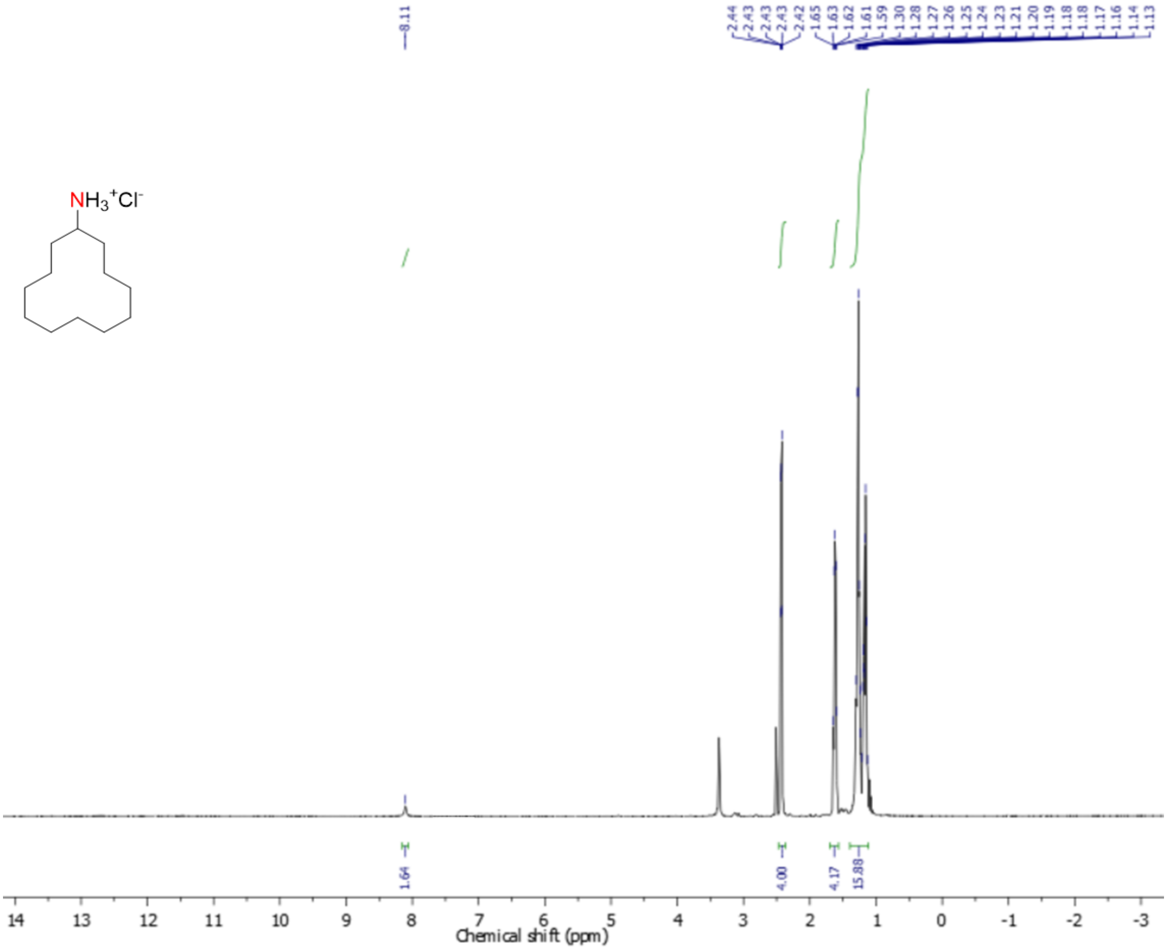
NH3+Cl-
8.11
2.44
2.43
2.43
2.43
2.42
1.65
1.63
1.62
1.61
1.59
1.30
1.28
1.27
1.26
1.25
1.24
1.23
1.21
1.20
1.19
1.18
1.18
1.17
1.16
1.14
1.13
1.64
4.00
4.17
15.88
Chemical shift (ppm)

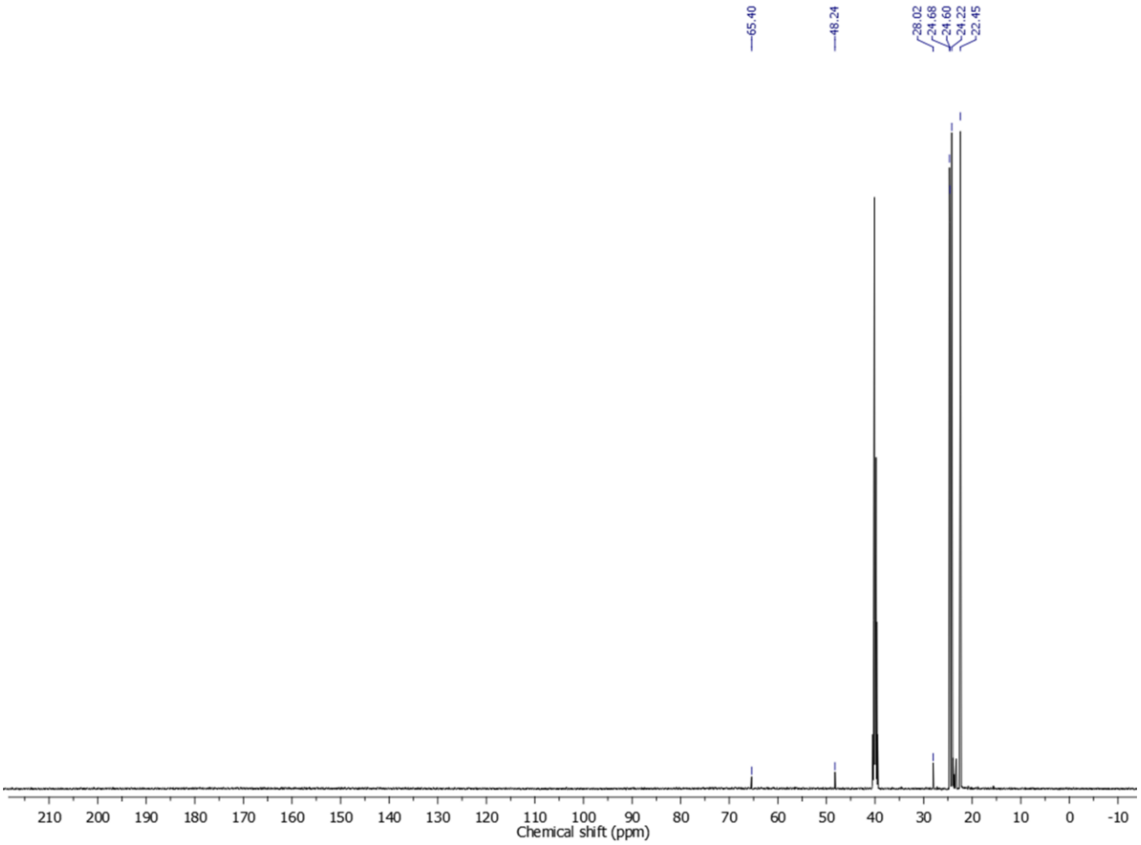
65.40
48.24
28.02
24.68
24.60
24.22
22.45
Chemical shift (ppm)

33:

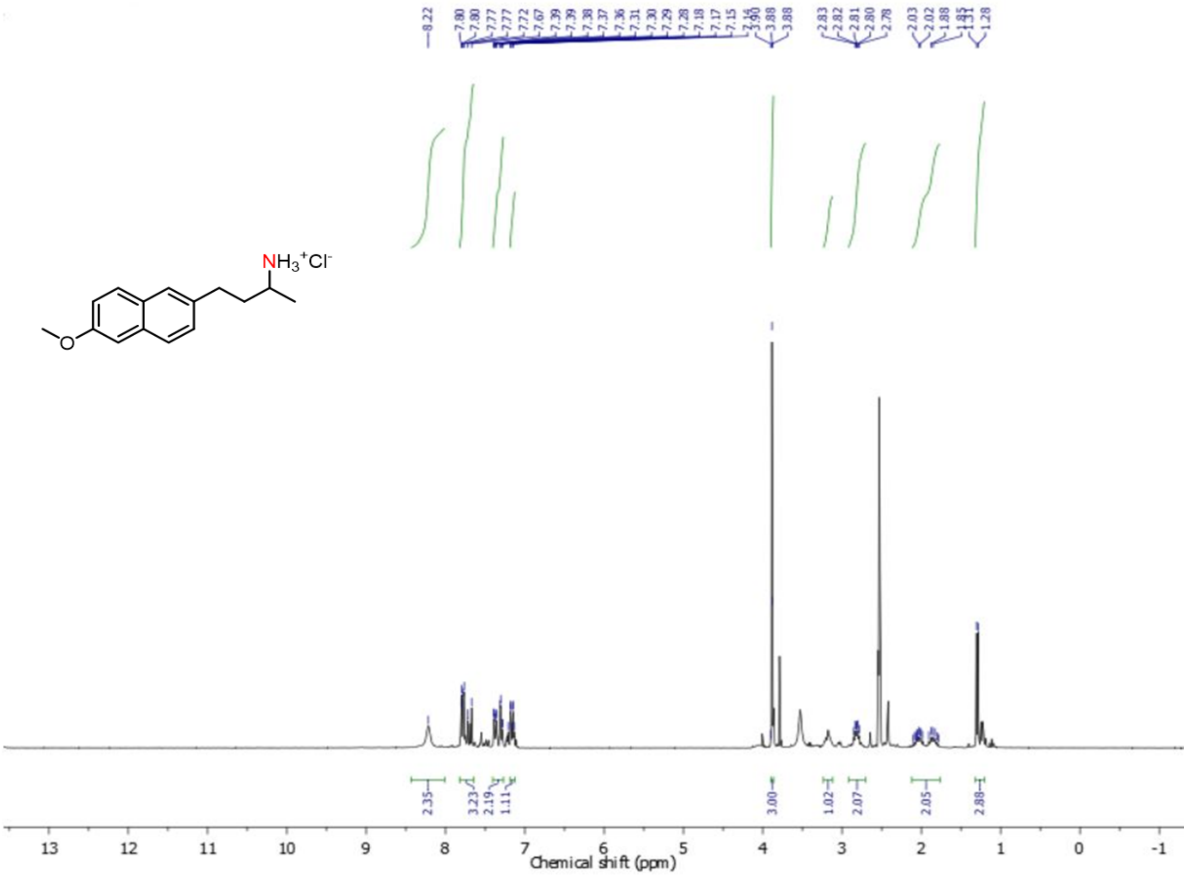

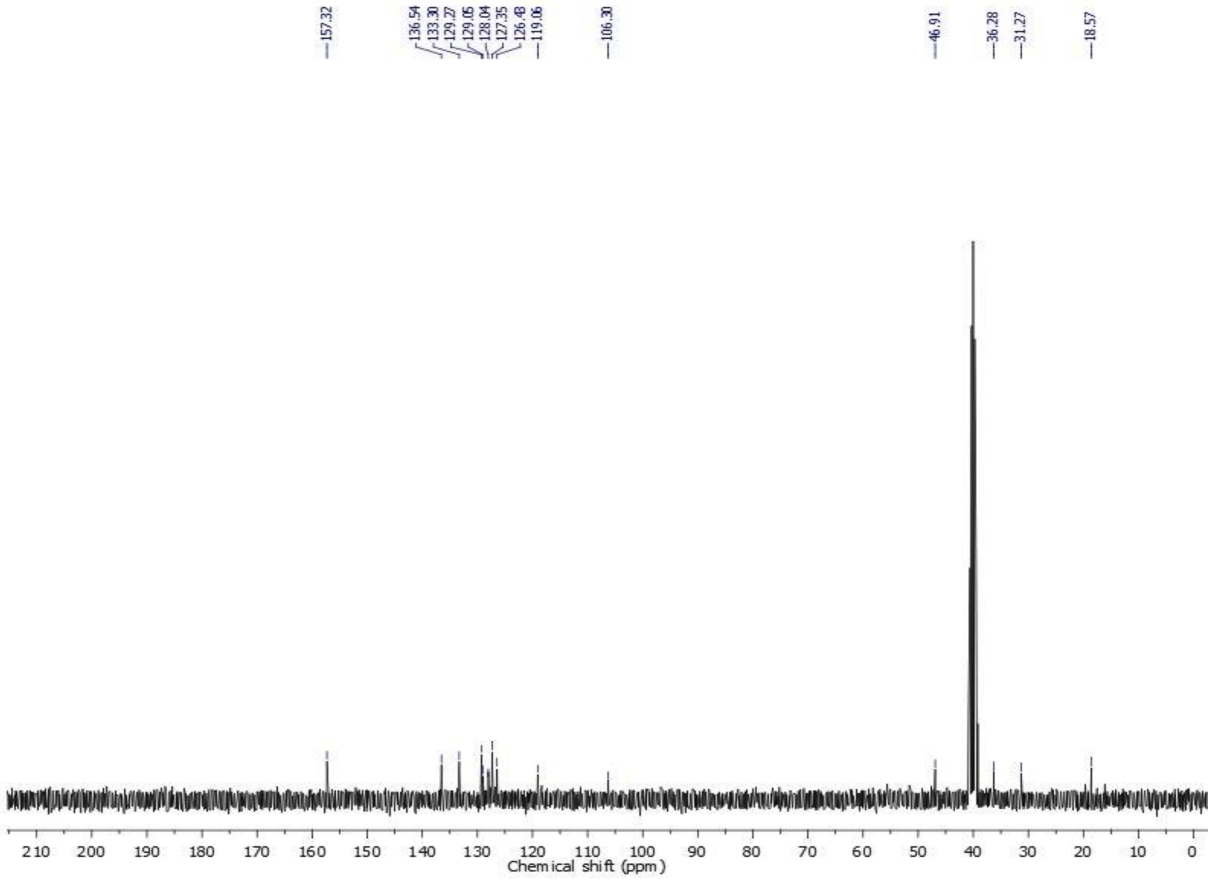

34:

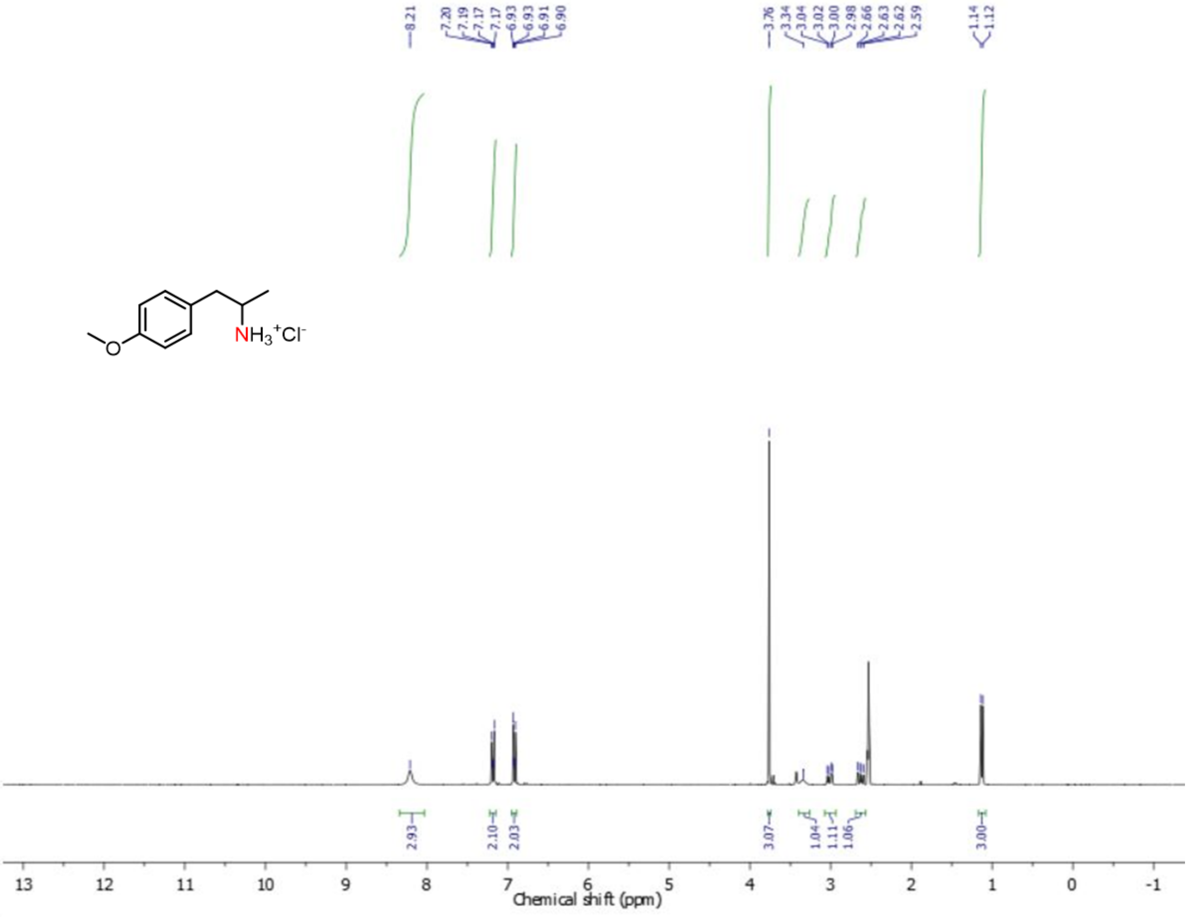

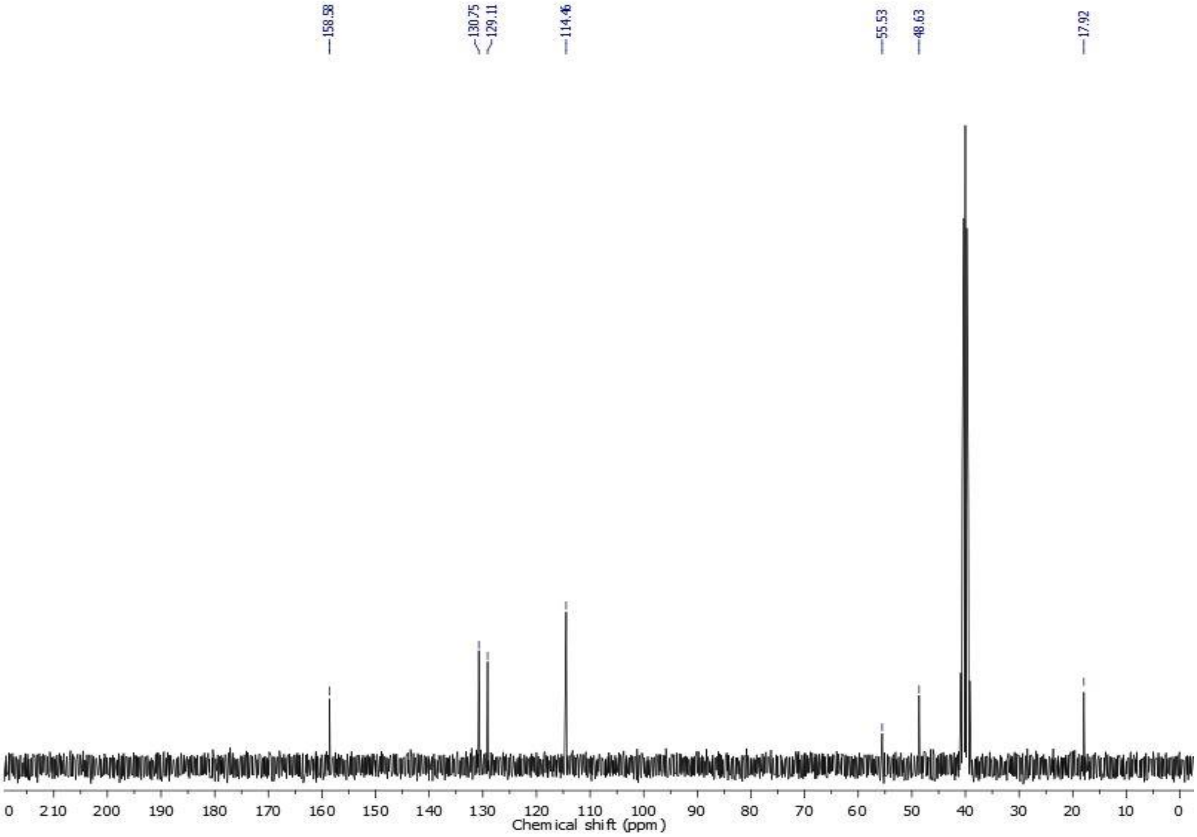

35:

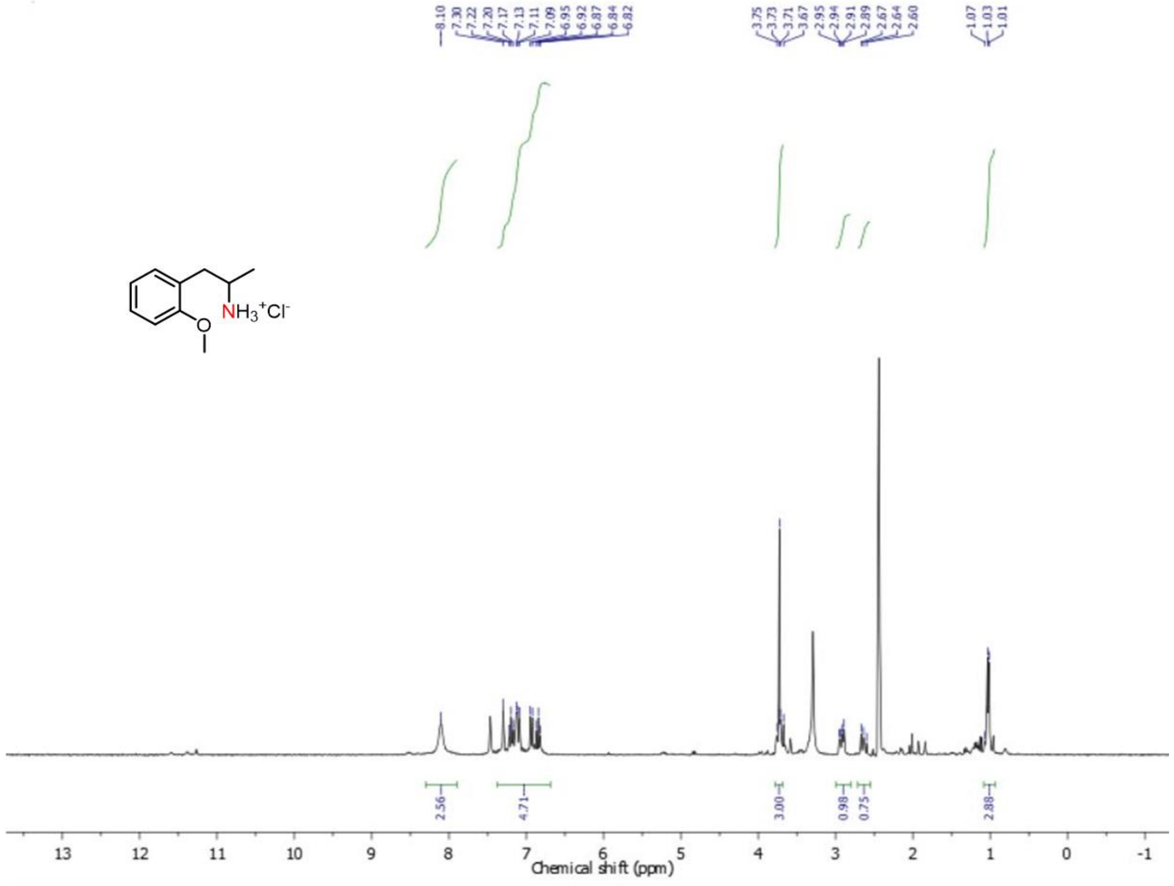
NH3+Cl-
O
8.10
7.30
7.22
7.20
7.17
7.13
7.11
7.09
6.95
6.92
6.87
6.84
6.82
3.75
3.73
3.71
3.67
2.95
2.94
2.91
2.89
2.67
2.64
2.60
1.07
1.03
1.01
2.56
4.71
3.00
0.98
0.75
2.88
Chemical shift (ppm)

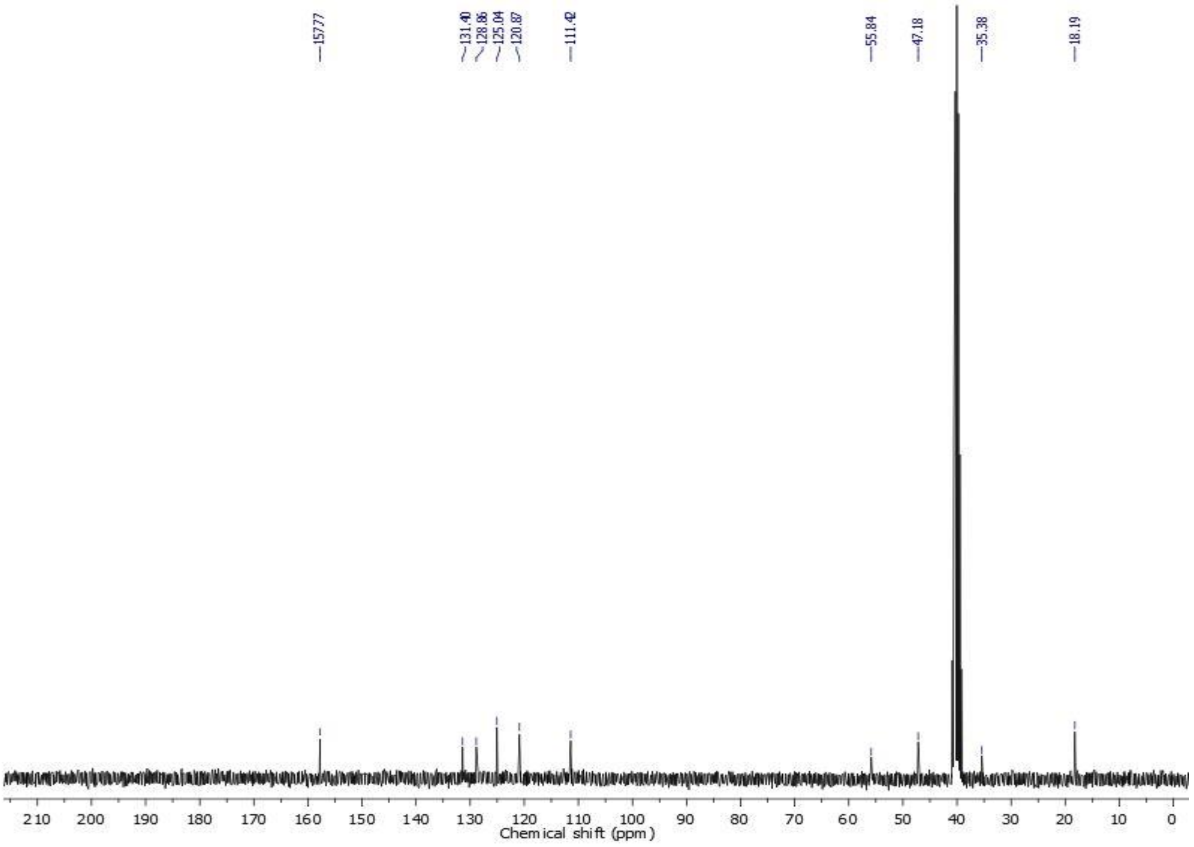
157.77
131.40
128.86
125.04
120.87
111.42
55.84
47.18
35.38
18.19
Chemical shift (ppm)

36:

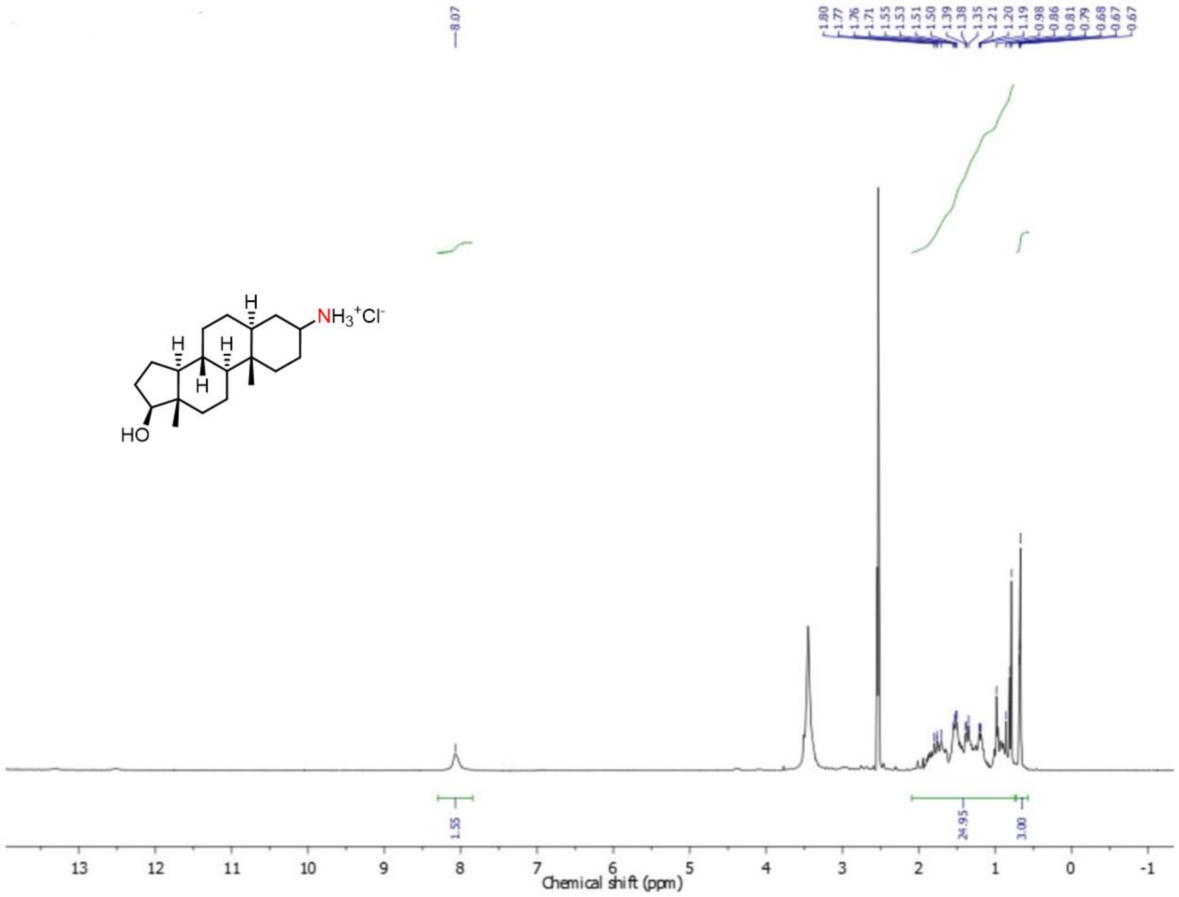
8.07
1.80 1.77 1.76 1.71 1.55 1.53 1.51 1.50 1.39 1.38 1.35 1.21 1.20 1.19 0.98 0.86 0.81 0.79 0.68 0.67 0.67
NH3+Cl-
HO
H
1.55
24.95
3.00
13 12 11 10 9 8 7 6 5 4 3 2 1 0 -1
Chemical shift (ppm)

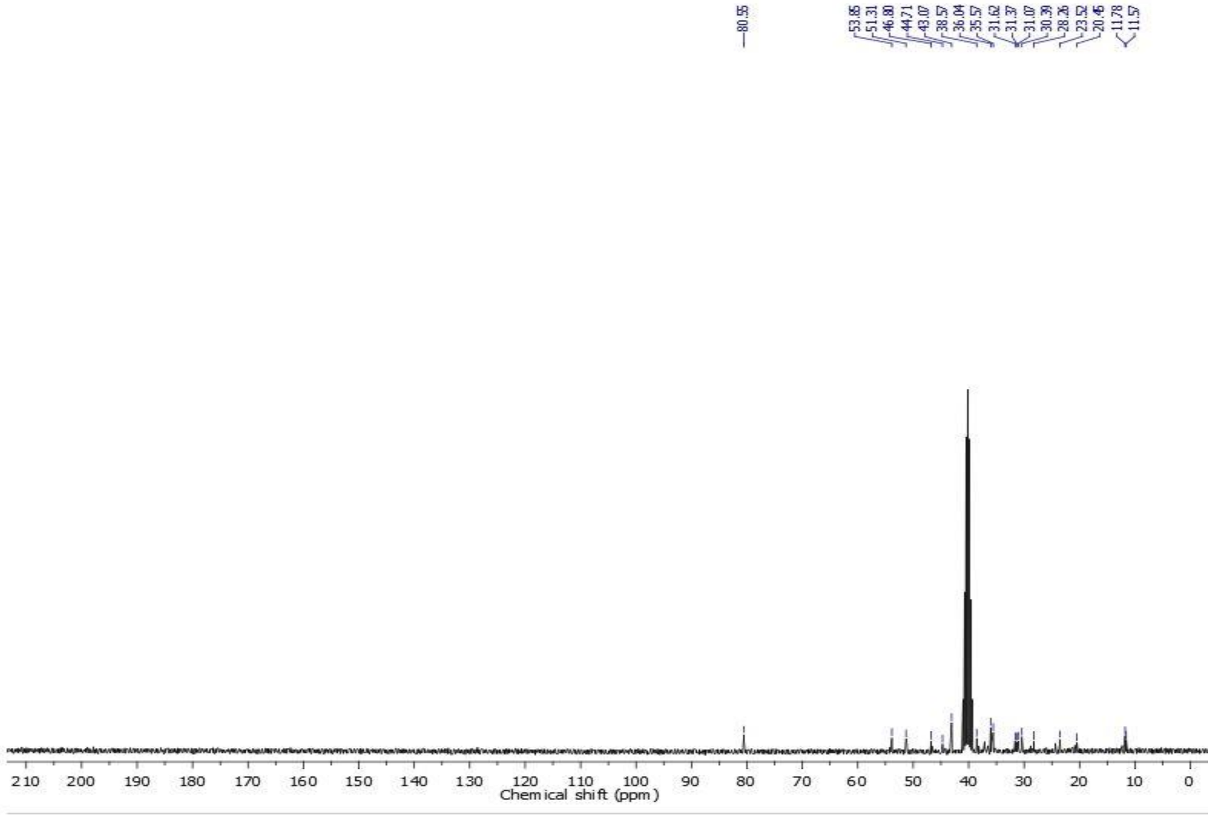
80.55
53.85 51.31 46.80 44.71 43.07 38.57 36.04 35.57 31.62 31.37 31.07 30.39 28.26 23.52 20.46 11.78 11.57
210 200 190 180 170 160 150 140 130 120 110 100 90 80 70 60 50 40 30 20 10 0
Chemical shift (ppm)

6. References

[1] G. Ashiotis, A. Deschildre, Z. Nawaz, J. P. Wright, D. Karkoulis, F. E. Picca, J. Kieffer, *J. Appl. Crystallogr.* **2015**, *48*, 510-519.
[2] xpdtools.
[3] P. Juhás, T. Davis, C. L. Farrow, S. J. L. Billinge, *J. Appl. Crystallogr.* **2013**, *46*, 560-566.
[4] C. L. Farrow, P. Juhas, J. W. Liu, D. Bryndin, E. S. Bozin, J. Bloch, T. Proffen, S. J. Billinge, *J. Phys. Condens. Matter.* **2007**, *19*, 335219.
[5] J. Zemann, *Acta Crystallographica* **1965**, *18*, 139-139.
[6] J. D. Hanawalt, H. W. Rinn, L. K. Frevel, *Ind. Eng. Chem. Res.* **1938**, *10*, 457-512.
[7] L. F. B. Ribeiro, O. Flores, P. Furtat, C. Gervais, R. Kempe, R. A. F. Machado, G. Motz, *J. Mater. Chem. A* **2017**, *5*, 720-729.
[8] a) A. Poulain, C. Dupont, P. Martinez, C. Guizani, J. Drnec, *J. Appl. Crystallogr.* **2019**, *52*, 60-71; b) V. Petkov, R. G. Difrancesco, S. J. L. Billinge, M. Acharya, H. C. Foley, *Philosophical Magazine B* **2009**, *79*, 1519-1530.
[9] A. C. Forse, C. Merlet, P. K. Allan, E. K. Humphreys, J. M. Griffin, M. Aslan, M. Zeiger, V. Presser, Y. Gogotsi, C. P. Grey, *Chem. Mater.* **2015**, *27*, 6848-6857.
[10] J. M. Stratford, P. K. Allan, O. Pecher, P. A. Chater, C. P. Grey, *Chem. Commun.* **2016**, *52*, 12430-12433.
[11] C. Bommier, T. W. Surta, M. Dolgos, X. Ji, *Nano Lett.* **2015**, *15*, 5888-5892.
[12] M. G. Tucker, M. T. Dove, D. A. Keen, *J. Phys. Condens. Matter.* **2000**, *12*, L425-L430.
[13] A. Pines, M. G. Gibby, J. S. Waugh, *J. Chem. Phys*. **1972**, *56*, 1776
[14] B. M. Fung, A. K. Khitrin, K. Ermolaev, *J. Magn. Reson*, **2000**, *142*, 97-101
[15] Z. Gan*, J. Am. Chem. Soc.* **2006**, *128*, 6040-6041
[16] S. Cavadini, A. Lupulescu, S. Antonijevic, G. Bodenhausen, *J. Am. Chem. Soc*. **2006**, *128*, 7706-7707
[17] A. Brinkmann, A. P. M. Kentgens*, J. Am. Chem. Soc.* **2006**, *128*, 14758-14759

Author Contributions

M. E. carried out the catalyst synthesis, catalytic reactions and catalyst characterization. S. L. J. T. and M. Z. performed XRD analyses and PDF refinement. T. S. and M. E. co-developed the catalyst. R. S. and J. S. carried out solid state NMR analyses. M. D. recorded the ‘TEM images. M. E. and R. K. co-wrote the manuscript.
